# Supplementary material for: Genomic surveillance of SARS-CoV-2 Omicron variants on a university campus
Source: Nat Commun. 2022 Sep 6;13:5240. doi: 10.1038/s41467-022-32786-z (PMC9446629; doi:10.1038/s41467-022-32786-z)
Supplement: Supplementary file 1 — Supplementary Information [file 41467_2022_32786_MOESM1_ESM.pdf]

Manuscript: Genomic surveillance of SARS-CoV-2 Omicron variants on a university campus

SUPPLEMENTAL MATERIALS

SUPPLEMENTAL TABLES

	Mean unadjusted difference in Orf1b Ct value (95% CI)	p-value±	Mean adjusted difference in Orf1b Ct value (95% CI)*	p-value±
**All Omicron positive individuals, adjusted for age, symptoms, and average RNase P gene value N=1,688 (BA.1 = 1664, BA.2 = 24)**				
Lineage (BA.1 vs. BA.2)	0.47 (-0.94, 1.88)	0.51	-0.04 (-1.34, 1.26)	0.95
Age (years)	-0.02 (-0.04, 0.002)	0.09	-0.01 (-0.03, 0.003)	0.11
Symptoms (symptomatic vs. asymptomatic)	-1.06 (-1.49, -0.63)	**$1.3X10^{-6}$**	-1.25 (-1.65, -0.85)	**$7.8X10^{-10}$**
Average RNase P gene value	0.38 (0.33, 0.42)	**$7.4X10^{-56}$**	0.38 (0.34, 0.43)	**$1.1X10^{-58}$**

Supplemental Table 1. **Cycle threshold comparisons by Omicron lineage (BA.1 vs. BA.2).**

*Mean adjusted differences estimated using multiple linear regression of average Orf1b Ct value on lineage (BA.1 vs. BA.2) adjusted for age, symptoms, and average RNase P gene value. Regression was restricted to Omicron cases detected using RHINOstic™ swabs (42 Omicron cases detected using US Cotton #3 swabs).

±Two-sided *t* statistic with significance level of 0.05.

**Characteristic, N (%)**	**Cluster**			p-value
	One	Two	Three	
Student	35 (100)	22 (100)	66 (100)	N/A
Male	7 (20.0)	13 (59.1)	27 (40.9)	**0.009**
Resident of on-campus housing	2 (5.70)	8 (36.4)	4 (6.10)	**0.001**
Fraternity or sorority member	25 (71.4)	7 (31.8)	46 (69.7)	**0.004**
Completion of COVID-19 vaccination primary series [2]	21 (60.0)	14 (63.6)	42 (63.6)	0.22

Supplemental Table 2. **Demographic characteristics of three Delta infection clusters**[1]**.**

[1]Proportions between clusters compared using Fisher's exact tests.

[2]Vaccination status unknown for 37.1%, 31.8%, and 34.8% of individuals in clusters 1, 2, and 3, respectively.

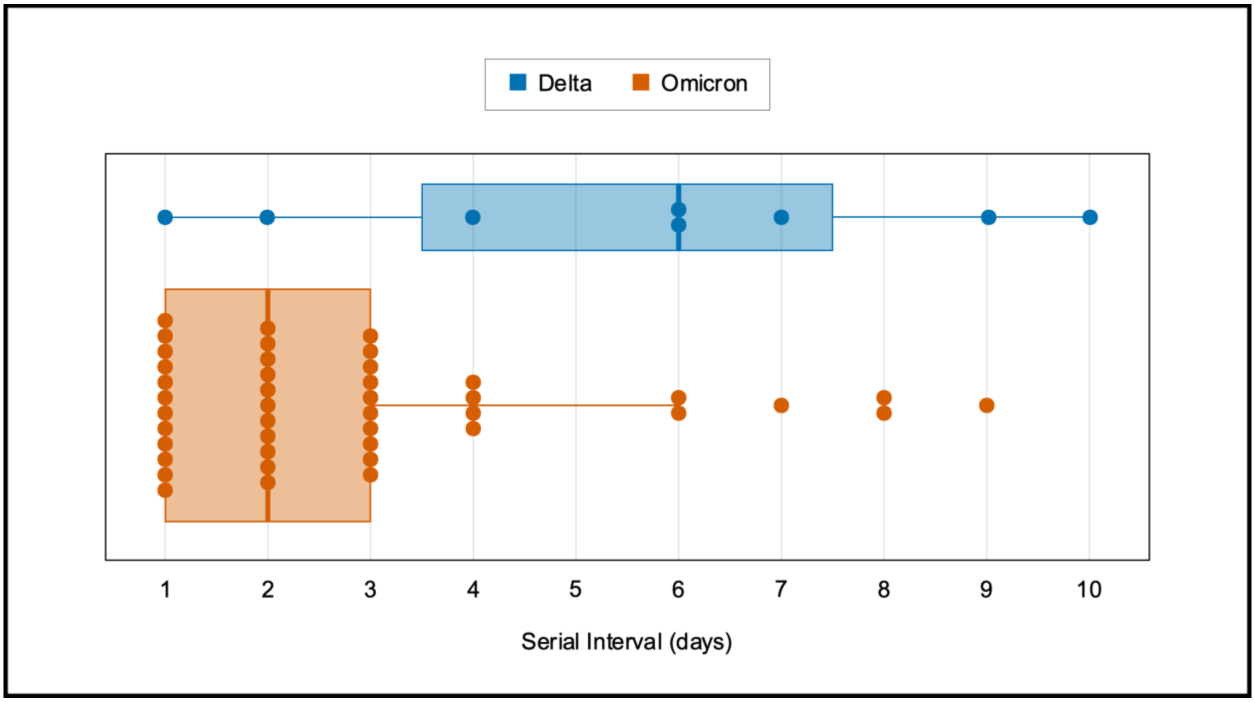

Supplemental Figure 1. **Serial intervals of Delta and Omicron transmission clusters**. Serial intervals are displayed for each subsequent case in symptomatic clusters of SARS-CoV-2 positive individuals with identical genomes and sharing the same address. The primary case of each cluster was identified by the earliest symptom onset date within the cluster. Serial interval was defined as the number of days between symptom onset of a symptomatic primary case and each subsequent symptomatic case within the cluster. Centerlines represent the median serial interval for each variant (Delta: 6 days, Omicron: 2 days), with boxes bound by the 25th and 75th percentiles (Delta: 3.5-7.5 days, Omicron: 1-3 days), and whiskers extending to 1.5 times the interquartile range below the 25th percentile (Delta: 1 day), and above the 75th percentile (Delta: 10 days, Omicron: 6 days). Minimum serial interval was 1 day for both variants; maximum was 10 days for Delta and 9 days for Omicron.

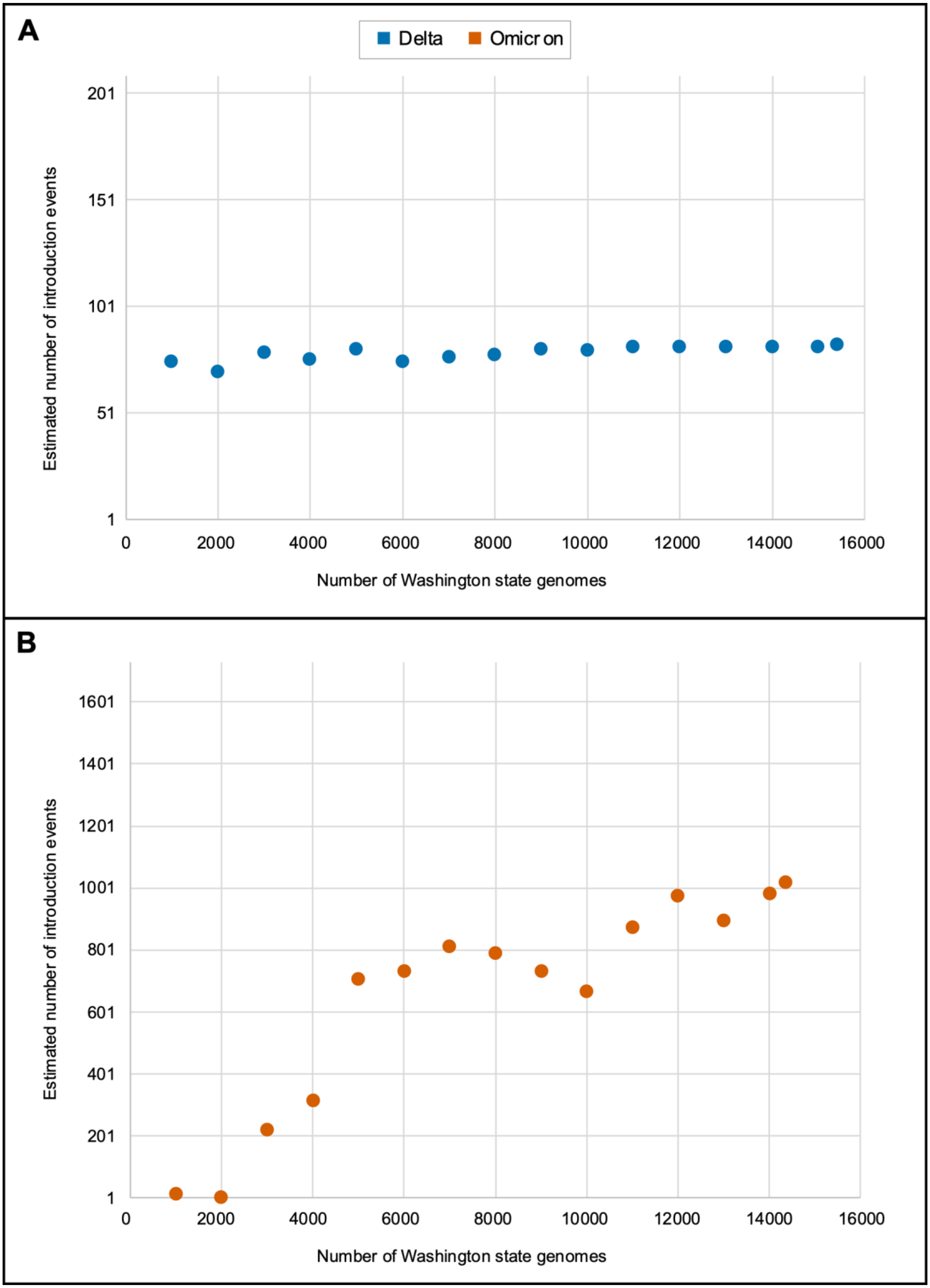
A
Delta
Omicr on
Estimated number of introduction events
201
151
101
51
1
0
2000
4000
6000
8000
10000
12000
14000
16000
Number of Washington state genomes
B
Estimated number of introduction events
1601
1401
1201
1001
801
601
401
201
1
0
2000
4000
6000
8000
10000
12000
14000
16000
Number of Washington state genomes

Supplemental Figure 2. **Accuracy analysis of number of Delta (A) and Omicron (B) introduction events onto campus represented by sequenced genomes.** Sample size of the pool of non-study Washington state genomes used in each analysis is on the x-axis and the resulting estimate for the number of introduction event of each variant onto campus for each analysis is on the y-axis. In (A), the vertical axis varies from 1 to 209, the full range of possible values for the introduction number estimate for this variant. Similarly, in (B), the vertical axis varies from 1 to 1,730.

Regression Analysis R Code

```
# Husky Coronavirus Testing Delta/ Omicron Manuscript
# Multiple linear regression of CT value on omicron vs. delta

# load libraries
library(tidyverse)
library(RColorBrewer)
library(visreg)

# regression

  #unadjusted regressions

  # ct value on variant type
model.unadj <- lm(avg_orf1b ~ who_label, data = dat)
summary(model.unadj)
model.unadj.results <- cbind(coefficient = coef(model.unadj), confint(model.unadj), p
= summary(model.unadj)$coefficients[,4] )
model.unadj.results <- as.data.frame(model.unadj.results)

  # ct value on age
model.unadj2 <- lm(avg_orf1b ~ age, data = dat)
summary(model.unadj2)
model.unadj.results2 <- cbind(coefficient = coef(model.unadj2), confint(model.unadj2),
p = summary(model.unadj2)$coefficients[,4] )
model.unadj.results2 <- as.data.frame(model.unadj.results2)

  # ct value on sx status
model.unadj3 <- lm(avg_orf1b ~ sx, data = dat)
summary(model.unadj3)
model.unadj.results3 <- cbind(coefficient = coef(model.unadj3), confint(model.unadj3),
p = summary(model.unadj3)$coefficients[,4] )
model.unadj.results3 <- as.data.frame(model.unadj.results3)

# ct value on avg RNase P gene
model.unadj4 <- lm(avg_orf1b ~ avg_rnase_p, data = dat)
summary(model.unadj4)
model.unadj.results4 <- cbind(coefficient = coef(model.unadj4), confint(model.unadj4),
p = summary(model.unadj4)$coefficients[,4] )
model.unadj.results4 <- as.data.frame(model.unadj.results4)

  # all omicron and delta cases
model.adj <- lm(avg_orf1b ~ who_label + age + avg_rnase_p, data = dat)

summary(model.adj)
model.adj.results <- cbind(coefficient = coef(model.adj), confint(model.adj), p =
summary(model.adj)$coefficients[,4] )
model.adj.results <- as.data.frame(model.adj.results)

# symptomatic omicron and delta cases only - adjusted for days since sx onset
model.adj.sx <- lm(avg_orf1b ~ who_label + age + symptoms_days + avg_rnase_p, data =
dat_sx)

summary(model.adj.sx)
model.adj.sx.results <- cbind(coefficient = coef(model.adj.sx), confint(model.adj.sx),
p = summary(model.adj.sx)$coefficients[,4] )
model.adj.sx.results <- as.data.frame(model.adj.sx.results)

# ct value on variant - symptomatic cases only
model.unadj5 <- lm(avg_orf1b ~ who_label, data = dat_sx)
summary(model.unadj5)
```

```
model.unadj.results5 <- cbind(coefficient = coef(model.unadj5), confint(model.unadj5),
p = summary(model.unadj5)$coefficients[,4] )
model.unadj.results5 <- as.data.frame(model.unadj.results5)

# ct value on age - symptomatic cases only
model.unadj6 <- lm(avg_orf1b ~ age, data = dat_sx)
summary(model.unadj6)
model.unadj.results6 <- cbind(coefficient = coef(model.unadj6), confint(model.unadj6),
p = summary(model.unadj6)$coefficients[,4] )
model.unadj.results6 <- as.data.frame(model.unadj.results6)

# ct value on days since sx onset - symptomatic cases only
model.unadj7 <- lm(avg_orf1b ~ symptoms_days, data = dat_sx)
summary(model.unadj7)
model.unadj.results7 <- cbind(coefficient = coef(model.unadj7), confint(model.unadj7),
p = summary(model.unadj7)$coefficients[,4] )
model.unadj.results7 <- as.data.frame(model.unadj.results7)

# ct value on RNase P - symptomatic cases only
model.unadj8 <- lm(avg_orf1b ~ avg_rnase_p, data = dat_sx)
summary(model.unadj8)
model.unadj.results8 <- cbind(coefficient = coef(model.unadj8), confint(model.unadj8),
p = summary(model.unadj8)$coefficients[,4] )
model.unadj.results8 <- as.data.frame(model.unadj.results8)

# among those confirmed vaxxed - adjusted for time since vax
model.adj.vax.days <- lm(avg_orf1b ~ who_label + age + sx + avg_rnase_p + vax_status +
last_dose_days, data = )

summary(model.adj.vax.days)
model.adj.vax.days.results <- cbind(coefficient = coef(model.adj.vax.days),
confint(model.adj.vax.days), p = summary(model.adj.vax.days)$coefficients[,4] )
model.adj.vax.days.results <- as.data.frame(model.adj.vax.days.results)

# ct value on variant - among those confirmed vaxxed
model.unadj14 <- lm(avg_orf1b ~ who_label, data = dat_vax_days)
summary(model.unadj14)
model.unadj.results14 <- cbind(coefficient = coef(model.unadj14),
confint(model.unadj14), p = summary(model.unadj14)$coefficients[,4] )
model.unadj.results14 <- as.data.frame(model.unadj.results14)

# ct value on age - among those confirmed vaxxed
model.unadj15 <- lm(avg_orf1b ~ age, data = dat_vax_days)
summary(model.unadj15)
model.unadj.results15 <- cbind(coefficient = coef(model.unadj15),
confint(model.unadj15), p = summary(model.unadj15)$coefficients[,4] )
model.unadj.results15 <- as.data.frame(model.unadj.results15)

# ct value on symptom status - among those confirmed vaxxed
model.unadj16 <- lm(avg_orf1b ~ sx, data = dat_vax_days)
summary(model.unadj16)
model.unadj.results16 <- cbind(coefficient = coef(model.unadj16),
confint(model.unadj16), p = summary(model.unadj16)$coefficients[,4] )
model.unadj.results16 <- as.data.frame(model.unadj.results16)

# ct value on ct value on RNase P - among those confirmed vaxxed
model.unadj17 <- lm(avg_orf1b ~ avg_rnase_p, data = dat_vax_days)
summary(model.unadj17)
model.unadj.results17 <- cbind(coefficient = coef(model.unadj17),
confint(model.unadj17), p = summary(model.unadj17)$coefficients[,4] )
model.unadj.results17 <- as.data.frame(model.unadj.results17)

# ct value on ct value on vax status - among those confirmed vaxxed
```

```
model.unadj18 <- lm(avg_orf1b ~ vax_status, data = dat_vax_days)
summary(model.unadj18)
model.unadj.results18 <- cbind(coefficient = coef(model.unadj18),
confint(model.unadj18), p = summary(model.unadj18)$coefficients[,4] )
model.unadj.results18 <- as.data.frame(model.unadj.results18)

# ct value on ct value on days since last vax dose - among those confirmed vaxxed
model.unadj19 <- lm(avg_orf1b ~ last_dose_days, data = dat_vax_days)
summary(model.unadj19)
model.unadj.results19 <- cbind(coefficient = coef(model.unadj19),
confint(model.unadj19), p = summary(model.unadj19)$coefficients[,4] )
model.unadj.results19 <- as.data.frame(model.unadj.results19)

# all omicron cases by lineage

#unadjusted regressions
# ct value on lineage
model.unadj20 <- lm(avg_orf1b ~ pangolin_lineage, data = dat_lin)
summary(model.unadj20)
model.unadj.results20 <- cbind(coefficient = coef(model.unadj20),
confint(model.unadj20), p = summary(model.unadj20)$coefficients[,4] )
model.unadj.results20 <- as.data.frame(model.unadj.results20)

# ct value on age
model.unadj21 <- lm(avg_orf1b ~ age, data = dat_lin)
summary(model.unadj21)
model.unadj.results21 <- cbind(coefficient = coef(model.unadj21),
confint(model.unadj21), p = summary(model.unadj21)$coefficients[,4] )
model.unadj.results21 <- as.data.frame(model.unadj.results21)

# ct value on sx status
model.unadj22 <- lm(avg_orf1b ~ sx, data = dat_lin)
summary(model.unadj22)
model.unadj.results22 <- cbind(coefficient = coef(model.unadj22),
confint(model.unadj22), p = summary(model.unadj22)$coefficients[,4] )
model.unadj.results22 <- as.data.frame(model.unadj.results22)

# ct value on avg RNase P gene
model.unadj23 <- lm(avg_orf1b ~ avg_rnase_p, data = dat_lin)
summary(model.unadj23)
model.unadj.results23 <- cbind(coefficient = coef(model.unadj23),
confint(model.unadj23), p = summary(model.unadj23)$coefficients[,4] )
model.unadj.results23 <- as.data.frame(model.unadj.results23)

# adjusted
model.adj.lin <- lm(avg_orf1b ~ pangolin_lineage + age + sx + avg_rnase_p, data =
dat_lin)

summary(model.adj.lin)
model.adj.results.lin <- cbind(coefficient = coef(model.adj.lin),
confint(model.adj.lin), p = summary(model.adj.lin)$coefficients[,4] )
model.adj.results.lin <- as.data.frame(model.adj.results)
```

GenBank Accession Numbers:

Virus_Name	Variant	EPI_ISL_ID	Genbank_ID
hCoV-19/USA/WA-S12563/2021	Delta	EPI_ISL_5329370	OK546612
hCoV-19/USA/WA-S12564/2021	Delta	EPI_ISL_5329277	OK546613
hCoV-19/USA/WA-S12568/2021	Delta	EPI_ISL_5329155	OK546617
hCoV-19/USA/WA-S12567/2021	Delta	EPI_ISL_5329154	OK546616
hCoV-19/USA/WA-S12566/2021	Delta	EPI_ISL_5329332	OK546615
hCoV-19/USA/WA-S12569/2021	Delta	EPI_ISL_5329372	OK546618
hCoV-19/USA/WA-S12570/2021	Delta	EPI_ISL_5329208	OK546619
hCoV-19/USA/WA-S12572/2021	Delta	EPI_ISL_5329156	OK546621
hCoV-19/USA/WA-S12867/2021	Delta	EPI_ISL_5629257	OP208794
hCoV-19/USA/WA-S12573/2021	Delta	EPI_ISL_5329157	OK546622
hCoV-19/USA/WA-S11617/2021	Delta	EPI_ISL_4636331	OK335515
hCoV-19/USA/WA-S12576/2021	Delta	EPI_ISL_5329425	OK546625
hCoV-19/USA/WA-S12577/2021	Delta	EPI_ISL_5329158	OK546626
hCoV-19/USA/WA-S12575/2021	Delta	EPI_ISL_5329352	OK546624
hCoV-19/USA/WA-S12574/2021	Delta	EPI_ISL_5329301	OK546623
hCoV-19/USA/WA-S12580/2021	Delta	EPI_ISL_5329419	OK546629
hCoV-19/USA/WA-S12578/2021	Delta	EPI_ISL_5329251	OK546627
hCoV-19/USA/WA-S12579/2021	Delta	EPI_ISL_5329326	OK546628
hCoV-19/USA/WA-S12581/2021	Delta	EPI_ISL_5329426	OK546630
hCoV-19/USA/WA-S12899/2021	Delta	EPI_ISL_5629289	OP208795
hCoV-19/USA/WA-S12030/2021	Delta	EPI_ISL_4873599	OK374750
hCoV-19/USA/WA-S12034/2021	Delta	EPI_ISL_4874250	OK374752
hCoV-19/USA/WA-S12033/2021	Delta	EPI_ISL_4873513	OK374751
hCoV-19/USA/WA-S12037/2021	Delta	EPI_ISL_4873530	OK374754
hCoV-19/USA/WA-S12582/2021	Delta	EPI_ISL_5329159	OK546631
hCoV-19/USA/WA-S12035/2021	Delta	EPI_ISL_4873523	OK374753
hCoV-19/USA/WA-S12038/2021	Delta	EPI_ISL_4873531	OK374755
hCoV-19/USA/WA-S12586/2021	Delta	EPI_ISL_5329428	OK546633
hCoV-19/USA/WA-S12585/2021	Delta	EPI_ISL_5329160	OK546632
hCoV-19/USA/WA-S12591/2021	Delta	EPI_ISL_5329293	OK546634
hCoV-19/USA/WA-S12602/2021	Delta	EPI_ISL_5329165	OK546645
hCoV-19/USA/WA-S12594/2021	Delta	EPI_ISL_5329350	OK546637
hCoV-19/USA/WA-S12600/2021	Delta	EPI_ISL_5329164	OK546643
hCoV-19/USA/WA-S12592/2021	Delta	EPI_ISL_5329415	OK546635
hCoV-19/USA/WA-S12601/2021	Delta	EPI_ISL_5329334	OK546644
hCoV-19/USA/WA-S12595/2021	Delta	EPI_ISL_5329161	OK546638
hCoV-19/USA/WA-S12599/2021	Delta	EPI_ISL_5329259	OK546642
hCoV-19/USA/WA-S12596/2021	Delta	EPI_ISL_5329162	OK546639
hCoV-19/USA/WA-S12597/2021	Delta	EPI_ISL_5329163	OK546640
hCoV-19/USA/WA-S12593/2021	Delta	EPI_ISL_5329399	OK546636
hCoV-19/USA/WA-S12598/2021	Delta	EPI_ISL_5329276	OK546641
hCoV-19/USA/WA-S12610/2021	Delta	EPI_ISL_5329168	OK546652
hCoV-19/USA/WA-S12606/2021	Delta	EPI_ISL_5329166	OK546648
hCoV-19/USA/WA-S12616/2021	Delta	EPI_ISL_5329169	OK546658
hCoV-19/USA/WA-S12934/2021	Delta	EPI_ISL_5629324	OP208796
hCoV-19/USA/WA-S12607/2021	Delta	EPI_ISL_5329357	OK546649
hCoV-19/USA/WA-S12611/2021	Delta	EPI_ISL_5329311	OK546653
hCoV-19/USA/WA-S12612/2021	Delta	EPI_ISL_5329424	OK546654
hCoV-19/USA/WA-S12603/2021	Delta	EPI_ISL_5329078	OK546646

hCoV-19/USA/WA-S12608/2021	Delta	EPI_ISL_5329167	OK546650
hCoV-19/USA/WA-S12615/2021	Delta	EPI_ISL_5329185	OK546657
hCoV-19/USA/WA-S12609/2021	Delta	EPI_ISL_5329421	OK546651
hCoV-19/USA/WA-S12933/2021	Delta	EPI_ISL_5629323	OP208797
hCoV-19/USA/WA-S12613/2021	Delta	EPI_ISL_5329264	OK546655
hCoV-19/USA/WA-S13175/2021	Delta	EPI_ISL_5903196	OL333786
hCoV-19/USA/WA-S12935/2021	Delta	EPI_ISL_5629325	OP208798
hCoV-19/USA/WA-S12614/2021	Delta	EPI_ISL_5329368	OK546656
hCoV-19/USA/WA-S12633/2021	Delta	EPI_ISL_5329174	OK546675
hCoV-19/USA/WA-S12628/2021	Delta	EPI_ISL_5329173	OK546670
hCoV-19/USA/WA-S12618/2021	Delta	EPI_ISL_5329340	OK546660
hCoV-19/USA/WA-S12624/2021	Delta	EPI_ISL_5329172	OK546666
hCoV-19/USA/WA-S12621/2021	Delta	EPI_ISL_5329353	OK546663
hCoV-19/USA/WA-S12632/2021	Delta	EPI_ISL_5329316	OK546674
hCoV-19/USA/WA-S12627/2021	Delta	EPI_ISL_5329342	OK546669
hCoV-19/USA/WA-S12617/2021	Delta	EPI_ISL_5329170	OK546659
hCoV-19/USA/WA-S12625/2021	Delta	EPI_ISL_5329257	OK546667
hCoV-19/USA/WA-S15538/2021	Delta	EPI_ISL_8035049	OM003858
hCoV-19/USA/WA-S12629/2021	Delta	EPI_ISL_5329343	OK546671
hCoV-19/USA/WA-S15539/2021	Delta	EPI_ISL_8035050	OP209801
hCoV-19/USA/WA-S12620/2021	Delta	EPI_ISL_5329365	OK546662
hCoV-19/USA/WA-S12630/2021	Delta	EPI_ISL_5329389	OK546672
hCoV-19/USA/WA-S12626/2021	Delta	EPI_ISL_5329397	OK546668
hCoV-19/USA/WA-S12623/2021	Delta	EPI_ISL_5329341	OK546665
hCoV-19/USA/WA-S15537/2021	Delta	EPI_ISL_8035048	OM003857
hCoV-19/USA/WA-S12622/2021	Delta	EPI_ISL_5329345	OK546664
hCoV-19/USA/WA-S12631/2021	Delta	EPI_ISL_5329378	OK546673
hCoV-19/USA/WA-S15534/2021	Delta	EPI_ISL_8035045	OM003854
hCoV-19/USA/WA-S12643/2021	Delta	EPI_ISL_5329182	OK546685
hCoV-19/USA/WA-S12645/2021	Delta	EPI_ISL_5329363	OK546687
hCoV-19/USA/WA-S12636/2021	Delta	EPI_ISL_5329297	OK546678
hCoV-19/USA/WA-S15536/2021	Delta	EPI_ISL_8035047	OM003856
hCoV-19/USA/WA-S12634/2021	Delta	EPI_ISL_5329175	OK546676
hCoV-19/USA/WA-S12639/2021	Delta	EPI_ISL_5329366	OK546681
hCoV-19/USA/WA-S15535/2021	Delta	EPI_ISL_8035046	OM003855
hCoV-19/USA/WA-S12638/2021	Delta	EPI_ISL_5329377	OK546680
hCoV-19/USA/WA-S12640/2021	Delta	EPI_ISL_5329298	OK546682
hCoV-19/USA/WA-S12635/2021	Delta	EPI_ISL_5329176	OK546677
hCoV-19/USA/WA-S15533/2021	Delta	EPI_ISL_8035044	OM003853
hCoV-19/USA/WA-S12646/2021	Delta	EPI_ISL_5329302	OK546688
hCoV-19/USA/WA-S12644/2021	Delta	EPI_ISL_5329080	OK546686
hCoV-19/USA/WA-S12642/2021	Delta	EPI_ISL_5329181	OK546684
hCoV-19/USA/WA-S15532/2021	Delta	EPI_ISL_8035043	OM003852
hCoV-19/USA/WA-S12641/2021	Delta	EPI_ISL_5329079	OK546683
hCoV-19/USA/WA-S12637/2021	Delta	EPI_ISL_5329364	OK546679
hCoV-19/USA/WA-S15540/2021	Delta	EPI_ISL_8035051	OM003859
hCoV-19/USA/WA-S15545/2021	Delta	EPI_ISL_8035056	OM003864
hCoV-19/USA/WA-S15551/2021	Delta	EPI_ISL_8035062	OM003870
hCoV-19/USA/WA-S15558/2021	Delta	EPI_ISL_8035069	OM003876
hCoV-19/USA/WA-S15556/2021	Delta	EPI_ISL_8035067	OM003874
hCoV-19/USA/WA-S15546/2021	Delta	EPI_ISL_8035057	OM003865

hCoV-19/USA/WA-S15548/2021	Delta	EPI_ISL_8035059	OM003867
hCoV-19/USA/WA-S15549/2021	Delta	EPI_ISL_8035060	OM003868
hCoV-19/USA/WA-S15552/2021	Delta	EPI_ISL_8035063	OM003871
hCoV-19/USA/WA-S15550/2021	Delta	EPI_ISL_8035061	OM003869
hCoV-19/USA/WA-S15543/2021	Delta	EPI_ISL_8035054	OM003862
hCoV-19/USA/WA-S15541/2021	Delta	EPI_ISL_8035052	OM003860
hCoV-19/USA/WA-S15547/2021	Delta	EPI_ISL_8035058	OM003866
hCoV-19/USA/WA-S15555/2021	Delta	EPI_ISL_8035066	OM003873
hCoV-19/USA/WA-S15553/2021	Delta	EPI_ISL_8035064	OM003872
hCoV-19/USA/WA-S15557/2021	Delta	EPI_ISL_8035068	OM003875
hCoV-19/USA/WA-S15559/2021	Delta	EPI_ISL_8035070	OM003877
hCoV-19/USA/WA-S15569/2021	Delta	EPI_ISL_8035080	OM003885
hCoV-19/USA/WA-S15562/2021	Delta	EPI_ISL_8035073	OM003878
hCoV-19/USA/WA-S15564/2021	Delta	EPI_ISL_8035075	OM003880
hCoV-19/USA/WA-S13183/2021	Delta	EPI_ISL_5903204	OL333789
hCoV-19/USA/WA-S15568/2021	Delta	EPI_ISL_8035079	OM003884
hCoV-19/USA/WA-S15574/2021	Delta	EPI_ISL_8035085	OM003890
hCoV-19/USA/WA-S15566/2021	Delta	EPI_ISL_8035077	OM003882
hCoV-19/USA/WA-S15573/2021	Delta	EPI_ISL_8035084	OM003889
hCoV-19/USA/WA-S15567/2021	Delta	EPI_ISL_8035078	OM003883
hCoV-19/USA/WA-S15563/2021	Delta	EPI_ISL_8035074	OM003879
hCoV-19/USA/WA-S15565/2021	Delta	EPI_ISL_8035076	OM003881
hCoV-19/USA/WA-S15572/2021	Delta	EPI_ISL_8035083	OM003888
hCoV-19/USA/WA-S15571/2021	Delta	EPI_ISL_8035082	OM003887
hCoV-19/USA/WA-S15576/2021	Delta	EPI_ISL_8035087	OM003892
hCoV-19/USA/WA-S15582/2021	Delta	EPI_ISL_8035093	OM003898
hCoV-19/USA/WA-S15575/2021	Delta	EPI_ISL_8035086	OM003891
hCoV-19/USA/WA-S15570/2021	Delta	EPI_ISL_8035081	OM003886
hCoV-19/USA/WA-S15583/2021	Delta	EPI_ISL_8035094	OM003899
hCoV-19/USA/WA-S15587/2021	Delta	EPI_ISL_8035098	OM003902
hCoV-19/USA/WA-S15577/2021	Delta	EPI_ISL_8035088	OM003893
hCoV-19/USA/WA-S15578/2021	Delta	EPI_ISL_8035089	OM003894
hCoV-19/USA/WA-S15586/2021	Delta	EPI_ISL_8035097	OM003901
hCoV-19/USA/WA-S15580/2021	Delta	EPI_ISL_8035091	OM003896
hCoV-19/USA/WA-S15588/2021	Delta	EPI_ISL_8035099	OM003903
hCoV-19/USA/WA-S15585/2021	Delta	EPI_ISL_8035096	OP209802
hCoV-19/USA/WA-S15581/2021	Delta	EPI_ISL_8035092	OM003897
hCoV-19/USA/WA-S15584/2021	Delta	EPI_ISL_8035095	OM003900
hCoV-19/USA/WA-S15598/2021	Delta	EPI_ISL_8035109	OM003913
hCoV-19/USA/WA-S15589/2021	Delta	EPI_ISL_8035100	OM003904
hCoV-19/USA/WA-S14849/2021	Delta	EPI_ISL_7285363	OL740674
hCoV-19/USA/WA-S15596/2021	Delta	EPI_ISL_8035107	OM003911
hCoV-19/USA/WA-S15597/2021	Delta	EPI_ISL_8035108	OM003912
hCoV-19/USA/WA-S15591/2021	Delta	EPI_ISL_8035102	OM003906
hCoV-19/USA/WA-S15594/2021	Delta	EPI_ISL_8035105	OM003909
hCoV-19/USA/WA-S15590/2021	Delta	EPI_ISL_8035101	OM003905
hCoV-19/USA/WA-S15595/2021	Delta	EPI_ISL_8035106	OM003910
hCoV-19/USA/WA-S15599/2021	Delta	EPI_ISL_8035110	OM003914
hCoV-19/USA/WA-S14847/2021	Delta	EPI_ISL_7285408	OL740672
hCoV-19/USA/WA-S14846/2021	Delta	EPI_ISL_7285407	OL740671
hCoV-19/USA/WA-S14852/2021	Delta	EPI_ISL_7285409	OL740677

hCoV-19/USA/WA-S14853/2021	Delta	EPI_ISL_7285337	OL740678
hCoV-19/USA/WA-S13207/2021	Delta	EPI_ISL_5903228	OL333793
hCoV-19/USA/WA-S13206/2021	Delta	EPI_ISL_5903227	OL333792
hCoV-19/USA/WA-S14850/2021	Delta	EPI_ISL_7285336	OL740675
hCoV-19/USA/WA-S13205/2021	Delta	EPI_ISL_5903226	OL333791
hCoV-19/USA/WA-S13204/2021	Delta	EPI_ISL_5903225	OL333790
hCoV-19/USA/WA-S13217/2021	Delta	EPI_ISL_5903238	OL333795
hCoV-19/USA/WA-S13218/2021	Delta	EPI_ISL_5903239	OL333796
hCoV-19/USA/WA-S13219/2021	Delta	EPI_ISL_5903240	OL333797
hCoV-19/USA/WA-S13221/2021	Delta	EPI_ISL_5903242	OL333798
hCoV-19/USA/WA-S13222/2021	Delta	EPI_ISL_5903243	OL333799
hCoV-19/USA/WA-S13223/2021	Delta	EPI_ISL_5903244	OL333800
hCoV-19/USA/WA-S13228/2021	Delta	EPI_ISL_5903249	OP208799
hCoV-19/USA/WA-S13226/2021	Delta	EPI_ISL_5903247	OL333803
hCoV-19/USA/WA-S13225/2021	Delta	EPI_ISL_5903246	OL333802
hCoV-19/USA/WA-S13232/2021	Delta	EPI_ISL_5903253	OL333804
hCoV-19/USA/WA-S13233/2021	Delta	EPI_ISL_5903254	OL333805
hCoV-19/USA/WA-S13235/2021	Delta	EPI_ISL_5903256	OL333807
hCoV-19/USA/WA-S13236/2021	Delta	EPI_ISL_5903257	OL333808
hCoV-19/USA/WA-S13237/2021	Delta	EPI_ISL_5903258	OL333809
hCoV-19/USA/WA-S13243/2021	Delta	EPI_ISL_5903264	OL333810
hCoV-19/USA/WA-S14862/2021	Delta	EPI_ISL_7285415	OL740679
hCoV-19/USA/WA-S15051/2021	Delta	EPI_ISL_7285343	OL740681
hCoV-19/USA/WA-S15052/2021	Delta	EPI_ISL_7285577	OL740682
hCoV-19/USA/WA-S15050/2021	Delta	EPI_ISL_7285393	OL740680
hCoV-19/USA/WA-S15345/2021	Delta	EPI_ISL_7812620	OL904009
hCoV-19/USA/WA-S15054/2021	Delta	EPI_ISL_7285353	OL740684
hCoV-19/USA/WA-S15053/2021	Delta	EPI_ISL_7285578	OL740683
hCoV-19/USA/WA-S15187/2021	Delta	EPI_ISL_7285679	OL740688
hCoV-19/USA/WA-S15603/2021	Delta	EPI_ISL_8035114	OM003916
hCoV-19/USA/WA-S15647/2021	Delta	EPI_ISL_8035156	OM003918
hCoV-19/USA/WA-S15649/2021	Delta	EPI_ISL_8035158	OM003919
hCoV-19/USA/WA-S15646/2021	Delta	EPI_ISL_8035155	OM003917
hCoV-19/USA/WA-S15651/2021	Delta	EPI_ISL_8035159	OM003920
hCoV-19/USA/WA-S15652/2021	Delta	EPI_ISL_8035160	OM003921
hCoV-19/USA/WA-S15660/2021	Delta	EPI_ISL_8035168	OM003925
hCoV-19/USA/WA-S15659/2021	Delta	EPI_ISL_8035167	OM003924
hCoV-19/USA/WA-S15663/2021	Delta	EPI_ISL_8035171	OM003928
hCoV-19/USA/WA-S15662/2021	Delta	EPI_ISL_8035170	OM003927
hCoV-19/USA/WA-S15661/2021	Delta	EPI_ISL_8035169	OM003926
hCoV-19/USA/WA-S15681/2021	Delta	EPI_ISL_8035189	OM003931
hCoV-19/USA/WA-S15680/2021	Delta	EPI_ISL_8035188	OM003930
hCoV-19/USA/WA-S16159/2021	Delta	EPI_ISL_8822478	OM296961
hCoV-19/USA/WA-S15679/2021	Delta	EPI_ISL_8035187	OM003929
hCoV-19/USA/WA-S15712/2021	Delta	EPI_ISL_8035218	OM003932
hCoV-19/USA/WA-S15718/2021	Delta	EPI_ISL_8035224	OM003934
hCoV-19/USA/WA-S15717/2021	Delta	EPI_ISL_8035223	OM003933
hCoV-19/USA/WA-S15505/2021	Delta	EPI_ISL_7812780	OL904016
hCoV-19/USA/WA-S15766/2021	Delta	EPI_ISL_8035269	OM003969
hCoV-19/USA/WA-S15792/2021	Delta	EPI_ISL_8035295	OM003988
hCoV-19/USA/WA-S15929/2021	Delta	EPI_ISL_8822243	OM296755

hCoV-19/USA/WA-S15962/2021	Delta	EPI_ISL_8822277	OM296772
hCoV-19/USA/WA-S16001/2021	Delta	EPI_ISL_8822317	OM296807
hCoV-19/USA/WA-S16268/2022	Delta	EPI_ISL_9060325	OM363899
hCoV-19/USA/WA-S16311/2022	Delta	EPI_ISL_9060367	OM363941
hCoV-19/USA/WA-S16778/2022	Delta	EPI_ISL_9271980	OM445424
hCoV-19/USA/WA-S16674/2022	Delta	EPI_ISL_9271878	OM445342
hCoV-19/USA/WA-S16970/2022	Delta	EPI_ISL_9346790	OM457908
hCoV-19/USA/WA-S15497/2021	Omicron	EPI_ISL_7812772	OL904012
hCoV-19/USA/WA-S15499/2021	Omicron	EPI_ISL_7812774	OL904013
hCoV-19/USA/WA-S15504/2021	Omicron	EPI_ISL_7812779	OL904015
hCoV-19/USA/WA-S15719/2021	Omicron	EPI_ISL_8035225	OM003935
hCoV-19/USA/WA-S15501/2021	Omicron	EPI_ISL_7812776	OL904014
hCoV-19/USA/WA-S15743/2021	Omicron	EPI_ISL_8035246	OM003951
hCoV-19/USA/WA-S15723/2021	Omicron	EPI_ISL_8035229	OM003938
hCoV-19/USA/WA-S15726/2021	Omicron	EPI_ISL_8035231	OM003940
hCoV-19/USA/WA-S15725/2021	Omicron	EPI_ISL_8035230	OM003939
hCoV-19/USA/WA-S15727/2021	Omicron	EPI_ISL_8035232	OM003941
hCoV-19/USA/WA-S15721/2021	Omicron	EPI_ISL_8035227	OM003937
hCoV-19/USA/WA-S15720/2021	Omicron	EPI_ISL_8035226	OM003936
hCoV-19/USA/WA-S15746/2021	Omicron	EPI_ISL_8035249	OM003954
hCoV-19/USA/WA-S15757/2021	Omicron	EPI_ISL_8035260	OM003961
hCoV-19/USA/WA-S15759/2021	Omicron	EPI_ISL_8035262	OM003963
hCoV-19/USA/WA-S15756/2021	Omicron	EPI_ISL_8035259	OM003960
hCoV-19/USA/WA-S15748/2021	Omicron	EPI_ISL_8035251	OM003956
hCoV-19/USA/WA-S15745/2021	Omicron	EPI_ISL_8035248	OM003953
hCoV-19/USA/WA-S15755/2021	Omicron	EPI_ISL_8035258	OM003959
hCoV-19/USA/WA-S15758/2021	Omicron	EPI_ISL_8035261	OM003962
hCoV-19/USA/WA-S15747/2021	Omicron	EPI_ISL_8035250	OM003955
hCoV-19/USA/WA-S15744/2021	Omicron	EPI_ISL_8035247	OM003952
hCoV-19/USA/WA-S15749/2021	Omicron	EPI_ISL_8035252	OM003957
hCoV-19/USA/WA-S15750/2021	Omicron	EPI_ISL_8035253	OM003958
hCoV-19/USA/WA-S15760/2021	Omicron	EPI_ISL_8035263	OM003964
hCoV-19/USA/WA-S15764/2021	Omicron	EPI_ISL_8035267	OM003967
hCoV-19/USA/WA-S15765/2021	Omicron	EPI_ISL_8035268	OM003968
hCoV-19/USA/WA-S15767/2021	Omicron	EPI_ISL_8035270	OM003970
hCoV-19/USA/WA-S15768/2021	Omicron	EPI_ISL_8035271	OM003971
hCoV-19/USA/WA-S15762/2021	Omicron	EPI_ISL_8035265	OM003966
hCoV-19/USA/WA-S15769/2021	Omicron	EPI_ISL_8035272	OM003972
hCoV-19/USA/WA-S15777/2021	Omicron	EPI_ISL_8035280	OM003978
hCoV-19/USA/WA-S15776/2021	Omicron	EPI_ISL_8035279	OM003977
hCoV-19/USA/WA-S15772/2021	Omicron	EPI_ISL_8035275	OM003973
hCoV-19/USA/WA-S15778/2021	Omicron	EPI_ISL_8035281	OM003979
hCoV-19/USA/WA-S15774/2021	Omicron	EPI_ISL_8035277	OM003975
hCoV-19/USA/WA-S15775/2021	Omicron	EPI_ISL_8035278	OM003976
hCoV-19/USA/WA-S15788/2021	Omicron	EPI_ISL_8035291	OM003984
hCoV-19/USA/WA-S15787/2021	Omicron	EPI_ISL_8035290	OM003983
hCoV-19/USA/WA-S15786/2021	Omicron	EPI_ISL_8035289	OM003982
hCoV-19/USA/WA-S15791/2021	Omicron	EPI_ISL_8035294	OM003987
hCoV-19/USA/WA-S15795/2021	Omicron	EPI_ISL_8035298	OM003991
hCoV-19/USA/WA-S15793/2021	Omicron	EPI_ISL_8035296	OM003989
hCoV-19/USA/WA-S15789/2021	Omicron	EPI_ISL_8035292	OM003985

hCoV-19/USA/WA-S15796/2021	Omicron	EPI_ISL_8035299	OM003992
hCoV-19/USA/WA-S15790/2021	Omicron	EPI_ISL_8035293	OM003986
hCoV-19/USA/WA-S15794/2021	Omicron	EPI_ISL_8035297	OM003990
hCoV-19/USA/WA-S15825/2021	Omicron	EPI_ISL_8035327	OM003998
hCoV-19/USA/WA-S15827/2021	Omicron	EPI_ISL_8035329	OM004000
hCoV-19/USA/WA-S15824/2021	Omicron	EPI_ISL_8035326	OM003997
hCoV-19/USA/WA-S16164/2021	Omicron	EPI_ISL_8822483	OM296964
hCoV-19/USA/WA-S15823/2021	Omicron	EPI_ISL_8035325	OM003996
hCoV-19/USA/WA-S16171/2021	Omicron	EPI_ISL_8822491	OM296967
hCoV-19/USA/WA-S16169/2021	Omicron	EPI_ISL_8822489	OM296965
hCoV-19/USA/WA-S16173/2021	Omicron	EPI_ISL_8822493	OM296969
hCoV-19/USA/WA-S16170/2021	Omicron	EPI_ISL_8822490	OM296966
hCoV-19/USA/WA-S16172/2021	Omicron	EPI_ISL_8822492	OM296968
hCoV-19/USA/WA-S16177/2021	Omicron	EPI_ISL_8822497	OM296973
hCoV-19/USA/WA-S16184/2021	Omicron	EPI_ISL_8822504	OM296974
hCoV-19/USA/WA-S16175/2021	Omicron	EPI_ISL_8822495	OM296971
hCoV-19/USA/WA-S16176/2021	Omicron	EPI_ISL_8822496	OM296972
hCoV-19/USA/WA-S16174/2021	Omicron	EPI_ISL_8822494	OM296970
hCoV-19/USA/WA-S15842/2021	Omicron	EPI_ISL_8822154	OM296707
hCoV-19/USA/WA-S15829/2021	Omicron	EPI_ISL_8822141	OM296701
hCoV-19/USA/WA-S15833/2021	Omicron	EPI_ISL_8822145	OM296705
hCoV-19/USA/WA-S16186/2021	Omicron	EPI_ISL_8822506	OM296976
hCoV-19/USA/WA-S15831/2021	Omicron	EPI_ISL_8822143	OM296703
hCoV-19/USA/WA-S16185/2021	Omicron	EPI_ISL_8822505	OM296975
hCoV-19/USA/WA-S16189/2021	Omicron	EPI_ISL_8822509	OM296979
hCoV-19/USA/WA-S15828/2021	Omicron	EPI_ISL_8822140	OM296700
hCoV-19/USA/WA-S15841/2021	Omicron	EPI_ISL_8822153	OM296706
hCoV-19/USA/WA-S16187/2021	Omicron	EPI_ISL_8822507	OM296977
hCoV-19/USA/WA-S15832/2021	Omicron	EPI_ISL_8822144	OM296704
hCoV-19/USA/WA-S15830/2021	Omicron	EPI_ISL_8822142	OM296702
hCoV-19/USA/WA-S16188/2021	Omicron	EPI_ISL_8822508	OM296978
hCoV-19/USA/WA-S15843/2021	Omicron	EPI_ISL_8822155	OM296708
hCoV-19/USA/WA-S15863/2021	Omicron	EPI_ISL_8822176	OM296720
hCoV-19/USA/WA-S15864/2021	Omicron	EPI_ISL_8822177	OM296721
hCoV-19/USA/WA-S15846/2021	Omicron	EPI_ISL_8822158	OM296711
hCoV-19/USA/WA-S15850/2021	Omicron	EPI_ISL_8822163	OM296715
hCoV-19/USA/WA-S15845/2021	Omicron	EPI_ISL_8822157	OM296710
hCoV-19/USA/WA-S15847/2021	Omicron	EPI_ISL_8822160	OM296712
hCoV-19/USA/WA-S15860/2021	Omicron	EPI_ISL_8822173	OM296717
hCoV-19/USA/WA-S15862/2021	Omicron	EPI_ISL_8822175	OM296719
hCoV-19/USA/WA-S15866/2021	Omicron	EPI_ISL_8822179	OM296723
hCoV-19/USA/WA-S15865/2021	Omicron	EPI_ISL_8822178	OM296722
hCoV-19/USA/WA-S15844/2021	Omicron	EPI_ISL_8822156	OM296709
hCoV-19/USA/WA-S15861/2021	Omicron	EPI_ISL_8822174	OM296718
hCoV-19/USA/WA-S15851/2021	Omicron	EPI_ISL_8822164	OM296716
hCoV-19/USA/WA-S15848/2021	Omicron	EPI_ISL_8822161	OM296713
hCoV-19/USA/WA-S15881/2021	Omicron	EPI_ISL_8822195	OM296738
hCoV-19/USA/WA-S15920/2021	Omicron	EPI_ISL_8822234	OM296746
hCoV-19/USA/WA-S15882/2021	Omicron	EPI_ISL_8822196	OM296739
hCoV-19/USA/WA-S15872/2021	Omicron	EPI_ISL_8822185	OM296729
hCoV-19/USA/WA-S15875/2021	Omicron	EPI_ISL_8822188	OM296732

hCoV-19/USA/WA-S15880/2021	Omicron	EPI_ISL_8822194	OM296737
hCoV-19/USA/WA-S15867/2021	Omicron	EPI_ISL_8822180	OM296724
hCoV-19/USA/WA-S15869/2021	Omicron	EPI_ISL_8822182	OM296726
hCoV-19/USA/WA-S15926/2021	Omicron	EPI_ISL_8822240	OM296752
hCoV-19/USA/WA-S15868/2021	Omicron	EPI_ISL_8822181	OM296725
hCoV-19/USA/WA-S15878/2021	Omicron	EPI_ISL_8822192	OM296735
hCoV-19/USA/WA-S15871/2021	Omicron	EPI_ISL_8822184	OM296728
hCoV-19/USA/WA-S15870/2021	Omicron	EPI_ISL_8822183	OM296727
hCoV-19/USA/WA-S15879/2021	Omicron	EPI_ISL_8822193	OM296736
hCoV-19/USA/WA-S15873/2021	Omicron	EPI_ISL_8822186	OM296730
hCoV-19/USA/WA-S15877/2021	Omicron	EPI_ISL_8822190	OM296734
hCoV-19/USA/WA-S15874/2021	Omicron	EPI_ISL_8822187	OM296731
hCoV-19/USA/WA-S15925/2021	Omicron	EPI_ISL_8822239	OM296751
hCoV-19/USA/WA-S15966/2021	Omicron	EPI_ISL_8822281	OM296776
hCoV-19/USA/WA-S16202/2021	Omicron	EPI_ISL_9060259	OM363884
hCoV-19/USA/WA-S15951/2021	Omicron	EPI_ISL_8822266	OM296761
hCoV-19/USA/WA-S15927/2021	Omicron	EPI_ISL_8822241	OM296753
hCoV-19/USA/WA-S15960/2021	Omicron	EPI_ISL_8822275	OM296770
hCoV-19/USA/WA-S15922/2021	Omicron	EPI_ISL_8822236	OM296748
hCoV-19/USA/WA-S15933/2021	Omicron	EPI_ISL_8822247	OM296759
hCoV-19/USA/WA-S15959/2021	Omicron	EPI_ISL_8822274	OM296769
hCoV-19/USA/WA-S15923/2021	Omicron	EPI_ISL_8822237	OM296749
hCoV-19/USA/WA-S15928/2021	Omicron	EPI_ISL_8822242	OM296754
hCoV-19/USA/WA-S15955/2021	Omicron	EPI_ISL_8822270	OM296765
hCoV-19/USA/WA-S15954/2021	Omicron	EPI_ISL_8822269	OM296764
hCoV-19/USA/WA-S15934/2021	Omicron	EPI_ISL_8822248	OM296760
hCoV-19/USA/WA-S15958/2021	Omicron	EPI_ISL_8822273	OM296768
hCoV-19/USA/WA-S15930/2021	Omicron	EPI_ISL_8822244	OM296756
hCoV-19/USA/WA-S15961/2021	Omicron	EPI_ISL_8822276	OM296771
hCoV-19/USA/WA-S15956/2021	Omicron	EPI_ISL_8822271	OM296766
hCoV-19/USA/WA-S15932/2021	Omicron	EPI_ISL_8822246	OM296758
hCoV-19/USA/WA-S15924/2021	Omicron	EPI_ISL_8822238	OM296750
hCoV-19/USA/WA-S15952/2021	Omicron	EPI_ISL_8822267	OM296762
hCoV-19/USA/WA-S15953/2021	Omicron	EPI_ISL_8822268	OM296763
hCoV-19/USA/WA-S15921/2021	Omicron	EPI_ISL_8822235	OM296747
hCoV-19/USA/WA-S15963/2021	Omicron	EPI_ISL_8822278	OM296773
hCoV-19/USA/WA-S15957/2021	Omicron	EPI_ISL_8822272	OM296767
hCoV-19/USA/WA-S16203/2021	Omicron	EPI_ISL_9060260	OM363885
hCoV-19/USA/WA-S16204/2021	Omicron	EPI_ISL_9060261	OM363886
hCoV-19/USA/WA-S15964/2021	Omicron	EPI_ISL_8822279	OM296774
hCoV-19/USA/WA-S15931/2021	Omicron	EPI_ISL_8822245	OM296757
hCoV-19/USA/WA-S15965/2021	Omicron	EPI_ISL_8822280	OM296775
hCoV-19/USA/WA-S15975/2021	Omicron	EPI_ISL_8822290	OM296781
hCoV-19/USA/WA-S15991/2021	Omicron	EPI_ISL_8822306	OM296797
hCoV-19/USA/WA-S16215/2021	Omicron	EPI_ISL_9060272	OM363887
hCoV-19/USA/WA-S15993/2021	Omicron	EPI_ISL_8822309	OM296799
hCoV-19/USA/WA-S15977/2021	Omicron	EPI_ISL_8822292	OM296783
hCoV-19/USA/WA-S15972/2021	Omicron	EPI_ISL_8822287	OM296778
hCoV-19/USA/WA-S15995/2021	Omicron	EPI_ISL_8822311	OM296801
hCoV-19/USA/WA-S15997/2021	Omicron	EPI_ISL_8822313	OM296803
hCoV-19/USA/WA-S15982/2021	Omicron	EPI_ISL_8822297	OM296788

hCoV-19/USA/WA-S15984/2021	Omicron	EPI_ISL_8822299	OM296790
hCoV-19/USA/WA-S15989/2021	Omicron	EPI_ISL_8822304	OM296795
hCoV-19/USA/WA-S15983/2021	Omicron	EPI_ISL_8822298	OM296789
hCoV-19/USA/WA-S15987/2021	Omicron	EPI_ISL_8822302	OM296793
hCoV-19/USA/WA-S16002/2021	Omicron	EPI_ISL_8822318	OM296808
hCoV-19/USA/WA-S15998/2021	Omicron	EPI_ISL_8822314	OM296804
hCoV-19/USA/WA-S16223/2021	Omicron	EPI_ISL_9060280	OM363890
hCoV-19/USA/WA-S16218/2021	Omicron	EPI_ISL_9060275	OM363889
hCoV-19/USA/WA-S16000/2021	Omicron	EPI_ISL_8822316	OM296806
hCoV-19/USA/WA-S15996/2021	Omicron	EPI_ISL_8822312	OM296802
hCoV-19/USA/WA-S15974/2021	Omicron	EPI_ISL_8822289	OM296780
hCoV-19/USA/WA-S15979/2021	Omicron	EPI_ISL_8822294	OM296785
hCoV-19/USA/WA-S15973/2021	Omicron	EPI_ISL_8822288	OM296779
hCoV-19/USA/WA-S15990/2021	Omicron	EPI_ISL_8822305	OM296796
hCoV-19/USA/WA-S16216/2021	Omicron	EPI_ISL_9060273	OM363888
hCoV-19/USA/WA-S15988/2021	Omicron	EPI_ISL_8822303	OM296794
hCoV-19/USA/WA-S16012/2021	Omicron	EPI_ISL_8822328	OM296818
hCoV-19/USA/WA-S15978/2021	Omicron	EPI_ISL_8822293	OM296784
hCoV-19/USA/WA-S15992/2021	Omicron	EPI_ISL_8822307	OM296798
hCoV-19/USA/WA-S15994/2021	Omicron	EPI_ISL_8822310	OM296800
hCoV-19/USA/WA-S15986/2021	Omicron	EPI_ISL_8822301	OM296792
hCoV-19/USA/WA-S15971/2021	Omicron	EPI_ISL_8822286	OM296777
hCoV-19/USA/WA-S15981/2021	Omicron	EPI_ISL_8822296	OM296787
hCoV-19/USA/WA-S15976/2021	Omicron	EPI_ISL_8822291	OM296782
hCoV-19/USA/WA-S15980/2021	Omicron	EPI_ISL_8822295	OM296786
hCoV-19/USA/WA-S16018/2021	Omicron	EPI_ISL_8822334	OM296824
hCoV-19/USA/WA-S16003/2021	Omicron	EPI_ISL_8822319	OM296809
hCoV-19/USA/WA-S16026/2021	Omicron	EPI_ISL_8822342	OM296832
hCoV-19/USA/WA-S16015/2021	Omicron	EPI_ISL_8822331	OM296821
hCoV-19/USA/WA-S16009/2021	Omicron	EPI_ISL_8822325	OM296815
hCoV-19/USA/WA-S15999/2021	Omicron	EPI_ISL_8822315	OM296805
hCoV-19/USA/WA-S16016/2021	Omicron	EPI_ISL_8822332	OM296822
hCoV-19/USA/WA-S16030/2021	Omicron	EPI_ISL_8822346	OM296836
hCoV-19/USA/WA-S16021/2021	Omicron	EPI_ISL_8822337	OM296827
hCoV-19/USA/WA-S16029/2021	Omicron	EPI_ISL_8822345	OM296835
hCoV-19/USA/WA-S16007/2021	Omicron	EPI_ISL_8822323	OM296813
hCoV-19/USA/WA-S16011/2021	Omicron	EPI_ISL_8822327	OM296817
hCoV-19/USA/WA-S16005/2021	Omicron	EPI_ISL_8822321	OM296811
hCoV-19/USA/WA-S16023/2021	Omicron	EPI_ISL_8822339	OM296829
hCoV-19/USA/WA-S16006/2021	Omicron	EPI_ISL_8822322	OM296812
hCoV-19/USA/WA-S16025/2021	Omicron	EPI_ISL_8822341	OM296831
hCoV-19/USA/WA-S16004/2021	Omicron	EPI_ISL_8822320	OM296810
hCoV-19/USA/WA-S16014/2021	Omicron	EPI_ISL_8822330	OM296820
hCoV-19/USA/WA-S16024/2021	Omicron	EPI_ISL_8822340	OM296830
hCoV-19/USA/WA-S16019/2021	Omicron	EPI_ISL_8822335	OM296825
hCoV-19/USA/WA-S16008/2021	Omicron	EPI_ISL_8822324	OM296814
hCoV-19/USA/WA-S16020/2021	Omicron	EPI_ISL_8822336	OM296826
hCoV-19/USA/WA-S16028/2021	Omicron	EPI_ISL_8822344	OM296834
hCoV-19/USA/WA-S16010/2021	Omicron	EPI_ISL_8822326	OM296816
hCoV-19/USA/WA-S16022/2021	Omicron	EPI_ISL_8822338	OM296828
hCoV-19/USA/WA-S16031/2021	Omicron	EPI_ISL_8822347	OM296837

hCoV-19/USA/WA-S16013/2021	Omicron	EPI_ISL_8822329	OM296819
hCoV-19/USA/WA-S16027/2021	Omicron	EPI_ISL_8822343	OM296833
hCoV-19/USA/WA-S16056/2022	Omicron	EPI_ISL_8822373	OM296862
hCoV-19/USA/WA-S16062/2022	Omicron	EPI_ISL_8822379	OM296868
hCoV-19/USA/WA-S16261/2022	Omicron	EPI_ISL_9060318	OM363892
hCoV-19/USA/WA-S16054/2022	Omicron	EPI_ISL_8822371	OM296860
hCoV-19/USA/WA-S16032/2022	Omicron	EPI_ISL_8822348	OM296838
hCoV-19/USA/WA-S16061/2022	Omicron	EPI_ISL_8822378	OM296867
hCoV-19/USA/WA-S16033/2022	Omicron	EPI_ISL_8822349	OM296839
hCoV-19/USA/WA-S16049/2022	Omicron	EPI_ISL_8822366	OM296855
hCoV-19/USA/WA-S16059/2022	Omicron	EPI_ISL_8822376	OM296865
hCoV-19/USA/WA-S16042/2022	Omicron	EPI_ISL_8822359	OM296848
hCoV-19/USA/WA-S16045/2022	Omicron	EPI_ISL_8822362	OM296851
hCoV-19/USA/WA-S16043/2022	Omicron	EPI_ISL_8822360	OM296849
hCoV-19/USA/WA-S16040/2022	Omicron	EPI_ISL_8822357	OM296846
hCoV-19/USA/WA-S16038/2022	Omicron	EPI_ISL_8822355	OM296844
hCoV-19/USA/WA-S16037/2022	Omicron	EPI_ISL_8822353	OM296843
hCoV-19/USA/WA-S16053/2022	Omicron	EPI_ISL_8822370	OM296859
hCoV-19/USA/WA-S16058/2022	Omicron	EPI_ISL_8822375	OM296864
hCoV-19/USA/WA-S16044/2022	Omicron	EPI_ISL_8822361	OM296850
hCoV-19/USA/WA-S16050/2022	Omicron	EPI_ISL_8822367	OM296856
hCoV-19/USA/WA-S16052/2022	Omicron	EPI_ISL_8822369	OM296858
hCoV-19/USA/WA-S16260/2022	Omicron	EPI_ISL_9060317	OM363891
hCoV-19/USA/WA-S16055/2022	Omicron	EPI_ISL_8822372	OM296861
hCoV-19/USA/WA-S16035/2022	Omicron	EPI_ISL_8822351	OM296841
hCoV-19/USA/WA-S16041/2022	Omicron	EPI_ISL_8822358	OM296847
hCoV-19/USA/WA-S16057/2022	Omicron	EPI_ISL_8822374	OM296863
hCoV-19/USA/WA-S16036/2022	Omicron	EPI_ISL_8822352	OM296842
hCoV-19/USA/WA-S16034/2022	Omicron	EPI_ISL_8822350	OM296840
hCoV-19/USA/WA-S16039/2022	Omicron	EPI_ISL_8822356	OM296845
hCoV-19/USA/WA-S16046/2022	Omicron	EPI_ISL_8822363	OM296852
hCoV-19/USA/WA-S16051/2022	Omicron	EPI_ISL_8822368	OM296857
hCoV-19/USA/WA-S16060/2022	Omicron	EPI_ISL_8822377	OM296866
hCoV-19/USA/WA-S16047/2022	Omicron	EPI_ISL_8822364	OM296853
hCoV-19/USA/WA-S16281/2022	Omicron	EPI_ISL_9060338	OM363912
hCoV-19/USA/WA-S16291/2022	Omicron	EPI_ISL_9060347	OM363921
hCoV-19/USA/WA-S16285/2022	Omicron	EPI_ISL_9060341	OM363915
hCoV-19/USA/WA-S16275/2022	Omicron	EPI_ISL_9060332	OM363906
hCoV-19/USA/WA-S16262/2022	Omicron	EPI_ISL_9060319	OM363893
hCoV-19/USA/WA-S16298/2022	Omicron	EPI_ISL_9060354	OM363928
hCoV-19/USA/WA-S16274/2022	Omicron	EPI_ISL_9060331	OM363905
hCoV-19/USA/WA-S16273/2022	Omicron	EPI_ISL_9060330	OM363904
hCoV-19/USA/WA-S16267/2022	Omicron	EPI_ISL_9060324	OM363898
hCoV-19/USA/WA-S16299/2022	Omicron	EPI_ISL_9060355	OM363929
hCoV-19/USA/WA-S16300/2022	Omicron	EPI_ISL_9060356	OM363930
hCoV-19/USA/WA-S16286/2022	Omicron	EPI_ISL_9060342	OM363916
hCoV-19/USA/WA-S16278/2022	Omicron	EPI_ISL_9060335	OM363909
hCoV-19/USA/WA-S16265/2022	Omicron	EPI_ISL_9060322	OM363896
hCoV-19/USA/WA-S16301/2022	Omicron	EPI_ISL_9060357	OM363931
hCoV-19/USA/WA-S16306/2022	Omicron	EPI_ISL_9060362	OM363936
hCoV-19/USA/WA-S16284/2022	Omicron	EPI_ISL_9060340	OM363914

hCoV-19/USA/WA-S16297/2022	Omicron	EPI_ISL_9060353	OM363927
hCoV-19/USA/WA-S16296/2022	Omicron	EPI_ISL_9060352	OM363926
hCoV-19/USA/WA-S16293/2022	Omicron	EPI_ISL_9060349	OM363923
hCoV-19/USA/WA-S16264/2022	Omicron	EPI_ISL_9060321	OM363895
hCoV-19/USA/WA-S16307/2022	Omicron	EPI_ISL_9060363	OM363937
hCoV-19/USA/WA-S16290/2022	Omicron	EPI_ISL_9060346	OM363920
hCoV-19/USA/WA-S16289/2022	Omicron	EPI_ISL_9060345	OM363919
hCoV-19/USA/WA-S16276/2022	Omicron	EPI_ISL_9060333	OM363907
hCoV-19/USA/WA-S16272/2022	Omicron	EPI_ISL_9060329	OM363903
hCoV-19/USA/WA-S16294/2022	Omicron	EPI_ISL_9060350	OM363924
hCoV-19/USA/WA-S16288/2022	Omicron	EPI_ISL_9060344	OM363918
hCoV-19/USA/WA-S16292/2022	Omicron	EPI_ISL_9060348	OM363922
hCoV-19/USA/WA-S16269/2022	Omicron	EPI_ISL_9060326	OM363900
hCoV-19/USA/WA-S16287/2022	Omicron	EPI_ISL_9060343	OM363917
hCoV-19/USA/WA-S16282/2022	Omicron	EPI_ISL_9060339	OM363913
hCoV-19/USA/WA-S16063/2022	Omicron	EPI_ISL_8822380	OM296869
hCoV-19/USA/WA-S16263/2022	Omicron	EPI_ISL_9060320	OM363894
hCoV-19/USA/WA-S16304/2022	Omicron	EPI_ISL_9060360	OM363934
hCoV-19/USA/WA-S16303/2022	Omicron	EPI_ISL_9060359	OM363933
hCoV-19/USA/WA-S16280/2022	Omicron	EPI_ISL_9060337	OM363911
hCoV-19/USA/WA-S16271/2022	Omicron	EPI_ISL_9060328	OM363902
hCoV-19/USA/WA-S16277/2022	Omicron	EPI_ISL_9060334	OM363908
hCoV-19/USA/WA-S16279/2022	Omicron	EPI_ISL_9060336	OM363910
hCoV-19/USA/WA-S16302/2022	Omicron	EPI_ISL_9060358	OM363932
hCoV-19/USA/WA-S16349/2022	Omicron	EPI_ISL_9060405	OM363979
hCoV-19/USA/WA-S16416/2022	Omicron	EPI_ISL_9060472	OM364024
hCoV-19/USA/WA-S16324/2022	Omicron	EPI_ISL_9060380	OM363954
hCoV-19/USA/WA-S16334/2022	Omicron	EPI_ISL_9060390	OM363964
hCoV-19/USA/WA-S16354/2022	Omicron	EPI_ISL_9060410	OM363984
hCoV-19/USA/WA-S16325/2022	Omicron	EPI_ISL_9060381	OM363955
hCoV-19/USA/WA-S16392/2022	Omicron	EPI_ISL_9060448	OM364000
hCoV-19/USA/WA-S16353/2022	Omicron	EPI_ISL_9060409	OM363983
hCoV-19/USA/WA-S16313/2022	Omicron	EPI_ISL_9060369	OM363943
hCoV-19/USA/WA-S16319/2022	Omicron	EPI_ISL_9060375	OM363949
hCoV-19/USA/WA-S16351/2022	Omicron	EPI_ISL_9060407	OM363981
hCoV-19/USA/WA-S16348/2022	Omicron	EPI_ISL_9060404	OM363978
hCoV-19/USA/WA-S16389/2022	Omicron	EPI_ISL_9060445	OM363997
hCoV-19/USA/WA-S16341/2022	Omicron	EPI_ISL_9060397	OM363971
hCoV-19/USA/WA-S16399/2022	Omicron	EPI_ISL_9060455	OM364007
hCoV-19/USA/WA-S16340/2022	Omicron	EPI_ISL_9060396	OM363970
hCoV-19/USA/WA-S16330/2022	Omicron	EPI_ISL_9060386	OM363960
hCoV-19/USA/WA-S16317/2022	Omicron	EPI_ISL_9060373	OM363947
hCoV-19/USA/WA-S16310/2022	Omicron	EPI_ISL_9060366	OM363940
hCoV-19/USA/WA-S16393/2022	Omicron	EPI_ISL_9060449	OM364001
hCoV-19/USA/WA-S16326/2022	Omicron	EPI_ISL_9060382	OM363956
hCoV-19/USA/WA-S16350/2022	Omicron	EPI_ISL_9060406	OM363980
hCoV-19/USA/WA-S16322/2022	Omicron	EPI_ISL_9060378	OM363952
hCoV-19/USA/WA-S16316/2022	Omicron	EPI_ISL_9060372	OM363946
hCoV-19/USA/WA-S16338/2022	Omicron	EPI_ISL_9060394	OM363968
hCoV-19/USA/WA-S16397/2022	Omicron	EPI_ISL_9060453	OM364005
hCoV-19/USA/WA-S16414/2022	Omicron	EPI_ISL_9060470	OM364022

hCoV-19/USA/WA-S16347/2022	Omicron	EPI_ISL_9060403	OM363977
hCoV-19/USA/WA-S16345/2022	Omicron	EPI_ISL_9060401	OM363975
hCoV-19/USA/WA-S16394/2022	Omicron	EPI_ISL_9060450	OM364002
hCoV-19/USA/WA-S16355/2022	Omicron	EPI_ISL_9060411	OM363985
hCoV-19/USA/WA-S16396/2022	Omicron	EPI_ISL_9060452	OM364004
hCoV-19/USA/WA-S16329/2022	Omicron	EPI_ISL_9060385	OM363959
hCoV-19/USA/WA-S16339/2022	Omicron	EPI_ISL_9060395	OM363969
hCoV-19/USA/WA-S16413/2022	Omicron	EPI_ISL_9060469	OM364021
hCoV-19/USA/WA-S16336/2022	Omicron	EPI_ISL_9060392	OM363966
hCoV-19/USA/WA-S16343/2022	Omicron	EPI_ISL_9060399	OM363973
hCoV-19/USA/WA-S16295/2022	Omicron	EPI_ISL_9060351	OM363925
hCoV-19/USA/WA-S16415/2022	Omicron	EPI_ISL_9060471	OM364023
hCoV-19/USA/WA-S16321/2022	Omicron	EPI_ISL_9060377	OM363951
hCoV-19/USA/WA-S16390/2022	Omicron	EPI_ISL_9060446	OM363998
hCoV-19/USA/WA-S16545/2022	Omicron	EPI_ISL_9271749	OM445257
hCoV-19/USA/WA-S16418/2022	Omicron	EPI_ISL_9060474	OM364026
hCoV-19/USA/WA-S16544/2022	Omicron	EPI_ISL_9271748	OM445256
hCoV-19/USA/WA-S16305/2022	Omicron	EPI_ISL_9060361	OM363935
hCoV-19/USA/WA-S16314/2022	Omicron	EPI_ISL_9060370	OM363944
hCoV-19/USA/WA-S16356/2022	Omicron	EPI_ISL_9060412	OM363986
hCoV-19/USA/WA-S16335/2022	Omicron	EPI_ISL_9060391	OM363965
hCoV-19/USA/WA-S16381/2022	Omicron	EPI_ISL_9060437	OM363989
hCoV-19/USA/WA-S16417/2022	Omicron	EPI_ISL_9060473	OM364025
hCoV-19/USA/WA-S16395/2022	Omicron	EPI_ISL_9060451	OM364003
hCoV-19/USA/WA-S16331/2022	Omicron	EPI_ISL_9060387	OM363961
hCoV-19/USA/WA-S16332/2022	Omicron	EPI_ISL_9060388	OM363962
hCoV-19/USA/WA-S16318/2022	Omicron	EPI_ISL_9060374	OM363948
hCoV-19/USA/WA-S16400/2022	Omicron	EPI_ISL_9060456	OM364008
hCoV-19/USA/WA-S16546/2022	Omicron	EPI_ISL_9271750	OM445258
hCoV-19/USA/WA-S16344/2022	Omicron	EPI_ISL_9060400	OM363974
hCoV-19/USA/WA-S16323/2022	Omicron	EPI_ISL_9060379	OM363953
hCoV-19/USA/WA-S16342/2022	Omicron	EPI_ISL_9060398	OM363972
hCoV-19/USA/WA-S16382/2022	Omicron	EPI_ISL_9060438	OM363990
hCoV-19/USA/WA-S16346/2022	Omicron	EPI_ISL_9060402	OM363976
hCoV-19/USA/WA-S16315/2022	Omicron	EPI_ISL_9060371	OM363945
hCoV-19/USA/WA-S16328/2022	Omicron	EPI_ISL_9060384	OM363958
hCoV-19/USA/WA-S16320/2022	Omicron	EPI_ISL_9060376	OM363950
hCoV-19/USA/WA-S16337/2022	Omicron	EPI_ISL_9060393	OM363967
hCoV-19/USA/WA-S16308/2022	Omicron	EPI_ISL_9060364	OM363938
hCoV-19/USA/WA-S16327/2022	Omicron	EPI_ISL_9060383	OM363957
hCoV-19/USA/WA-S16391/2022	Omicron	EPI_ISL_9060447	OM363999
hCoV-19/USA/WA-S16606/2022	Omicron	EPI_ISL_9271810	OM445276
hCoV-19/USA/WA-S16333/2022	Omicron	EPI_ISL_9060389	OM363963
hCoV-19/USA/WA-S16398/2022	Omicron	EPI_ISL_9060454	OM364006
hCoV-19/USA/WA-S16312/2022	Omicron	EPI_ISL_9060368	OM363942
hCoV-19/USA/WA-S16309/2022	Omicron	EPI_ISL_9060365	OM363939
hCoV-19/USA/WA-S16388/2022	Omicron	EPI_ISL_9060444	OM363996
hCoV-19/USA/WA-S16352/2022	Omicron	EPI_ISL_9060408	OM363982
hCoV-19/USA/WA-S16596/2022	Omicron	EPI_ISL_9271801	OM445267
hCoV-19/USA/WA-S16562/2022	Omicron	EPI_ISL_9271766	OM445263
hCoV-19/USA/WA-S16696/2022	Omicron	EPI_ISL_9271899	OM445363

hCoV-19/USA/WA-S16598/2022	Omicron	EPI_ISL_9271803	OM445269
hCoV-19/USA/WA-S16567/2022	Omicron	EPI_ISL_9271771	OM445264
hCoV-19/USA/WA-S16386/2022	Omicron	EPI_ISL_9060442	OM363994
hCoV-19/USA/WA-S16409/2022	Omicron	EPI_ISL_9060465	OM364017
hCoV-19/USA/WA-S16421/2022	Omicron	EPI_ISL_9060477	OM364029
hCoV-19/USA/WA-S16407/2022	Omicron	EPI_ISL_9060463	OM364015
hCoV-19/USA/WA-S16486/2022	Omicron	EPI_ISL_9060542	OM364059
hCoV-19/USA/WA-S16532/2022	Omicron	EPI_ISL_9060588	OM364077
hCoV-19/USA/WA-S16405/2022	Omicron	EPI_ISL_9060461	OM364013
hCoV-19/USA/WA-S16692/2022	Omicron	EPI_ISL_9271895	OM445359
hCoV-19/USA/WA-S16422/2022	Omicron	EPI_ISL_9060478	OM364030
hCoV-19/USA/WA-S16420/2022	Omicron	EPI_ISL_9060476	OM364028
hCoV-19/USA/WA-S16490/2022	Omicron	EPI_ISL_9060546	OM364063
hCoV-19/USA/WA-S16387/2022	Omicron	EPI_ISL_9060443	OM363995
hCoV-19/USA/WA-S16380/2022	Omicron	EPI_ISL_9060436	OM363988
hCoV-19/USA/WA-S16497/2022	Omicron	EPI_ISL_9060553	OM364070
hCoV-19/USA/WA-S16707/2022	Omicron	EPI_ISL_9271910	OM445373
hCoV-19/USA/WA-S16491/2022	Omicron	EPI_ISL_9060547	OM364064
hCoV-19/USA/WA-S16410/2022	Omicron	EPI_ISL_9060466	OM364018
hCoV-19/USA/WA-S16697/2022	Omicron	EPI_ISL_9271900	OM445364
hCoV-19/USA/WA-S16404/2022	Omicron	EPI_ISL_9060460	OM364012
hCoV-19/USA/WA-S16423/2022	Omicron	EPI_ISL_9060479	OM364031
hCoV-19/USA/WA-S16502/2022	Omicron	EPI_ISL_9060558	OM364075
hCoV-19/USA/WA-S16498/2022	Omicron	EPI_ISL_9060554	OM364071
hCoV-19/USA/WA-S16782/2022	Omicron	EPI_ISL_9271984	OM445428
hCoV-19/USA/WA-S16419/2022	Omicron	EPI_ISL_9060475	OM364027
hCoV-19/USA/WA-S16384/2022	Omicron	EPI_ISL_9060440	OM363992
hCoV-19/USA/WA-S16411/2022	Omicron	EPI_ISL_9060467	OM364019
hCoV-19/USA/WA-S16775/2022	Omicron	EPI_ISL_9271977	OM445421
hCoV-19/USA/WA-S16704/2022	Omicron	EPI_ISL_9271907	OM445371
hCoV-19/USA/WA-S16503/2022	Omicron	EPI_ISL_9060559	OM364076
hCoV-19/USA/WA-S16379/2022	Omicron	EPI_ISL_9060435	OM363987
hCoV-19/USA/WA-S16408/2022	Omicron	EPI_ISL_9060464	OM364016
hCoV-19/USA/WA-S16485/2022	Omicron	EPI_ISL_9060541	OM364058
hCoV-19/USA/WA-S16712/2022	Omicron	EPI_ISL_9271915	OM445378
hCoV-19/USA/WA-S16479/2022	Omicron	EPI_ISL_9060535	OM364052
hCoV-19/USA/WA-S16605/2022	Omicron	EPI_ISL_9271809	OM445275
hCoV-19/USA/WA-S16401/2022	Omicron	EPI_ISL_9060457	OM364009
hCoV-19/USA/WA-S16698/2022	Omicron	EPI_ISL_9271901	OM445365
hCoV-19/USA/WA-S16475/2022	Omicron	EPI_ISL_9060531	OM364048
hCoV-19/USA/WA-S16690/2022	Omicron	EPI_ISL_9271893	OM445357
hCoV-19/USA/WA-S16599/2022	Omicron	EPI_ISL_9271804	OM445270
hCoV-19/USA/WA-S16478/2022	Omicron	EPI_ISL_9060534	OM364051
hCoV-19/USA/WA-S16702/2022	Omicron	EPI_ISL_9271905	OM445369
hCoV-19/USA/WA-S16574/2022	Omicron	EPI_ISL_9271778	OM445265
hCoV-19/USA/WA-S16493/2022	Omicron	EPI_ISL_9060549	OM364066
hCoV-19/USA/WA-S16695/2022	Omicron	EPI_ISL_9271898	OM445362
hCoV-19/USA/WA-S16489/2022	Omicron	EPI_ISL_9060545	OM364062
hCoV-19/USA/WA-S16703/2022	Omicron	EPI_ISL_9271906	OM445370
hCoV-19/USA/WA-S16383/2022	Omicron	EPI_ISL_9060439	OM363991
hCoV-19/USA/WA-S16501/2022	Omicron	EPI_ISL_9060557	OM364074

hCoV-19/USA/WA-S16603/2022	Omicron	EPI_ISL_9271808	OM445274
hCoV-19/USA/WA-S16402/2022	Omicron	EPI_ISL_9060458	OM364010
hCoV-19/USA/WA-S16487/2022	Omicron	EPI_ISL_9060543	OM364060
hCoV-19/USA/WA-S16494/2022	Omicron	EPI_ISL_9060550	OM364067
hCoV-19/USA/WA-S16481/2022	Omicron	EPI_ISL_9060537	OM364054
hCoV-19/USA/WA-S16480/2022	Omicron	EPI_ISL_9060536	OM364053
hCoV-19/USA/WA-S16749/2022	Omicron	EPI_ISL_9271952	OM445411
hCoV-19/USA/WA-S16711/2022	Omicron	EPI_ISL_9271914	OM445377
hCoV-19/USA/WA-S16500/2022	Omicron	EPI_ISL_9060556	OM364073
hCoV-19/USA/WA-S16495/2022	Omicron	EPI_ISL_9060551	OM364068
hCoV-19/USA/WA-S16779/2022	Omicron	EPI_ISL_9271981	OM445425
hCoV-19/USA/WA-S16488/2022	Omicron	EPI_ISL_9060544	OM364061
hCoV-19/USA/WA-S16784/2022	Omicron	EPI_ISL_9271986	OM445430
hCoV-19/USA/WA-S16700/2022	Omicron	EPI_ISL_9271903	OM445367
hCoV-19/USA/WA-S16492/2022	Omicron	EPI_ISL_9060548	OM364065
hCoV-19/USA/WA-S16482/2022	Omicron	EPI_ISL_9060538	OM364055
hCoV-19/USA/WA-S16484/2022	Omicron	EPI_ISL_9060540	OM364057
hCoV-19/USA/WA-S16483/2022	Omicron	EPI_ISL_9060539	OM364056
hCoV-19/USA/WA-S16477/2022	Omicron	EPI_ISL_9060533	OM364050
hCoV-19/USA/WA-S16708/2022	Omicron	EPI_ISL_9271911	OM445374
hCoV-19/USA/WA-S16710/2022	Omicron	EPI_ISL_9271913	OM445376
hCoV-19/USA/WA-S16737/2022	Omicron	EPI_ISL_9271940	OM445403
hCoV-19/USA/WA-S16788/2022	Omicron	EPI_ISL_9271990	OM445434
hCoV-19/USA/WA-S16403/2022	Omicron	EPI_ISL_9060459	OM364011
hCoV-19/USA/WA-S16412/2022	Omicron	EPI_ISL_9060468	OM364020
hCoV-19/USA/WA-S16706/2022	Omicron	EPI_ISL_9271909	OM445372
hCoV-19/USA/WA-S16496/2022	Omicron	EPI_ISL_9060552	OM364069
hCoV-19/USA/WA-S16406/2022	Omicron	EPI_ISL_9060462	OM364014
hCoV-19/USA/WA-S16609/2022	Omicron	EPI_ISL_9271813	OM445279
hCoV-19/USA/WA-S16533/2022	Omicron	EPI_ISL_9060589	OM364078
hCoV-19/USA/WA-S16608/2022	Omicron	EPI_ISL_9271812	OM445278
hCoV-19/USA/WA-S16699/2022	Omicron	EPI_ISL_9271902	OM445366
hCoV-19/USA/WA-S16499/2022	Omicron	EPI_ISL_9060555	OM364072
hCoV-19/USA/WA-S16701/2022	Omicron	EPI_ISL_9271904	OM445368
hCoV-19/USA/WA-S16705/2022	Omicron	EPI_ISL_9271908	OP208800
hCoV-19/USA/WA-S16720/2022	Omicron	EPI_ISL_9271923	OM445386
hCoV-19/USA/WA-S16651/2022	Omicron	EPI_ISL_9271855	OM445321
hCoV-19/USA/WA-S16709/2022	Omicron	EPI_ISL_9271912	OM445375
hCoV-19/USA/WA-S16694/2022	Omicron	EPI_ISL_9271897	OM445361
hCoV-19/USA/WA-S16644/2022	Omicron	EPI_ISL_9271848	OM445314
hCoV-19/USA/WA-S16670/2022	Omicron	EPI_ISL_9271874	OM445338
hCoV-19/USA/WA-S16666/2022	Omicron	EPI_ISL_9271870	OP209793
hCoV-19/USA/WA-S16689/2022	Omicron	EPI_ISL_9271892	OM445356
hCoV-19/USA/WA-S16639/2022	Omicron	EPI_ISL_9271843	OM445309
hCoV-19/USA/WA-S16680/2022	Omicron	EPI_ISL_9271884	OM445348
hCoV-19/USA/WA-S16731/2022	Omicron	EPI_ISL_9271934	OM445397
hCoV-19/USA/WA-S16658/2022	Omicron	EPI_ISL_9271862	OM445328
hCoV-19/USA/WA-S16636/2022	Omicron	EPI_ISL_9271840	OM445306
hCoV-19/USA/WA-S16740/2022	Omicron	EPI_ISL_9271943	OM445406
hCoV-19/USA/WA-S16652/2022	Omicron	EPI_ISL_9271856	OM445322
hCoV-19/USA/WA-S16631/2022	Omicron	EPI_ISL_9271835	OM445301

hCoV-19/USA/WA-S16677/2022	Omicron	EPI_ISL_9271881	OM445345
hCoV-19/USA/WA-S16723/2022	Omicron	EPI_ISL_9271926	OM445389
hCoV-19/USA/WA-S16643/2022	Omicron	EPI_ISL_9271847	OM445313
hCoV-19/USA/WA-S16685/2022	Omicron	EPI_ISL_9271888	OM445352
hCoV-19/USA/WA-S16602/2022	Omicron	EPI_ISL_9271807	OM445273
hCoV-19/USA/WA-S16714/2022	Omicron	EPI_ISL_9271917	OM445380
hCoV-19/USA/WA-S16645/2022	Omicron	EPI_ISL_9271849	OM445315
hCoV-19/USA/WA-S16687/2022	Omicron	EPI_ISL_9271890	OM445354
hCoV-19/USA/WA-S16729/2022	Omicron	EPI_ISL_9271932	OM445395
hCoV-19/USA/WA-S16726/2022	Omicron	EPI_ISL_9271929	OM445392
hCoV-19/USA/WA-S16939/2022	Omicron	EPI_ISL_9346759	OM457877
hCoV-19/USA/WA-S16656/2022	Omicron	EPI_ISL_9271860	OM445326
hCoV-19/USA/WA-S16668/2022	Omicron	EPI_ISL_9271872	OM445336
hCoV-19/USA/WA-S16647/2022	Omicron	EPI_ISL_9271851	OM445317
hCoV-19/USA/WA-S16718/2022	Omicron	EPI_ISL_9271921	OM445384
hCoV-19/USA/WA-S16755/2022	Omicron	EPI_ISL_9271958	OM445412
hCoV-19/USA/WA-S16657/2022	Omicron	EPI_ISL_9271861	OM445327
hCoV-19/USA/WA-S16713/2022	Omicron	EPI_ISL_9271916	OM445379
hCoV-19/USA/WA-S16662/2022	Omicron	EPI_ISL_9271866	OM445332
hCoV-19/USA/WA-S16686/2022	Omicron	EPI_ISL_9271889	OM445353
hCoV-19/USA/WA-S16655/2022	Omicron	EPI_ISL_9271859	OM445325
hCoV-19/USA/WA-S16722/2022	Omicron	EPI_ISL_9271925	OM445388
hCoV-19/USA/WA-S16642/2022	Omicron	EPI_ISL_9271846	OM445312
hCoV-19/USA/WA-S16673/2022	Omicron	EPI_ISL_9271877	OM445341
hCoV-19/USA/WA-S16716/2022	Omicron	EPI_ISL_9271919	OM445382
hCoV-19/USA/WA-S16691/2022	Omicron	EPI_ISL_9271894	OM445358
hCoV-19/USA/WA-S16746/2022	Omicron	EPI_ISL_9271949	OM445410
hCoV-19/USA/WA-S16665/2022	Omicron	EPI_ISL_9271869	OM445335
hCoV-19/USA/WA-S16635/2022	Omicron	EPI_ISL_9271839	OM445305
hCoV-19/USA/WA-S16735/2022	Omicron	EPI_ISL_9271938	OM445401
hCoV-19/USA/WA-S16653/2022	Omicron	EPI_ISL_9271857	OM445323
hCoV-19/USA/WA-S16661/2022	Omicron	EPI_ISL_9271865	OM445331
hCoV-19/USA/WA-S16684/2022	Omicron	EPI_ISL_9271887	OM445351
hCoV-19/USA/WA-S16734/2022	Omicron	EPI_ISL_9271937	OM445400
hCoV-19/USA/WA-S16641/2022	Omicron	EPI_ISL_9271845	OM445311
hCoV-19/USA/WA-S16675/2022	Omicron	EPI_ISL_9271879	OM445343
hCoV-19/USA/WA-S16727/2022	Omicron	EPI_ISL_9271930	OM445393
hCoV-19/USA/WA-S16630/2022	Omicron	EPI_ISL_9271834	OM445300
hCoV-19/USA/WA-S16672/2022	Omicron	EPI_ISL_9271876	OM445340
hCoV-19/USA/WA-S16773/2022	Omicron	EPI_ISL_9271975	OM445419
hCoV-19/USA/WA-S16650/2022	Omicron	EPI_ISL_9271854	OM445320
hCoV-19/USA/WA-S16724/2022	Omicron	EPI_ISL_9271927	OM445390
hCoV-19/USA/WA-S16640/2022	Omicron	EPI_ISL_9271844	OM445310
hCoV-19/USA/WA-S16936/2022	Omicron	EPI_ISL_9346756	OM457874
hCoV-19/USA/WA-S16671/2022	Omicron	EPI_ISL_9271875	OM445339
hCoV-19/USA/WA-S16638/2022	Omicron	EPI_ISL_9271842	OM445308
hCoV-19/USA/WA-S16715/2022	Omicron	EPI_ISL_9271918	OM445381
hCoV-19/USA/WA-S16679/2022	Omicron	EPI_ISL_9271883	OM445347
hCoV-19/USA/WA-S16772/2022	Omicron	EPI_ISL_9271974	OM445418
hCoV-19/USA/WA-S16771/2022	Omicron	EPI_ISL_9271973	OM445417
hCoV-19/USA/WA-S16634/2022	Omicron	EPI_ISL_9271838	OM445304

hCoV-19/USA/WA-S16738/2022	Omicron	EPI_ISL_9271941	OM445404
hCoV-19/USA/WA-S16693/2022	Omicron	EPI_ISL_9271896	OM445360
hCoV-19/USA/WA-S16736/2022	Omicron	EPI_ISL_9271939	OM445402
hCoV-19/USA/WA-S16637/2022	Omicron	EPI_ISL_9271841	OM445307
hCoV-19/USA/WA-S16654/2022	Omicron	EPI_ISL_9271858	OM445324
hCoV-19/USA/WA-S16676/2022	Omicron	EPI_ISL_9271880	OM445344
hCoV-19/USA/WA-S16742/2022	Omicron	EPI_ISL_9271945	OM445408
hCoV-19/USA/WA-S16659/2022	Omicron	EPI_ISL_9271863	OM445329
hCoV-19/USA/WA-S16725/2022	Omicron	EPI_ISL_9271928	OM445391
hCoV-19/USA/WA-S16646/2022	Omicron	EPI_ISL_9271850	OM445316
hCoV-19/USA/WA-S16717/2022	Omicron	EPI_ISL_9271920	OM445383
hCoV-19/USA/WA-S16601/2022	Omicron	EPI_ISL_9271806	OM445272
hCoV-19/USA/WA-S16758/2022	Omicron	EPI_ISL_9271960	OM445413
hCoV-19/USA/WA-S16789/2022	Omicron	EPI_ISL_9271991	OM445435
hCoV-19/USA/WA-S16600/2022	Omicron	EPI_ISL_9271805	OM445271
hCoV-19/USA/WA-S16607/2022	Omicron	EPI_ISL_9271811	OM445277
hCoV-19/USA/WA-S16938/2022	Omicron	EPI_ISL_9346758	OM457876
hCoV-19/USA/WA-S16935/2022	Omicron	EPI_ISL_9346755	OM457873
hCoV-19/USA/WA-S16732/2022	Omicron	EPI_ISL_9271935	OM445398
hCoV-19/USA/WA-S16741/2022	Omicron	EPI_ISL_9271944	OM445407
hCoV-19/USA/WA-S16664/2022	Omicron	EPI_ISL_9271868	OM445334
hCoV-19/USA/WA-S16719/2022	Omicron	EPI_ISL_9271922	OM445385
hCoV-19/USA/WA-S16629/2022	Omicron	EPI_ISL_9271833	OM445299
hCoV-19/USA/WA-S16940/2022	Omicron	EPI_ISL_9346760	OM457878
hCoV-19/USA/WA-S16777/2022	Omicron	EPI_ISL_9271979	OM445423
hCoV-19/USA/WA-S16632/2022	Omicron	EPI_ISL_9271836	OM445302
hCoV-19/USA/WA-S16663/2022	Omicron	EPI_ISL_9271867	OM445333
hCoV-19/USA/WA-S16667/2022	Omicron	EPI_ISL_9271871	OP209795
hCoV-19/USA/WA-S16688/2022	Omicron	EPI_ISL_9271891	OM445355
hCoV-19/USA/WA-S16678/2022	Omicron	EPI_ISL_9271882	OM445346
hCoV-19/USA/WA-S16682/2022	Omicron	EPI_ISL_9271885	OM445349
hCoV-19/USA/WA-S16669/2022	Omicron	EPI_ISL_9271873	OM445337
hCoV-19/USA/WA-S16633/2022	Omicron	EPI_ISL_9271837	OM445303
hCoV-19/USA/WA-S16739/2022	Omicron	EPI_ISL_9271942	OM445405
hCoV-19/USA/WA-S16649/2022	Omicron	EPI_ISL_9271853	OM445319
hCoV-19/USA/WA-S16730/2022	Omicron	EPI_ISL_9271933	OM445396
hCoV-19/USA/WA-S16683/2022	Omicron	EPI_ISL_9271886	OM445350
hCoV-19/USA/WA-S16660/2022	Omicron	EPI_ISL_9271864	OM445330
hCoV-19/USA/WA-S16721/2022	Omicron	EPI_ISL_9271924	OM445387
hCoV-19/USA/WA-S16743/2022	Omicron	EPI_ISL_9271946	OM445409
hCoV-19/USA/WA-S16928/2022	Omicron	EPI_ISL_9346748	OM457866
hCoV-19/USA/WA-S16929/2022	Omicron	EPI_ISL_9346749	OM457867
hCoV-19/USA/WA-S16806/2022	Omicron	EPI_ISL_9272008	OM445452
hCoV-19/USA/WA-S16801/2022	Omicron	EPI_ISL_9272003	OM445447
hCoV-19/USA/WA-S16920/2022	Omicron	EPI_ISL_9346740	OM457858
hCoV-19/USA/WA-S16805/2022	Omicron	EPI_ISL_9272007	OM445451
hCoV-19/USA/WA-S16812/2022	Omicron	EPI_ISL_9272014	OM445458
hCoV-19/USA/WA-S16949/2022	Omicron	EPI_ISL_9346769	OM457887
hCoV-19/USA/WA-S16912/2022	Omicron	EPI_ISL_9346732	OM457850
hCoV-19/USA/WA-S16921/2022	Omicron	EPI_ISL_9346741	OM457859
hCoV-19/USA/WA-S16917/2022	Omicron	EPI_ISL_9346737	OM457855

hCoV-19/USA/WA-S16924/2022	Omicron	EPI_ISL_9346744	OM457862
hCoV-19/USA/WA-S16889/2022	Omicron	EPI_ISL_9346709	OM457827
hCoV-19/USA/WA-S16902/2022	Omicron	EPI_ISL_9346722	OM457840
hCoV-19/USA/WA-S16787/2022	Omicron	EPI_ISL_9271989	OM445433
hCoV-19/USA/WA-S16888/2022	Omicron	EPI_ISL_9346708	OM457826
hCoV-19/USA/WA-S16927/2022	Omicron	EPI_ISL_9346747	OM457865
hCoV-19/USA/WA-S16809/2022	Omicron	EPI_ISL_9272011	OM445455
hCoV-19/USA/WA-S16887/2022	Omicron	EPI_ISL_9346707	OM457825
hCoV-19/USA/WA-S16800/2022	Omicron	EPI_ISL_9272002	OM445446
hCoV-19/USA/WA-S16794/2022	Omicron	EPI_ISL_9271996	OM445440
hCoV-19/USA/WA-S16891/2022	Omicron	EPI_ISL_9346711	OM457829
hCoV-19/USA/WA-S16907/2022	Omicron	EPI_ISL_9346727	OM457845
hCoV-19/USA/WA-S16804/2022	Omicron	EPI_ISL_9272006	OM445450
hCoV-19/USA/WA-S16796/2022	Omicron	EPI_ISL_9271998	OM445442
hCoV-19/USA/WA-S16914/2022	Omicron	EPI_ISL_9346734	OM457852
hCoV-19/USA/WA-S16904/2022	Omicron	EPI_ISL_9346724	OM457842
hCoV-19/USA/WA-S16944/2022	Omicron	EPI_ISL_9346764	OM457882
hCoV-19/USA/WA-S16910/2022	Omicron	EPI_ISL_9346730	OM457848
hCoV-19/USA/WA-S16795/2022	Omicron	EPI_ISL_9271997	OM445441
hCoV-19/USA/WA-S16892/2022	Omicron	EPI_ISL_9346712	OM457830
hCoV-19/USA/WA-S16895/2022	Omicron	EPI_ISL_9346715	OM457833
hCoV-19/USA/WA-S16819/2022	Omicron	EPI_ISL_9272021	OM445462
hCoV-19/USA/WA-S16802/2022	Omicron	EPI_ISL_9272004	OM445448
hCoV-19/USA/WA-S16790/2022	Omicron	EPI_ISL_9271992	OM445436
hCoV-19/USA/WA-S16896/2022	Omicron	EPI_ISL_9346716	OM457834
hCoV-19/USA/WA-S16786/2022	Omicron	EPI_ISL_9271988	OM445432
hCoV-19/USA/WA-S16915/2022	Omicron	EPI_ISL_9346735	OM457853
hCoV-19/USA/WA-S16948/2022	Omicron	EPI_ISL_9346768	OM457886
hCoV-19/USA/WA-S16926/2022	Omicron	EPI_ISL_9346746	OM457864
hCoV-19/USA/WA-S16781/2022	Omicron	EPI_ISL_9271983	OM445427
hCoV-19/USA/WA-S16908/2022	Omicron	EPI_ISL_9346728	OM457846
hCoV-19/USA/WA-S16923/2022	Omicron	EPI_ISL_9346743	OM457861
hCoV-19/USA/WA-S16916/2022	Omicron	EPI_ISL_9346736	OM457854
hCoV-19/USA/WA-S16922/2022	Omicron	EPI_ISL_9346742	OM457860
hCoV-19/USA/WA-S16899/2022	Omicron	EPI_ISL_9346719	OM457837
hCoV-19/USA/WA-S16146/2022	Omicron	EPI_ISL_8822465	OM296952
hCoV-19/USA/WA-S16893/2022	Omicron	EPI_ISL_9346713	OM457831
hCoV-19/USA/WA-S16815/2022	Omicron	EPI_ISL_9272017	OM445461
hCoV-19/USA/WA-S16945/2022	Omicron	EPI_ISL_9346765	OM457883
hCoV-19/USA/WA-S16947/2022	Omicron	EPI_ISL_9346767	OM457885
hCoV-19/USA/WA-S16937/2022	Omicron	EPI_ISL_9346757	OM457875
hCoV-19/USA/WA-S16943/2022	Omicron	EPI_ISL_9346763	OM457881
hCoV-19/USA/WA-S16811/2022	Omicron	EPI_ISL_9272013	OM445457
hCoV-19/USA/WA-S16913/2022	Omicron	EPI_ISL_9346733	OM457851
hCoV-19/USA/WA-S16797/2022	Omicron	EPI_ISL_9271999	OM445443
hCoV-19/USA/WA-S16903/2022	Omicron	EPI_ISL_9346723	OM457841
hCoV-19/USA/WA-S16953/2022	Omicron	EPI_ISL_9346773	OM457891
hCoV-19/USA/WA-S16776/2022	Omicron	EPI_ISL_9271978	OM445422
hCoV-19/USA/WA-S16918/2022	Omicron	EPI_ISL_9346738	OM457856
hCoV-19/USA/WA-S16909/2022	Omicron	EPI_ISL_9346729	OM457847
hCoV-19/USA/WA-S16911/2022	Omicron	EPI_ISL_9346731	OM457849

hCoV-19/USA/WA-S16930/2022	Omicron	EPI_ISL_9346750	OM457868
hCoV-19/USA/WA-S16932/2022	Omicron	EPI_ISL_9346752	OM457870
hCoV-19/USA/WA-S16898/2022	Omicron	EPI_ISL_9346718	OM457836
hCoV-19/USA/WA-S16785/2022	Omicron	EPI_ISL_9271987	OM445431
hCoV-19/USA/WA-S16958/2022	Omicron	EPI_ISL_9346778	OM457896
hCoV-19/USA/WA-S16793/2022	Omicron	EPI_ISL_9271995	OM445439
hCoV-19/USA/WA-S16931/2022	Omicron	EPI_ISL_9346751	OM457869
hCoV-19/USA/WA-S16950/2022	Omicron	EPI_ISL_9346770	OM457888
hCoV-19/USA/WA-S16952/2022	Omicron	EPI_ISL_9346772	OM457890
hCoV-19/USA/WA-S16897/2022	Omicron	EPI_ISL_9346717	OM457835
hCoV-19/USA/WA-S16957/2022	Omicron	EPI_ISL_9346777	OM457895
hCoV-19/USA/WA-S16807/2022	Omicron	EPI_ISL_9272009	OM445453
hCoV-19/USA/WA-S16810/2022	Omicron	EPI_ISL_9272012	OM445456
hCoV-19/USA/WA-S16813/2022	Omicron	EPI_ISL_9272015	OM445459
hCoV-19/USA/WA-S16925/2022	Omicron	EPI_ISL_9346745	OM457863
hCoV-19/USA/WA-S16808/2022	Omicron	EPI_ISL_9272010	OM445454
hCoV-19/USA/WA-S16890/2022	Omicron	EPI_ISL_9346710	OM457828
hCoV-19/USA/WA-S16799/2022	Omicron	EPI_ISL_9272001	OM445445
hCoV-19/USA/WA-S16941/2022	Omicron	EPI_ISL_9346761	OM457879
hCoV-19/USA/WA-S16942/2022	Omicron	EPI_ISL_9346762	OM457880
hCoV-19/USA/WA-S16783/2022	Omicron	EPI_ISL_9271985	OM445429
hCoV-19/USA/WA-S16774/2022	Omicron	EPI_ISL_9271976	OM445420
hCoV-19/USA/WA-S16946/2022	Omicron	EPI_ISL_9346766	OM457884
hCoV-19/USA/WA-S16143/2022	Omicron	EPI_ISL_8822462	OM296949
hCoV-19/USA/WA-S16919/2022	Omicron	EPI_ISL_9346739	OM457857
hCoV-19/USA/WA-S16798/2022	Omicron	EPI_ISL_9272000	OM445444
hCoV-19/USA/WA-S16905/2022	Omicron	EPI_ISL_9346725	OM457843
hCoV-19/USA/WA-S16934/2022	Omicron	EPI_ISL_9346754	OM457872
hCoV-19/USA/WA-S16906/2022	Omicron	EPI_ISL_9346726	OM457844
hCoV-19/USA/WA-S16954/2022	Omicron	EPI_ISL_9346774	OM457892
hCoV-19/USA/WA-S16901/2022	Omicron	EPI_ISL_9346721	OM457839
hCoV-19/USA/WA-S16956/2022	Omicron	EPI_ISL_9346776	OM457894
hCoV-19/USA/WA-S16073/2022	Omicron	EPI_ISL_8822390	OM296879
hCoV-19/USA/WA-S16122/2022	Omicron	EPI_ISL_8822440	OM296928
hCoV-19/USA/WA-S16131/2022	Omicron	EPI_ISL_8822450	OM296937
hCoV-19/USA/WA-S16822/2022	Omicron	EPI_ISL_9272024	OM445463
hCoV-19/USA/WA-S16971/2022	Omicron	EPI_ISL_9346791	OM457909
hCoV-19/USA/WA-S16107/2022	Omicron	EPI_ISL_8822425	OM296913
hCoV-19/USA/WA-S16092/2022	Omicron	EPI_ISL_8822410	OM296898
hCoV-19/USA/WA-S16105/2022	Omicron	EPI_ISL_8822423	OM296911
hCoV-19/USA/WA-S16114/2022	Omicron	EPI_ISL_8822432	OM296920
hCoV-19/USA/WA-S16951/2022	Omicron	EPI_ISL_9346771	OM457889
hCoV-19/USA/WA-S16973/2022	Omicron	EPI_ISL_9346793	OM457911
hCoV-19/USA/WA-S16099/2022	Omicron	EPI_ISL_8822417	OM296905
hCoV-19/USA/WA-S16968/2022	Omicron	EPI_ISL_9346788	OM457906
hCoV-19/USA/WA-S16963/2022	Omicron	EPI_ISL_9346783	OM457901
hCoV-19/USA/WA-S16961/2022	Omicron	EPI_ISL_9346781	OM457899
hCoV-19/USA/WA-S16076/2022	Omicron	EPI_ISL_8822393	OM296882
hCoV-19/USA/WA-S16138/2022	Omicron	EPI_ISL_8822457	OM296944
hCoV-19/USA/WA-S16972/2022	Omicron	EPI_ISL_9346792	OM457910
hCoV-19/USA/WA-S16083/2022	Omicron	EPI_ISL_8822400	OM296889

hCoV-19/USA/WA-S16115/2022	Omicron	EPI_ISL_8822433	OM296921
hCoV-19/USA/WA-S16069/2022	Omicron	EPI_ISL_8822386	OM296875
hCoV-19/USA/WA-S16124/2022	Omicron	EPI_ISL_8822442	OM296930
hCoV-19/USA/WA-S16078/2022	Omicron	EPI_ISL_8822395	OM296884
hCoV-19/USA/WA-S16127/2022	Omicron	EPI_ISL_8822445	OM296933
hCoV-19/USA/WA-S16098/2022	Omicron	EPI_ISL_8822416	OM296904
hCoV-19/USA/WA-S16071/2022	Omicron	EPI_ISL_8822388	OM296877
hCoV-19/USA/WA-S16100/2022	Omicron	EPI_ISL_8822418	OM296906
hCoV-19/USA/WA-S16088/2022	Omicron	EPI_ISL_8822405	OM296894
hCoV-19/USA/WA-S16960/2022	Omicron	EPI_ISL_9346780	OM457898
hCoV-19/USA/WA-S16068/2022	Omicron	EPI_ISL_8822385	OM296874
hCoV-19/USA/WA-S16962/2022	Omicron	EPI_ISL_9346782	OM457900
hCoV-19/USA/WA-S16082/2022	Omicron	EPI_ISL_8822399	OM296888
hCoV-19/USA/WA-S16964/2022	Omicron	EPI_ISL_9346784	OM457902
hCoV-19/USA/WA-S16976/2022	Omicron	EPI_ISL_9346796	OM457914
hCoV-19/USA/WA-S16085/2022	Omicron	EPI_ISL_8822402	OM296891
hCoV-19/USA/WA-S16081/2022	Omicron	EPI_ISL_8822398	OM296887
hCoV-19/USA/WA-S16975/2022	Omicron	EPI_ISL_9346795	OM457913
hCoV-19/USA/WA-S16140/2022	Omicron	EPI_ISL_8822459	OM296946
hCoV-19/USA/WA-S16113/2022	Omicron	EPI_ISL_8822431	OM296919
hCoV-19/USA/WA-S16149/2022	Omicron	EPI_ISL_8822468	OM296955
hCoV-19/USA/WA-S16104/2022	Omicron	EPI_ISL_8822422	OM296910
hCoV-19/USA/WA-S16074/2022	Omicron	EPI_ISL_8822391	OM296880
hCoV-19/USA/WA-S16995/2022	Omicron	EPI_ISL_9346815	OM457933
hCoV-19/USA/WA-S16130/2022	Omicron	EPI_ISL_8822449	OM296936
hCoV-19/USA/WA-S16102/2022	Omicron	EPI_ISL_8822420	OM296908
hCoV-19/USA/WA-S16118/2022	Omicron	EPI_ISL_8822436	OM296924
hCoV-19/USA/WA-S16066/2022	Omicron	EPI_ISL_8822383	OM296872
hCoV-19/USA/WA-S16996/2022	Omicron	EPI_ISL_9346816	OM457934
hCoV-19/USA/WA-S16095/2022	Omicron	EPI_ISL_8822413	OM296901
hCoV-19/USA/WA-S16119/2022	Omicron	EPI_ISL_8822437	OM296925
hCoV-19/USA/WA-S16141/2022	Omicron	EPI_ISL_8822460	OM296947
hCoV-19/USA/WA-S16129/2022	Omicron	EPI_ISL_8822448	OM296935
hCoV-19/USA/WA-S16955/2022	Omicron	EPI_ISL_9346775	OM457893
hCoV-19/USA/WA-S16080/2022	Omicron	EPI_ISL_8822397	OM296886
hCoV-19/USA/WA-S16086/2022	Omicron	EPI_ISL_8822403	OM296892
hCoV-19/USA/WA-S16991/2022	Omicron	EPI_ISL_9346811	OM457929
hCoV-19/USA/WA-S16101/2022	Omicron	EPI_ISL_8822419	OM296907
hCoV-19/USA/WA-S16089/2022	Omicron	EPI_ISL_8822406	OM296895
hCoV-19/USA/WA-S16148/2022	Omicron	EPI_ISL_8822467	OM296954
hCoV-19/USA/WA-S16090/2022	Omicron	EPI_ISL_8822407	OM296896
hCoV-19/USA/WA-S16064/2022	Omicron	EPI_ISL_8822381	OM296870
hCoV-19/USA/WA-S16993/2022	Omicron	EPI_ISL_9346813	OM457931
hCoV-19/USA/WA-S16132/2022	Omicron	EPI_ISL_8822451	OM296938
hCoV-19/USA/WA-S16147/2022	Omicron	EPI_ISL_8822466	OM296953
hCoV-19/USA/WA-S16992/2022	Omicron	EPI_ISL_9346812	OM457930
hCoV-19/USA/WA-S16075/2022	Omicron	EPI_ISL_8822392	OM296881
hCoV-19/USA/WA-S16094/2022	Omicron	EPI_ISL_8822412	OM296900
hCoV-19/USA/WA-S16109/2022	Omicron	EPI_ISL_8822427	OM296915
hCoV-19/USA/WA-S16096/2022	Omicron	EPI_ISL_8822414	OM296902
hCoV-19/USA/WA-S16150/2022	Omicron	EPI_ISL_8822469	OM296956

hCoV-19/USA/WA-S16990/2022	Omicron	EPI_ISL_9346810	OM457928
hCoV-19/USA/WA-S16091/2022	Omicron	EPI_ISL_8822409	OM296897
hCoV-19/USA/WA-S16989/2022	Omicron	EPI_ISL_9346809	OM457927
hCoV-19/USA/WA-S16959/2022	Omicron	EPI_ISL_9346779	OM457897
hCoV-19/USA/WA-S16110/2022	Omicron	EPI_ISL_8822428	OM296916
hCoV-19/USA/WA-S16065/2022	Omicron	EPI_ISL_8822382	OM296871
hCoV-19/USA/WA-S16120/2022	Omicron	EPI_ISL_8822438	OM296926
hCoV-19/USA/WA-S17008/2022	Omicron	EPI_ISL_9346828	OM457946
hCoV-19/USA/WA-S16144/2022	Omicron	EPI_ISL_8822463	OM296950
hCoV-19/USA/WA-S16103/2022	Omicron	EPI_ISL_8822421	OM296909
hCoV-19/USA/WA-S16994/2022	Omicron	EPI_ISL_9346814	OM457932
hCoV-19/USA/WA-S16067/2022	Omicron	EPI_ISL_8822384	OM296873
hCoV-19/USA/WA-S16974/2022	Omicron	EPI_ISL_9346794	OM457912
hCoV-19/USA/WA-S16135/2022	Omicron	EPI_ISL_8822454	OM296941
hCoV-19/USA/WA-S16125/2022	Omicron	EPI_ISL_8822443	OM296931
hCoV-19/USA/WA-S16097/2022	Omicron	EPI_ISL_8822415	OM296903
hCoV-19/USA/WA-S16077/2022	Omicron	EPI_ISL_8822394	OM296883
hCoV-19/USA/WA-S16072/2022	Omicron	EPI_ISL_8822389	OM296878
hCoV-19/USA/WA-S16087/2022	Omicron	EPI_ISL_8822404	OM296893
hCoV-19/USA/WA-S16133/2022	Omicron	EPI_ISL_8822452	OM296939
hCoV-19/USA/WA-S16070/2022	Omicron	EPI_ISL_8822387	OM296876
hCoV-19/USA/WA-S16111/2022	Omicron	EPI_ISL_8822429	OM296917
hCoV-19/USA/WA-S16117/2022	Omicron	EPI_ISL_8822435	OM296923
hCoV-19/USA/WA-S16112/2022	Omicron	EPI_ISL_8822430	OM296918
hCoV-19/USA/WA-S16966/2022	Omicron	EPI_ISL_9346786	OM457904
hCoV-19/USA/WA-S16128/2022	Omicron	EPI_ISL_8822446	OM296934
hCoV-19/USA/WA-S16969/2022	Omicron	EPI_ISL_9346789	OM457907
hCoV-19/USA/WA-S16965/2022	Omicron	EPI_ISL_9346785	OM457903
hCoV-19/USA/WA-S16093/2022	Omicron	EPI_ISL_8822411	OM296899
hCoV-19/USA/WA-S16079/2022	Omicron	EPI_ISL_8822396	OM296885
hCoV-19/USA/WA-S16967/2022	Omicron	EPI_ISL_9346787	OM457905
hCoV-19/USA/WA-S16145/2022	Omicron	EPI_ISL_8822464	OM296951
hCoV-19/USA/WA-S16121/2022	Omicron	EPI_ISL_8822439	OM296927
hCoV-19/USA/WA-S16108/2022	Omicron	EPI_ISL_8822426	OM296914
hCoV-19/USA/WA-S16136/2022	Omicron	EPI_ISL_8822455	OM296942
hCoV-19/USA/WA-S16116/2022	Omicron	EPI_ISL_8822434	OM296922
hCoV-19/USA/WA-S16123/2022	Omicron	EPI_ISL_8822441	OM296929
hCoV-19/USA/WA-S16978/2022	Omicron	EPI_ISL_9346798	OM457916
hCoV-19/USA/WA-S17001/2022	Omicron	EPI_ISL_9346821	OM457939
hCoV-19/USA/WA-S16981/2022	Omicron	EPI_ISL_9346801	OM457919
hCoV-19/USA/WA-S17002/2022	Omicron	EPI_ISL_9346822	OM457940
hCoV-19/USA/WA-S17007/2022	Omicron	EPI_ISL_9346827	OM457945
hCoV-19/USA/WA-S17012/2022	Omicron	EPI_ISL_9346832	OM457950
hCoV-19/USA/WA-S16983/2022	Omicron	EPI_ISL_9346803	OM457921
hCoV-19/USA/WA-S16999/2022	Omicron	EPI_ISL_9346819	OM457937
hCoV-19/USA/WA-S17000/2022	Omicron	EPI_ISL_9346820	OM457938
hCoV-19/USA/WA-S16982/2022	Omicron	EPI_ISL_9346802	OM457920
hCoV-19/USA/WA-S17003/2022	Omicron	EPI_ISL_9346823	OM457941
hCoV-19/USA/WA-S16980/2022	Omicron	EPI_ISL_9346800	OM457918
hCoV-19/USA/WA-S17011/2022	Omicron	EPI_ISL_9346831	OM457949
hCoV-19/USA/WA-S16979/2022	Omicron	EPI_ISL_9346799	OM457917

hCoV-19/USA/WA-S16987/2022	Omicron	EPI_ISL_9346807	OM457925
hCoV-19/USA/WA-S16977/2022	Omicron	EPI_ISL_9346797	OM457915
hCoV-19/USA/WA-S16985/2022	Omicron	EPI_ISL_9346805	OM457923
hCoV-19/USA/WA-S17004/2022	Omicron	EPI_ISL_9346824	OM457942
hCoV-19/USA/WA-S17005/2022	Omicron	EPI_ISL_9346825	OM457943
hCoV-19/USA/WA-S17009/2022	Omicron	EPI_ISL_9346829	OM457947
hCoV-19/USA/WA-S17010/2022	Omicron	EPI_ISL_9346830	OM457948
hCoV-19/USA/WA-S16988/2022	Omicron	EPI_ISL_9346808	OM457926
hCoV-19/USA/WA-S16997/2022	Omicron	EPI_ISL_9346817	OM457935
hCoV-19/USA/WA-S17006/2022	Omicron	EPI_ISL_9346826	OM457944
hCoV-19/USA/WA-S17016/2022	Omicron	EPI_ISL_9346837	OM457954
hCoV-19/USA/WA-S17038/2022	Omicron	EPI_ISL_9346859	OM457976
hCoV-19/USA/WA-S17023/2022	Omicron	EPI_ISL_9346844	OM457961
hCoV-19/USA/WA-S17042/2022	Omicron	EPI_ISL_9346863	OM457980
hCoV-19/USA/WA-S17028/2022	Omicron	EPI_ISL_9346849	OM457966
hCoV-19/USA/WA-S16883/2022	Omicron	EPI_ISL_9272085	OM445469
hCoV-19/USA/WA-S17024/2022	Omicron	EPI_ISL_9346845	OM457962
hCoV-19/USA/WA-S17026/2022	Omicron	EPI_ISL_9346847	OM457964
hCoV-19/USA/WA-S17034/2022	Omicron	EPI_ISL_9346855	OM457972
hCoV-19/USA/WA-S17018/2022	Omicron	EPI_ISL_9346839	OM457956
hCoV-19/USA/WA-S17065/2022	Omicron	EPI_ISL_9346885	OM458002
hCoV-19/USA/WA-S17022/2022	Omicron	EPI_ISL_9346843	OM457960
hCoV-19/USA/WA-S17040/2022	Omicron	EPI_ISL_9346861	OM457978
hCoV-19/USA/WA-S17027/2022	Omicron	EPI_ISL_9346848	OM457965
hCoV-19/USA/WA-S17036/2022	Omicron	EPI_ISL_9346857	OM457974
hCoV-19/USA/WA-S16884/2022	Omicron	EPI_ISL_9272086	OM445470
hCoV-19/USA/WA-S17037/2022	Omicron	EPI_ISL_9346858	OM457975
hCoV-19/USA/WA-S17033/2022	Omicron	EPI_ISL_9346854	OM457971
hCoV-19/USA/WA-S17017/2022	Omicron	EPI_ISL_9346838	OM457955
hCoV-19/USA/WA-S17021/2022	Omicron	EPI_ISL_9346842	OM457959
hCoV-19/USA/WA-S17013/2022	Omicron	EPI_ISL_9346833	OM457951
hCoV-19/USA/WA-S17030/2022	Omicron	EPI_ISL_9346851	OM457968
hCoV-19/USA/WA-S17035/2022	Omicron	EPI_ISL_9346856	OM457973
hCoV-19/USA/WA-S16151/2022	Omicron	EPI_ISL_8822470	OM296957
hCoV-19/USA/WA-S17025/2022	Omicron	EPI_ISL_9346846	OM457963
hCoV-19/USA/WA-S17127/2022	Omicron	EPI_ISL_9346947	OM458064
hCoV-19/USA/WA-S16885/2022	Omicron	EPI_ISL_9272087	OM445471
hCoV-19/USA/WA-S17039/2022	Omicron	EPI_ISL_9346860	OM457977
hCoV-19/USA/WA-S17015/2022	Omicron	EPI_ISL_9346836	OM457953
hCoV-19/USA/WA-S16882/2022	Omicron	EPI_ISL_9272084	OM445468
hCoV-19/USA/WA-S17014/2022	Omicron	EPI_ISL_9346834	OM457952
hCoV-19/USA/WA-S17029/2022	Omicron	EPI_ISL_9346850	OM457967
hCoV-19/USA/WA-S17019/2022	Omicron	EPI_ISL_9346840	OM457957
hCoV-19/USA/WA-S17032/2022	Omicron	EPI_ISL_9346853	OM457970
hCoV-19/USA/WA-S17031/2022	Omicron	EPI_ISL_9346852	OM457969
hCoV-19/USA/WA-S17063/2022	Omicron	EPI_ISL_9346883	OM458000
hCoV-19/USA/WA-S17105/2022	Omicron	EPI_ISL_9346925	OM458042
hCoV-19/USA/WA-S17057/2022	Omicron	EPI_ISL_9346878	OM457995
hCoV-19/USA/WA-S17225/2022	Omicron	EPI_ISL_9553010	OM538446
hCoV-19/USA/WA-S17135/2022	Omicron	EPI_ISL_9346955	OM458067
hCoV-19/USA/WA-S17108/2022	Omicron	EPI_ISL_9346928	OM458045

hCoV-19/USA/WA-S17100/2022	Omicron	EPI_ISL_9346920	OM458037
hCoV-19/USA/WA-S17125/2022	Omicron	EPI_ISL_9346945	OM458062
hCoV-19/USA/WA-S17093/2022	Omicron	EPI_ISL_9346913	OM458030
hCoV-19/USA/WA-S17116/2022	Omicron	EPI_ISL_9346936	OM458053
hCoV-19/USA/WA-S17054/2022	Omicron	EPI_ISL_9346875	OM457992
hCoV-19/USA/WA-S17224/2022	Omicron	EPI_ISL_9553009	OM538445
hCoV-19/USA/WA-S17084/2022	Omicron	EPI_ISL_9346904	OM458021
hCoV-19/USA/WA-S17267/2022	Omicron	EPI_ISL_9553052	OM538488
hCoV-19/USA/WA-S17062/2022	Omicron	EPI_ISL_9346882	OM457999
hCoV-19/USA/WA-S17218/2022	Omicron	EPI_ISL_9553003	OM538439
hCoV-19/USA/WA-S17217/2022	Omicron	EPI_ISL_9553002	OM538438
hCoV-19/USA/WA-S17126/2022	Omicron	EPI_ISL_9346946	OM458063
hCoV-19/USA/WA-S17079/2022	Omicron	EPI_ISL_9346899	OM458016
hCoV-19/USA/WA-S17083/2022	Omicron	EPI_ISL_9346903	OM458020
hCoV-19/USA/WA-S17047/2022	Omicron	EPI_ISL_9346868	OM457985
hCoV-19/USA/WA-S17117/2022	Omicron	EPI_ISL_9346937	OM458054
hCoV-19/USA/WA-S17124/2022	Omicron	EPI_ISL_9346944	OM458061
hCoV-19/USA/WA-S17101/2022	Omicron	EPI_ISL_9346921	OM458038
hCoV-19/USA/WA-S17205/2022	Omicron	EPI_ISL_9552990	OM538426
hCoV-19/USA/WA-S17223/2022	Omicron	EPI_ISL_9553008	OM538444
hCoV-19/USA/WA-S17092/2022	Omicron	EPI_ISL_9346912	OM458029
hCoV-19/USA/WA-S17060/2022	Omicron	EPI_ISL_9346881	OM457998
hCoV-19/USA/WA-S17207/2022	Omicron	EPI_ISL_9552992	OM538428
hCoV-19/USA/WA-S17104/2022	Omicron	EPI_ISL_9346924	OM458041
hCoV-19/USA/WA-S17212/2022	Omicron	EPI_ISL_9552997	OM538433
hCoV-19/USA/WA-S17086/2022	Omicron	EPI_ISL_9346906	OM458023
hCoV-19/USA/WA-S17216/2022	Omicron	EPI_ISL_9553001	OM538437
hCoV-19/USA/WA-S17053/2022	Omicron	EPI_ISL_9346874	OM457991
hCoV-19/USA/WA-S17098/2022	Omicron	EPI_ISL_9346918	OM458035
hCoV-19/USA/WA-S17081/2022	Omicron	EPI_ISL_9346901	OM458018
hCoV-19/USA/WA-S17102/2022	Omicron	EPI_ISL_9346922	OM458039
hCoV-19/USA/WA-S17227/2022	Omicron	EPI_ISL_9553012	OM538448
hCoV-19/USA/WA-S17045/2022	Omicron	EPI_ISL_9346866	OM457983
hCoV-19/USA/WA-S17051/2022	Omicron	EPI_ISL_9346872	OM457989
hCoV-19/USA/WA-S17091/2022	Omicron	EPI_ISL_9346911	OM458028
hCoV-19/USA/WA-S17074/2022	Omicron	EPI_ISL_9346894	OM458011
hCoV-19/USA/WA-S17222/2022	Omicron	EPI_ISL_9553007	OM538443
hCoV-19/USA/WA-S17066/2022	Omicron	EPI_ISL_9346886	OM458003
hCoV-19/USA/WA-S17120/2022	Omicron	EPI_ISL_9346940	OM458057
hCoV-19/USA/WA-S17220/2022	Omicron	EPI_ISL_9553005	OM538441
hCoV-19/USA/WA-S17058/2022	Omicron	EPI_ISL_9346879	OM457996
hCoV-19/USA/WA-S17231/2022	Omicron	EPI_ISL_9553016	OM538452
hCoV-19/USA/WA-S17080/2022	Omicron	EPI_ISL_9346900	OM458017
hCoV-19/USA/WA-S17044/2022	Omicron	EPI_ISL_9346865	OM457982
hCoV-19/USA/WA-S17246/2022	Omicron	EPI_ISL_9553031	OM538467
hCoV-19/USA/WA-S17273/2022	Omicron	EPI_ISL_9553058	OM538493
hCoV-19/USA/WA-S17071/2022	Omicron	EPI_ISL_9346891	OM458008
hCoV-19/USA/WA-S17055/2022	Omicron	EPI_ISL_9346876	OM457993
hCoV-19/USA/WA-S17109/2022	Omicron	EPI_ISL_9346929	OM458046
hCoV-19/USA/WA-S17134/2022	Omicron	EPI_ISL_9346954	OM458066
hCoV-19/USA/WA-S17069/2022	Omicron	EPI_ISL_9346889	OM458006

hCoV-19/USA/WA-S17090/2022	Omicron	EPI_ISL_9346910	OM458027
hCoV-19/USA/WA-S17210/2022	Omicron	EPI_ISL_9552995	OM538431
hCoV-19/USA/WA-S17268/2022	Omicron	EPI_ISL_9553053	OM538489
hCoV-19/USA/WA-S17103/2022	Omicron	EPI_ISL_9346923	OM458040
hCoV-19/USA/WA-S17070/2022	Omicron	EPI_ISL_9346890	OM458007
hCoV-19/USA/WA-S17050/2022	Omicron	EPI_ISL_9346871	OM457988
hCoV-19/USA/WA-S17077/2022	Omicron	EPI_ISL_9346897	OM458014
hCoV-19/USA/WA-S17119/2022	Omicron	EPI_ISL_9346939	OM458056
hCoV-19/USA/WA-S17099/2022	Omicron	EPI_ISL_9346919	OM458036
hCoV-19/USA/WA-S17088/2022	Omicron	EPI_ISL_9346908	OM458025
hCoV-19/USA/WA-S17076/2022	Omicron	EPI_ISL_9346896	OM458013
hCoV-19/USA/WA-S17067/2022	Omicron	EPI_ISL_9346887	OM458004
hCoV-19/USA/WA-S17087/2022	Omicron	EPI_ISL_9346907	OM458024
hCoV-19/USA/WA-S17214/2022	Omicron	EPI_ISL_9552999	OM538435
hCoV-19/USA/WA-S17052/2022	Omicron	EPI_ISL_9346873	OM457990
hCoV-19/USA/WA-S17095/2022	Omicron	EPI_ISL_9346915	OM458032
hCoV-19/USA/WA-S17115/2022	Omicron	EPI_ISL_9346935	OM458052
hCoV-19/USA/WA-S17121/2022	Omicron	EPI_ISL_9346941	OM458058
hCoV-19/USA/WA-S16152/2022	Omicron	EPI_ISL_8822471	OM296958
hCoV-19/USA/WA-S17096/2022	Omicron	EPI_ISL_9346916	OM458033
hCoV-19/USA/WA-S17272/2022	Omicron	EPI_ISL_9553057	OM538492
hCoV-19/USA/WA-S17221/2022	Omicron	EPI_ISL_9553006	OM538442
hCoV-19/USA/WA-S17118/2022	Omicron	EPI_ISL_9346938	OM458055
hCoV-19/USA/WA-S17226/2022	Omicron	EPI_ISL_9553011	OM538447
hCoV-19/USA/WA-S17082/2022	Omicron	EPI_ISL_9346902	OM458019
hCoV-19/USA/WA-S17073/2022	Omicron	EPI_ISL_9346893	OM458010
hCoV-19/USA/WA-S17085/2022	Omicron	EPI_ISL_9346905	OM458022
hCoV-19/USA/WA-S17123/2022	Omicron	EPI_ISL_9346943	OM458060
hCoV-19/USA/WA-S17068/2022	Omicron	EPI_ISL_9346888	OM458005
hCoV-19/USA/WA-S17049/2022	Omicron	EPI_ISL_9346870	OM457987
hCoV-19/USA/WA-S17215/2022	Omicron	EPI_ISL_9553000	OM538436
hCoV-19/USA/WA-S17048/2022	Omicron	EPI_ISL_9346869	OM457986
hCoV-19/USA/WA-S17213/2022	Omicron	EPI_ISL_9552998	OM538434
hCoV-19/USA/WA-S17271/2022	Omicron	EPI_ISL_9553056	OM538491
hCoV-19/USA/WA-S17046/2022	Omicron	EPI_ISL_9346867	OM457984
hCoV-19/USA/WA-S17219/2022	Omicron	EPI_ISL_9553004	OM538440
hCoV-19/USA/WA-S17244/2022	Omicron	EPI_ISL_9553029	OM538465
hCoV-19/USA/WA-S17206/2022	Omicron	EPI_ISL_9552991	OM538427
hCoV-19/USA/WA-S17107/2022	Omicron	EPI_ISL_9346927	OM458044
hCoV-19/USA/WA-S17056/2022	Omicron	EPI_ISL_9346877	OM457994
hCoV-19/USA/WA-S17097/2022	Omicron	EPI_ISL_9346917	OM458034
hCoV-19/USA/WA-S17325/2022	Omicron	EPI_ISL_9553110	OM538545
hCoV-19/USA/WA-S17128/2022	Omicron	EPI_ISL_9346948	OM458065
hCoV-19/USA/WA-S17064/2022	Omicron	EPI_ISL_9346884	OM458001
hCoV-19/USA/WA-S16154/2022	Omicron	EPI_ISL_8822473	OM296959
hCoV-19/USA/WA-S17209/2022	Omicron	EPI_ISL_9552994	OM538430
hCoV-19/USA/WA-S17276/2022	Omicron	EPI_ISL_9553061	OM538496
hCoV-19/USA/WA-S17308/2022	Omicron	EPI_ISL_9553093	OM538528
hCoV-19/USA/WA-S17320/2022	Omicron	EPI_ISL_9553105	OM538540
hCoV-19/USA/WA-S17287/2022	Omicron	EPI_ISL_9553072	OM538507
hCoV-19/USA/WA-S17290/2022	Omicron	EPI_ISL_9553075	OM538510

hCoV-19/USA/WA-S17279/2022	Omicron	EPI_ISL_9553064	OM538499
hCoV-19/USA/WA-S17286/2022	Omicron	EPI_ISL_9553071	OM538506
hCoV-19/USA/WA-S17258/2022	Omicron	EPI_ISL_9553043	OM538479
hCoV-19/USA/WA-S17307/2022	Omicron	EPI_ISL_9553092	OM538527
hCoV-19/USA/WA-S17283/2022	Omicron	EPI_ISL_9553068	OM538503
hCoV-19/USA/WA-S17302/2022	Omicron	EPI_ISL_9553087	OM538522
hCoV-19/USA/WA-S17265/2022	Omicron	EPI_ISL_9553050	OM538486
hCoV-19/USA/WA-S17313/2022	Omicron	EPI_ISL_9553098	OM538533
hCoV-19/USA/WA-S17274/2022	Omicron	EPI_ISL_9553059	OM538494
hCoV-19/USA/WA-S17316/2022	Omicron	EPI_ISL_9553101	OM538536
hCoV-19/USA/WA-S17388/2022	Omicron	EPI_ISL_9553173	OM538608
hCoV-19/USA/WA-S17311/2022	Omicron	EPI_ISL_9553096	OM538531
hCoV-19/USA/WA-S17309/2022	Omicron	EPI_ISL_9553094	OM538529
hCoV-19/USA/WA-S17322/2022	Omicron	EPI_ISL_9553107	OM538542
hCoV-19/USA/WA-S17319/2022	Omicron	EPI_ISL_9553104	OM538539
hCoV-19/USA/WA-S17229/2022	Omicron	EPI_ISL_9553014	OM538450
hCoV-19/USA/WA-S17230/2022	Omicron	EPI_ISL_9553015	OM538451
hCoV-19/USA/WA-S17263/2022	Omicron	EPI_ISL_9553048	OM538484
hCoV-19/USA/WA-S17305/2022	Omicron	EPI_ISL_9553090	OM538525
hCoV-19/USA/WA-S17256/2022	Omicron	EPI_ISL_9553041	OM538477
hCoV-19/USA/WA-S17296/2022	Omicron	EPI_ISL_9553081	OM538516
hCoV-19/USA/WA-S17252/2022	Omicron	EPI_ISL_9553037	OM538473
hCoV-19/USA/WA-S17237/2022	Omicron	EPI_ISL_9553022	OM538458
hCoV-19/USA/WA-S17318/2022	Omicron	EPI_ISL_9553103	OM538538
hCoV-19/USA/WA-S17255/2022	Omicron	EPI_ISL_9553040	OM538476
hCoV-19/USA/WA-S17238/2022	Omicron	EPI_ISL_9553023	OM538459
hCoV-19/USA/WA-S17326/2022	Omicron	EPI_ISL_9553111	OM538546
hCoV-19/USA/WA-S17270/2022	Omicron	EPI_ISL_9553055	OM538490
hCoV-19/USA/WA-S17251/2022	Omicron	EPI_ISL_9553036	OM538472
hCoV-19/USA/WA-S17288/2022	Omicron	EPI_ISL_9553073	OM538508
hCoV-19/USA/WA-S17262/2022	Omicron	EPI_ISL_9553047	OM538483
hCoV-19/USA/WA-S17384/2022	Omicron	EPI_ISL_9553169	OM538604
hCoV-19/USA/WA-S17235/2022	Omicron	EPI_ISL_9553020	OM538456
hCoV-19/USA/WA-S17249/2022	Omicron	EPI_ISL_9553034	OM538470
hCoV-19/USA/WA-S17314/2022	Omicron	EPI_ISL_9553099	OM538534
hCoV-19/USA/WA-S17315/2022	Omicron	EPI_ISL_9553100	OM538535
hCoV-19/USA/WA-S17310/2022	Omicron	EPI_ISL_9553095	OM538530
hCoV-19/USA/WA-S17261/2022	Omicron	EPI_ISL_9553046	OM538482
hCoV-19/USA/WA-S17122/2022	Omicron	EPI_ISL_9346942	OM458059
hCoV-19/USA/WA-S17260/2022	Omicron	EPI_ISL_9553045	OM538481
hCoV-19/USA/WA-S17281/2022	Omicron	EPI_ISL_9553066	OM538501
hCoV-19/USA/WA-S17253/2022	Omicron	EPI_ISL_9553038	OM538474
hCoV-19/USA/WA-S17241/2022	Omicron	EPI_ISL_9553026	OM538462
hCoV-19/USA/WA-S17298/2022	Omicron	EPI_ISL_9553083	OM538518
hCoV-19/USA/WA-S17282/2022	Omicron	EPI_ISL_9553067	OM538502
hCoV-19/USA/WA-S17243/2022	Omicron	EPI_ISL_9553028	OM538464
hCoV-19/USA/WA-S17264/2022	Omicron	EPI_ISL_9553049	OM538485
hCoV-19/USA/WA-S17297/2022	Omicron	EPI_ISL_9553082	OM538517
hCoV-19/USA/WA-S17232/2022	Omicron	EPI_ISL_9553017	OM538453
hCoV-19/USA/WA-S17383/2022	Omicron	EPI_ISL_9553168	OM538603
hCoV-19/USA/WA-S17382/2022	Omicron	EPI_ISL_9553167	OM538602

hCoV-19/USA/WA-S17317/2022	Omicron	EPI_ISL_9553102	OM538537
hCoV-19/USA/WA-S17236/2022	Omicron	EPI_ISL_9553021	OM538457
hCoV-19/USA/WA-S17242/2022	Omicron	EPI_ISL_9553027	OM538463
hCoV-19/USA/WA-S17300/2022	Omicron	EPI_ISL_9553085	OM538520
hCoV-19/USA/WA-S17306/2022	Omicron	EPI_ISL_9553091	OM538526
hCoV-19/USA/WA-S17284/2022	Omicron	EPI_ISL_9553069	OM538504
hCoV-19/USA/WA-S17295/2022	Omicron	EPI_ISL_9553080	OM538515
hCoV-19/USA/WA-S17649/2022	Omicron	EPI_ISL_9638266	OM571434
hCoV-19/USA/WA-S17379/2022	Omicron	EPI_ISL_9553164	OM538599
hCoV-19/USA/WA-S17390/2022	Omicron	EPI_ISL_9553175	OM538610
hCoV-19/USA/WA-S17285/2022	Omicron	EPI_ISL_9553070	OM538505
hCoV-19/USA/WA-S17228/2022	Omicron	EPI_ISL_9553013	OM538449
hCoV-19/USA/WA-S17259/2022	Omicron	EPI_ISL_9553044	OM538480
hCoV-19/USA/WA-S17339/2022	Omicron	EPI_ISL_9553124	OM538559
hCoV-19/USA/WA-S17340/2022	Omicron	EPI_ISL_9553125	OM538560
hCoV-19/USA/WA-S17323/2022	Omicron	EPI_ISL_9553108	OM538543
hCoV-19/USA/WA-S17378/2022	Omicron	EPI_ISL_9553163	OM538598
hCoV-19/USA/WA-S17240/2022	Omicron	EPI_ISL_9553025	OM538461
hCoV-19/USA/WA-S17375/2022	Omicron	EPI_ISL_9553160	OM538595
hCoV-19/USA/WA-S17280/2022	Omicron	EPI_ISL_9553065	OM538500
hCoV-19/USA/WA-S17329/2022	Omicron	EPI_ISL_9553114	OM538549
hCoV-19/USA/WA-S17304/2022	Omicron	EPI_ISL_9553089	OM538524
hCoV-19/USA/WA-S17165/2022	Omicron	EPI_ISL_9346986	OM458077
hCoV-19/USA/WA-S17376/2022	Omicron	EPI_ISL_9553161	OM538596
hCoV-19/USA/WA-S17278/2022	Omicron	EPI_ISL_9553063	OM538498
hCoV-19/USA/WA-S17389/2022	Omicron	EPI_ISL_9553174	OM538609
hCoV-19/USA/WA-S17171/2022	Omicron	EPI_ISL_9346992	OM458078
hCoV-19/USA/WA-S17277/2022	Omicron	EPI_ISL_9553062	OM538497
hCoV-19/USA/WA-S17275/2022	Omicron	EPI_ISL_9553060	OM538495
hCoV-19/USA/WA-S17294/2022	Omicron	EPI_ISL_9553079	OM538514
hCoV-19/USA/WA-S17269/2022	Omicron	EPI_ISL_9553054	OP209794
hCoV-19/USA/WA-S17254/2022	Omicron	EPI_ISL_9553039	OM538475
hCoV-19/USA/WA-S17233/2022	Omicron	EPI_ISL_9553018	OM538454
hCoV-19/USA/WA-S17394/2022	Omicron	EPI_ISL_9553179	OM538614
hCoV-19/USA/WA-S17303/2022	Omicron	EPI_ISL_9553088	OM538523
hCoV-19/USA/WA-S17332/2022	Omicron	EPI_ISL_9553117	OM538552
hCoV-19/USA/WA-S17331/2022	Omicron	EPI_ISL_9553116	OM538551
hCoV-19/USA/WA-S17385/2022	Omicron	EPI_ISL_9553170	OM538605
hCoV-19/USA/WA-S17377/2022	Omicron	EPI_ISL_9553162	OM538597
hCoV-19/USA/WA-S17257/2022	Omicron	EPI_ISL_9553042	OM538478
hCoV-19/USA/WA-S17239/2022	Omicron	EPI_ISL_9553024	OM538460
hCoV-19/USA/WA-S17381/2022	Omicron	EPI_ISL_9553166	OM538601
hCoV-19/USA/WA-S17234/2022	Omicron	EPI_ISL_9553019	OM538455
hCoV-19/USA/WA-S17374/2022	Omicron	EPI_ISL_9553159	OM538594
hCoV-19/USA/WA-S17380/2022	Omicron	EPI_ISL_9553165	OM538600
hCoV-19/USA/WA-S17291/2022	Omicron	EPI_ISL_9553076	OM538511
hCoV-19/USA/WA-S17301/2022	Omicron	EPI_ISL_9553086	OM538521
hCoV-19/USA/WA-S17321/2022	Omicron	EPI_ISL_9553106	OM538541
hCoV-19/USA/WA-S17312/2022	Omicron	EPI_ISL_9553097	OM538532
hCoV-19/USA/WA-S17386/2022	Omicron	EPI_ISL_9553171	OM538606
hCoV-19/USA/WA-S17387/2022	Omicron	EPI_ISL_9553172	OM538607

hCoV-19/USA/WA-S17430/2022	Omicron	EPI_ISL_9553213	OM538648
hCoV-19/USA/WA-S17403/2022	Omicron	EPI_ISL_9553188	OM538623
hCoV-19/USA/WA-S17351/2022	Omicron	EPI_ISL_9553136	OM538571
hCoV-19/USA/WA-S17367/2022	Omicron	EPI_ISL_9553152	OM538587
hCoV-19/USA/WA-S17398/2022	Omicron	EPI_ISL_9553183	OM538618
hCoV-19/USA/WA-S17425/2022	Omicron	EPI_ISL_9553208	OM538643
hCoV-19/USA/WA-S17431/2022	Omicron	EPI_ISL_9553214	OM538649
hCoV-19/USA/WA-S17350/2022	Omicron	EPI_ISL_9553135	OM538570
hCoV-19/USA/WA-S17568/2022	Omicron	EPI_ISL_9638186	OM571354
hCoV-19/USA/WA-S17373/2022	Omicron	EPI_ISL_9553158	OM538593
hCoV-19/USA/WA-S17391/2022	Omicron	EPI_ISL_9553176	OM538611
hCoV-19/USA/WA-S17413/2022	Omicron	EPI_ISL_9553197	OM538632
hCoV-19/USA/WA-S17328/2022	Omicron	EPI_ISL_9553113	OM538548
hCoV-19/USA/WA-S17406/2022	Omicron	EPI_ISL_9553190	OM538625
hCoV-19/USA/WA-S17422/2022	Omicron	EPI_ISL_9553206	OM538641
hCoV-19/USA/WA-S17372/2022	Omicron	EPI_ISL_9553157	OM538592
hCoV-19/USA/WA-S17371/2022	Omicron	EPI_ISL_9553156	OM538591
hCoV-19/USA/WA-S17361/2022	Omicron	EPI_ISL_9553146	OM538581
hCoV-19/USA/WA-S17342/2022	Omicron	EPI_ISL_9553127	OM538562
hCoV-19/USA/WA-S17410/2022	Omicron	EPI_ISL_9553194	OM538629
hCoV-19/USA/WA-S17359/2022	Omicron	EPI_ISL_9553144	OM538579
hCoV-19/USA/WA-S17338/2022	Omicron	EPI_ISL_9553123	OM538558
hCoV-19/USA/WA-S17408/2022	Omicron	EPI_ISL_9553192	OM538627
hCoV-19/USA/WA-S17421/2022	Omicron	EPI_ISL_9553205	OM538640
hCoV-19/USA/WA-S17566/2022	Omicron	EPI_ISL_9638184	OM571352
hCoV-19/USA/WA-S17401/2022	Omicron	EPI_ISL_9553186	OM538621
hCoV-19/USA/WA-S17435/2022	Omicron	EPI_ISL_9553218	OM538653
hCoV-19/USA/WA-S17333/2022	Omicron	EPI_ISL_9553118	OM538553
hCoV-19/USA/WA-S17365/2022	Omicron	EPI_ISL_9553150	OM538585
hCoV-19/USA/WA-S17567/2022	Omicron	EPI_ISL_9638185	OM571353
hCoV-19/USA/WA-S17412/2022	Omicron	EPI_ISL_9553196	OM538631
hCoV-19/USA/WA-S17392/2022	Omicron	EPI_ISL_9553177	OM538612
hCoV-19/USA/WA-S17432/2022	Omicron	EPI_ISL_9553215	OM538650
hCoV-19/USA/WA-S17405/2022	Omicron	EPI_ISL_9553189	OM538624
hCoV-19/USA/WA-S17354/2022	Omicron	EPI_ISL_9553139	OM538574
hCoV-19/USA/WA-S17362/2022	Omicron	EPI_ISL_9553147	OM538582
hCoV-19/USA/WA-S17416/2022	Omicron	EPI_ISL_9553200	OM538635
hCoV-19/USA/WA-S17399/2022	Omicron	EPI_ISL_9553184	OM538619
hCoV-19/USA/WA-S17174/2022	Omicron	EPI_ISL_9346995	OM458079
hCoV-19/USA/WA-S17352/2022	Omicron	EPI_ISL_9553137	OM538572
hCoV-19/USA/WA-S17330/2022	Omicron	EPI_ISL_9553115	OM538550
hCoV-19/USA/WA-S17433/2022	Omicron	EPI_ISL_9553216	OM538651
hCoV-19/USA/WA-S17427/2022	Omicron	EPI_ISL_9553210	OM538645
hCoV-19/USA/WA-S17341/2022	Omicron	EPI_ISL_9553126	OM538561
hCoV-19/USA/WA-S17400/2022	Omicron	EPI_ISL_9553185	OM538620
hCoV-19/USA/WA-S17549/2022	Omicron	EPI_ISL_9638167	OM571335
hCoV-19/USA/WA-S17569/2022	Omicron	EPI_ISL_9638187	OM571355
hCoV-19/USA/WA-S17553/2022	Omicron	EPI_ISL_9638171	OM571339
hCoV-19/USA/WA-S17417/2022	Omicron	EPI_ISL_9553201	OM538636
hCoV-19/USA/WA-S17368/2022	Omicron	EPI_ISL_9553153	OM538588
hCoV-19/USA/WA-S17419/2022	Omicron	EPI_ISL_9553203	OM538638

hCoV-19/USA/WA-S17334/2022	Omicron	EPI_ISL_9553119	OM538554
hCoV-19/USA/WA-S17434/2022	Omicron	EPI_ISL_9553217	OM538652
hCoV-19/USA/WA-S17370/2022	Omicron	EPI_ISL_9553155	OM538590
hCoV-19/USA/WA-S17353/2022	Omicron	EPI_ISL_9553138	OM538573
hCoV-19/USA/WA-S17544/2022	Omicron	EPI_ISL_9638162	OM571330
hCoV-19/USA/WA-S17355/2022	Omicron	EPI_ISL_9553140	OM538575
hCoV-19/USA/WA-S17428/2022	Omicron	EPI_ISL_9553211	OM538646
hCoV-19/USA/WA-S17356/2022	Omicron	EPI_ISL_9553141	OM538576
hCoV-19/USA/WA-S17347/2022	Omicron	EPI_ISL_9553132	OM538567
hCoV-19/USA/WA-S17327/2022	Omicron	EPI_ISL_9553112	OM538547
hCoV-19/USA/WA-S17564/2022	Omicron	EPI_ISL_9638182	OM571350
hCoV-19/USA/WA-S17348/2022	Omicron	EPI_ISL_9553133	OM538568
hCoV-19/USA/WA-S17409/2022	Omicron	EPI_ISL_9553193	OM538628
hCoV-19/USA/WA-S17364/2022	Omicron	EPI_ISL_9553149	OM538584
hCoV-19/USA/WA-S17565/2022	Omicron	EPI_ISL_9638183	OM571351
hCoV-19/USA/WA-S17415/2022	Omicron	EPI_ISL_9553199	OM538634
hCoV-19/USA/WA-S17358/2022	Omicron	EPI_ISL_9553143	OM538578
hCoV-19/USA/WA-S17426/2022	Omicron	EPI_ISL_9553209	OM538644
hCoV-19/USA/WA-S17420/2022	Omicron	EPI_ISL_9553204	OM538639
hCoV-19/USA/WA-S17550/2022	Omicron	EPI_ISL_9638168	OM571336
hCoV-19/USA/WA-S17414/2022	Omicron	EPI_ISL_9553198	OM538633
hCoV-19/USA/WA-S17570/2022	Omicron	EPI_ISL_9638188	OM571356
hCoV-19/USA/WA-S17366/2022	Omicron	EPI_ISL_9553151	OM538586
hCoV-19/USA/WA-S17411/2022	Omicron	EPI_ISL_9553195	OM538630
hCoV-19/USA/WA-S17436/2022	Omicron	EPI_ISL_9553219	OM538654
hCoV-19/USA/WA-S17418/2022	Omicron	EPI_ISL_9553202	OM538637
hCoV-19/USA/WA-S17397/2022	Omicron	EPI_ISL_9553182	OM538617
hCoV-19/USA/WA-S17336/2022	Omicron	EPI_ISL_9553121	OM538556
hCoV-19/USA/WA-S17552/2022	Omicron	EPI_ISL_9638170	OM571338
hCoV-19/USA/WA-S17335/2022	Omicron	EPI_ISL_9553120	OM538555
hCoV-19/USA/WA-S17360/2022	Omicron	EPI_ISL_9553145	OM538580
hCoV-19/USA/WA-S17402/2022	Omicron	EPI_ISL_9553187	OM538622
hCoV-19/USA/WA-S17369/2022	Omicron	EPI_ISL_9553154	OM538589
hCoV-19/USA/WA-S17343/2022	Omicron	EPI_ISL_9553128	OM538563
hCoV-19/USA/WA-S17337/2022	Omicron	EPI_ISL_9553122	OM538557
hCoV-19/USA/WA-S17548/2022	Omicron	EPI_ISL_9638166	OM571334
hCoV-19/USA/WA-S17344/2022	Omicron	EPI_ISL_9553129	OM538564
hCoV-19/USA/WA-S17396/2022	Omicron	EPI_ISL_9553181	OM538616
hCoV-19/USA/WA-S17407/2022	Omicron	EPI_ISL_9553191	OM538626
hCoV-19/USA/WA-S17625/2022	Omicron	EPI_ISL_9638242	OM571410
hCoV-19/USA/WA-S17546/2022	Omicron	EPI_ISL_9638164	OM571332
hCoV-19/USA/WA-S17429/2022	Omicron	EPI_ISL_9553212	OM538647
hCoV-19/USA/WA-S17604/2022	Omicron	EPI_ISL_9638221	OM571389
hCoV-19/USA/WA-S17598/2022	Omicron	EPI_ISL_9638215	OM571383
hCoV-19/USA/WA-S17639/2022	Omicron	EPI_ISL_9638256	OM571424
hCoV-19/USA/WA-S17542/2022	Omicron	EPI_ISL_9638160	OM571328
hCoV-19/USA/WA-S17578/2022	Omicron	EPI_ISL_9638196	OM571364
hCoV-19/USA/WA-S17587/2022	Omicron	EPI_ISL_9638205	OM571373
hCoV-19/USA/WA-S17543/2022	Omicron	EPI_ISL_9638161	OM571329
hCoV-19/USA/WA-S17581/2022	Omicron	EPI_ISL_9638199	OM571367
hCoV-19/USA/WA-S17572/2022	Omicron	EPI_ISL_9638190	OM571358

hCoV-19/USA/WA-S17575/2022	Omicron	EPI_ISL_9638193	OM571361
hCoV-19/USA/WA-S17437/2022	Omicron	EPI_ISL_9553220	OM538655
hCoV-19/USA/WA-S17545/2022	Omicron	EPI_ISL_9638163	OM571331
hCoV-19/USA/WA-S17440/2022	Omicron	EPI_ISL_9553223	OM538658
hCoV-19/USA/WA-S17603/2022	Omicron	EPI_ISL_9638220	OM571388
hCoV-19/USA/WA-S17541/2022	Omicron	EPI_ISL_9638159	OM571327
hCoV-19/USA/WA-S17641/2022	Omicron	EPI_ISL_9638258	OM571426
hCoV-19/USA/WA-S17599/2022	Omicron	EPI_ISL_9638216	OM571384
hCoV-19/USA/WA-S17438/2022	Omicron	EPI_ISL_9553221	OM538656
hCoV-19/USA/WA-S17605/2022	Omicron	EPI_ISL_9638222	OM571390
hCoV-19/USA/WA-S17576/2022	Omicron	EPI_ISL_9638194	OM571362
hCoV-19/USA/WA-S17595/2022	Omicron	EPI_ISL_9638212	OM571380
hCoV-19/USA/WA-S17596/2022	Omicron	EPI_ISL_9638213	OM571381
hCoV-19/USA/WA-S17589/2022	Omicron	EPI_ISL_9638207	OM571375
hCoV-19/USA/WA-S17554/2022	Omicron	EPI_ISL_9638172	OM571340
hCoV-19/USA/WA-S17610/2022	Omicron	EPI_ISL_9638227	OM571395
hCoV-19/USA/WA-S17579/2022	Omicron	EPI_ISL_9638197	OM571365
hCoV-19/USA/WA-S17600/2022	Omicron	EPI_ISL_9638217	OM571385
hCoV-19/USA/WA-S17561/2022	Omicron	EPI_ISL_9638179	OM571347
hCoV-19/USA/WA-S17540/2022	Omicron	EPI_ISL_9638158	OM571326
hCoV-19/USA/WA-S17638/2022	Omicron	EPI_ISL_9638255	OM571423
hCoV-19/USA/WA-S17590/2022	Omicron	EPI_ISL_9638208	OM571376
hCoV-19/USA/WA-S17580/2022	Omicron	EPI_ISL_9638198	OM571366
hCoV-19/USA/WA-S17445/2022	Omicron	EPI_ISL_9553228	OM538663
hCoV-19/USA/WA-S17559/2022	Omicron	EPI_ISL_9638177	OM571345
hCoV-19/USA/WA-S17586/2022	Omicron	EPI_ISL_9638204	OM571372
hCoV-19/USA/WA-S17577/2022	Omicron	EPI_ISL_9638195	OM571363
hCoV-19/USA/WA-S17573/2022	Omicron	EPI_ISL_9638191	OM571359
hCoV-19/USA/WA-S17652/2022	Omicron	EPI_ISL_9638269	OM571437
hCoV-19/USA/WA-S17562/2022	Omicron	EPI_ISL_9638180	OM571348
hCoV-19/USA/WA-S17601/2022	Omicron	EPI_ISL_9638218	OM571386
hCoV-19/USA/WA-S17447/2022	Omicron	EPI_ISL_9553230	OM538664
hCoV-19/USA/WA-S17651/2022	Omicron	EPI_ISL_9638268	OM571436
hCoV-19/USA/WA-S17441/2022	Omicron	EPI_ISL_9553224	OM538659
hCoV-19/USA/WA-S17640/2022	Omicron	EPI_ISL_9638257	OM571425
hCoV-19/USA/WA-S17444/2022	Omicron	EPI_ISL_9553227	OM538662
hCoV-19/USA/WA-S17660/2022	Omicron	EPI_ISL_9638277	OM571445
hCoV-19/USA/WA-S17643/2022	Omicron	EPI_ISL_9638260	OM571428
hCoV-19/USA/WA-S17654/2022	Omicron	EPI_ISL_9638271	OM571439
hCoV-19/USA/WA-S17439/2022	Omicron	EPI_ISL_9553222	OM538657
hCoV-19/USA/WA-S17695/2022	Omicron	EPI_ISL_9638312	OM571478
hCoV-19/USA/WA-S17443/2022	Omicron	EPI_ISL_9553226	OM538661
hCoV-19/USA/WA-S17442/2022	Omicron	EPI_ISL_9553225	OM538660
hCoV-19/USA/WA-S17608/2022	Omicron	EPI_ISL_9638225	OM571393
hCoV-19/USA/WA-S17623/2022	Omicron	EPI_ISL_9638240	OM571408
hCoV-19/USA/WA-S17642/2022	Omicron	EPI_ISL_9638259	OM571427
hCoV-19/USA/WA-S17571/2022	Omicron	EPI_ISL_9638189	OM571357
hCoV-19/USA/WA-S17606/2022	Omicron	EPI_ISL_9638223	OM571391
hCoV-19/USA/WA-S17585/2022	Omicron	EPI_ISL_9638203	OM571371
hCoV-19/USA/WA-S17582/2022	Omicron	EPI_ISL_9638200	OM571368
hCoV-19/USA/WA-S17594/2022	Omicron	EPI_ISL_9638211	OM571379

hCoV-19/USA/WA-S17644/2022	Omicron	EPI_ISL_9638261	OM571429
hCoV-19/USA/WA-S17646/2022	Omicron	EPI_ISL_9638263	OM571431
hCoV-19/USA/WA-S17645/2022	Omicron	EPI_ISL_9638262	OM571430
hCoV-19/USA/WA-S17555/2022	Omicron	EPI_ISL_9638173	OM571341
hCoV-19/USA/WA-S17455/2022	Omicron	EPI_ISL_9553238	OM538665
hCoV-19/USA/WA-S17557/2022	Omicron	EPI_ISL_9638175	OM571343
hCoV-19/USA/WA-S17556/2022	Omicron	EPI_ISL_9638174	OM571342
hCoV-19/USA/WA-S17597/2022	Omicron	EPI_ISL_9638214	OM571382
hCoV-19/USA/WA-S17547/2022	Omicron	EPI_ISL_9638165	OM571333
hCoV-19/USA/WA-S17539/2022	Omicron	EPI_ISL_9638157	OM571325
hCoV-19/USA/WA-S17558/2022	Omicron	EPI_ISL_9638176	OM571344
hCoV-19/USA/WA-S17583/2022	Omicron	EPI_ISL_9638201	OM571369
hCoV-19/USA/WA-S17574/2022	Omicron	EPI_ISL_9638192	OM571360
hCoV-19/USA/WA-S17591/2022	Omicron	EPI_ISL_9638209	OM571377
hCoV-19/USA/WA-S17650/2022	Omicron	EPI_ISL_9638267	OM571435
hCoV-19/USA/WA-S17656/2022	Omicron	EPI_ISL_9638273	OM571441
hCoV-19/USA/WA-S17648/2022	Omicron	EPI_ISL_9638265	OM571433
hCoV-19/USA/WA-S17653/2022	Omicron	EPI_ISL_9638270	OM571438
hCoV-19/USA/WA-S17630/2022	Omicron	EPI_ISL_9638247	OM571415
hCoV-19/USA/WA-S17681/2022	Omicron	EPI_ISL_9638298	OM571464
hCoV-19/USA/WA-S17624/2022	Omicron	EPI_ISL_9638241	OM571409
hCoV-19/USA/WA-S17724/2022	Omicron	EPI_ISL_9638341	OM571507
hCoV-19/USA/WA-S17658/2022	Omicron	EPI_ISL_9638275	OM571443
hCoV-19/USA/WA-S17628/2022	Omicron	EPI_ISL_9638245	OM571413
hCoV-19/USA/WA-S17672/2022	Omicron	EPI_ISL_9638289	OM571455
hCoV-19/USA/WA-S17680/2022	Omicron	EPI_ISL_9638297	OM571463
hCoV-19/USA/WA-S17635/2022	Omicron	EPI_ISL_9638252	OM571420
hCoV-19/USA/WA-S17664/2022	Omicron	EPI_ISL_9638281	OP209796
hCoV-19/USA/WA-S17674/2022	Omicron	EPI_ISL_9638291	OM571457
hCoV-19/USA/WA-S17663/2022	Omicron	EPI_ISL_9638280	OM571448
hCoV-19/USA/WA-S17723/2022	Omicron	EPI_ISL_9638340	OM571506
hCoV-19/USA/WA-S17668/2022	Omicron	EPI_ISL_9638285	OP209797
hCoV-19/USA/WA-S17683/2022	Omicron	EPI_ISL_9638300	OM571466
hCoV-19/USA/WA-S17673/2022	Omicron	EPI_ISL_9638290	OM571456
hCoV-19/USA/WA-S17685/2022	Omicron	EPI_ISL_9638302	OM571468
hCoV-19/USA/WA-S17667/2022	Omicron	EPI_ISL_9638284	OM571451
hCoV-19/USA/WA-S17694/2022	Omicron	EPI_ISL_9638311	OM571477
hCoV-19/USA/WA-S17657/2022	Omicron	EPI_ISL_9638274	OM571442
hCoV-19/USA/WA-S17633/2022	Omicron	EPI_ISL_9638250	OM571418
hCoV-19/USA/WA-S17686/2022	Omicron	EPI_ISL_9638303	OM571469
hCoV-19/USA/WA-S17631/2022	Omicron	EPI_ISL_9638248	OM571416
hCoV-19/USA/WA-S17647/2022	Omicron	EPI_ISL_9638264	OM571432
hCoV-19/USA/WA-S17616/2022	Omicron	EPI_ISL_9638233	OM571401
hCoV-19/USA/WA-S17679/2022	Omicron	EPI_ISL_9638296	OM571462
hCoV-19/USA/WA-S17617/2022	Omicron	EPI_ISL_9638234	OM571402
hCoV-19/USA/WA-S17701/2022	Omicron	EPI_ISL_9638318	OM571484
hCoV-19/USA/WA-S17662/2022	Omicron	EPI_ISL_9638279	OM571447
hCoV-19/USA/WA-S17659/2022	Omicron	EPI_ISL_9638276	OM571444
hCoV-19/USA/WA-S17666/2022	Omicron	EPI_ISL_9638283	OM571450
hCoV-19/USA/WA-S17614/2022	Omicron	EPI_ISL_9638231	OM571399
hCoV-19/USA/WA-S17670/2022	Omicron	EPI_ISL_9638287	OM571453

hCoV-19/USA/WA-S17697/2022	Omicron	EPI_ISL_9638314	OM571480
hCoV-19/USA/WA-S17619/2022	Omicron	EPI_ISL_9638236	OM571404
hCoV-19/USA/WA-S17698/2022	Omicron	EPI_ISL_9638315	OM571481
hCoV-19/USA/WA-S17626/2022	Omicron	EPI_ISL_9638243	OM571411
hCoV-19/USA/WA-S17627/2022	Omicron	EPI_ISL_9638244	OM571412
hCoV-19/USA/WA-S17629/2022	Omicron	EPI_ISL_9638246	OM571414
hCoV-19/USA/WA-S17678/2022	Omicron	EPI_ISL_9638295	OM571461
hCoV-19/USA/WA-S17613/2022	Omicron	EPI_ISL_9638230	OM571398
hCoV-19/USA/WA-S17615/2022	Omicron	EPI_ISL_9638232	OM571400
hCoV-19/USA/WA-S17661/2022	Omicron	EPI_ISL_9638278	OM571446
hCoV-19/USA/WA-S17693/2022	Omicron	EPI_ISL_9638310	OM571476
hCoV-19/USA/WA-S17612/2022	Omicron	EPI_ISL_9638229	OM571397
hCoV-19/USA/WA-S17691/2022	Omicron	EPI_ISL_9638308	OM571474
hCoV-19/USA/WA-S17684/2022	Omicron	EPI_ISL_9638301	OM571467
hCoV-19/USA/WA-S17699/2022	Omicron	EPI_ISL_9638316	OM571482
hCoV-19/USA/WA-S17609/2022	Omicron	EPI_ISL_9638226	OM571394
hCoV-19/USA/WA-S17637/2022	Omicron	EPI_ISL_9638254	OM571422
hCoV-19/USA/WA-S17689/2022	Omicron	EPI_ISL_9638306	OM571472
hCoV-19/USA/WA-S17700/2022	Omicron	EPI_ISL_9638317	OM571483
hCoV-19/USA/WA-S17720/2022	Omicron	EPI_ISL_9638337	OM571503
hCoV-19/USA/WA-S17634/2022	Omicron	EPI_ISL_9638251	OM571419
hCoV-19/USA/WA-S17696/2022	Omicron	EPI_ISL_9638313	OM571479
hCoV-19/USA/WA-S17636/2022	Omicron	EPI_ISL_9638253	OM571421
hCoV-19/USA/WA-S17632/2022	Omicron	EPI_ISL_9638249	OM571417
hCoV-19/USA/WA-S17682/2022	Omicron	EPI_ISL_9638299	OM571465
hCoV-19/USA/WA-S17677/2022	Omicron	EPI_ISL_9638294	OM571460
hCoV-19/USA/WA-S17671/2022	Omicron	EPI_ISL_9638288	OM571454
hCoV-19/USA/WA-S17611/2022	Omicron	EPI_ISL_9638228	OM571396
hCoV-19/USA/WA-S17687/2022	Omicron	EPI_ISL_9638304	OM571470
hCoV-19/USA/WA-S17702/2022	Omicron	EPI_ISL_9638319	OM571485
hCoV-19/USA/WA-S17704/2022	Omicron	EPI_ISL_9638321	OM571487
hCoV-19/USA/WA-S17726/2022	Omicron	EPI_ISL_9638343	OM571509
hCoV-19/USA/WA-S17716/2022	Omicron	EPI_ISL_9638333	OM571499
hCoV-19/USA/WA-S17722/2022	Omicron	EPI_ISL_9638339	OM571505
hCoV-19/USA/WA-S17713/2022	Omicron	EPI_ISL_9638330	OM571496
hCoV-19/USA/WA-S17712/2022	Omicron	EPI_ISL_9638329	OM571495
hCoV-19/USA/WA-S17727/2022	Omicron	EPI_ISL_9638344	OM571510
hCoV-19/USA/WA-S17714/2022	Omicron	EPI_ISL_9638331	OM571497
hCoV-19/USA/WA-S17688/2022	Omicron	EPI_ISL_9638305	OM571471
hCoV-19/USA/WA-S17710/2022	Omicron	EPI_ISL_9638327	OM571493
hCoV-19/USA/WA-S17705/2022	Omicron	EPI_ISL_9638322	OM571488
hCoV-19/USA/WA-S17715/2022	Omicron	EPI_ISL_9638332	OM571498
hCoV-19/USA/WA-S17718/2022	Omicron	EPI_ISL_9638335	OM571501
hCoV-19/USA/WA-S17708/2022	Omicron	EPI_ISL_9638325	OM571491
hCoV-19/USA/WA-S17731/2022	Omicron	EPI_ISL_9638348	OM571514
hCoV-19/USA/WA-S17703/2022	Omicron	EPI_ISL_9638320	OM571486
hCoV-19/USA/WA-S17706/2022	Omicron	EPI_ISL_9638323	OM571489
hCoV-19/USA/WA-S17707/2022	Omicron	EPI_ISL_9638324	OM571490
hCoV-19/USA/WA-S17733/2022	Omicron	EPI_ISL_9638350	OM571516
hCoV-19/USA/WA-S17709/2022	Omicron	EPI_ISL_9638326	OM571492
hCoV-19/USA/WA-S17719/2022	Omicron	EPI_ISL_9638336	OM571502

hCoV-19/USA/WA-S17711/2022	Omicron	EPI_ISL_9638328	OM571494
hCoV-19/USA/WA-S17717/2022	Omicron	EPI_ISL_9638334	OM571500
hCoV-19/USA/WA-S17721/2022	Omicron	EPI_ISL_9638338	OM571504
hCoV-19/USA/WA-S17730/2022	Omicron	EPI_ISL_9638347	OM571513
hCoV-19/USA/WA-S17725/2022	Omicron	EPI_ISL_9638342	OM571508
hCoV-19/USA/WA-S17732/2022	Omicron	EPI_ISL_9638349	OM571515
hCoV-19/USA/WA-S17728/2022	Omicron	EPI_ISL_9638345	OM571511
hCoV-19/USA/WA-S17746/2022	Omicron	EPI_ISL_9638363	OM571529
hCoV-19/USA/WA-S17754/2022	Omicron	EPI_ISL_9638371	OM571537
hCoV-19/USA/WA-S17753/2022	Omicron	EPI_ISL_9638370	OM571536
hCoV-19/USA/WA-S17741/2022	Omicron	EPI_ISL_9638358	OM571524
hCoV-19/USA/WA-S17738/2022	Omicron	EPI_ISL_9638355	OM571521
hCoV-19/USA/WA-S17737/2022	Omicron	EPI_ISL_9638354	OM571520
hCoV-19/USA/WA-S17752/2022	Omicron	EPI_ISL_9638369	OM571535
hCoV-19/USA/WA-S17750/2022	Omicron	EPI_ISL_9638367	OM571533
hCoV-19/USA/WA-S17945/2022	Omicron	EPI_ISL_9881812	OM676013
hCoV-19/USA/WA-S17744/2022	Omicron	EPI_ISL_9638361	OM571527
hCoV-19/USA/WA-S17743/2022	Omicron	EPI_ISL_9638360	OM571526
hCoV-19/USA/WA-S17736/2022	Omicron	EPI_ISL_9638353	OM571519
hCoV-19/USA/WA-S17739/2022	Omicron	EPI_ISL_9638356	OM571522
hCoV-19/USA/WA-S17747/2022	Omicron	EPI_ISL_9638364	OM571530
hCoV-19/USA/WA-S17734/2022	Omicron	EPI_ISL_9638351	OM571517
hCoV-19/USA/WA-S17748/2022	Omicron	EPI_ISL_9638365	OM571531
hCoV-19/USA/WA-S17740/2022	Omicron	EPI_ISL_9638357	OM571523
hCoV-19/USA/WA-S17946/2022	Omicron	EPI_ISL_9881758	OM676014
hCoV-19/USA/WA-S17774/2022	Omicron	EPI_ISL_9638391	OM571557
hCoV-19/USA/WA-S17938/2022	Omicron	EPI_ISL_9881535	OM676006
hCoV-19/USA/WA-S17882/2022	Omicron	EPI_ISL_9881491	OM675989
hCoV-19/USA/WA-S17772/2022	Omicron	EPI_ISL_9638389	OM571555
hCoV-19/USA/WA-S17939/2022	Omicron	EPI_ISL_9881536	OM676007
hCoV-19/USA/WA-S17775/2022	Omicron	EPI_ISL_9638392	OM571558
hCoV-19/USA/WA-S17895/2022	Omicron	EPI_ISL_9881500	OM675992
hCoV-19/USA/WA-S17759/2022	Omicron	EPI_ISL_9638376	OM571542
hCoV-19/USA/WA-S17937/2022	Omicron	EPI_ISL_9881534	OM676005
hCoV-19/USA/WA-S17768/2022	Omicron	EPI_ISL_9638385	OM571551
hCoV-19/USA/WA-S17778/2022	Omicron	EPI_ISL_9638395	OM571561
hCoV-19/USA/WA-S17771/2022	Omicron	EPI_ISL_9638388	OM571554
hCoV-19/USA/WA-S17942/2022	Omicron	EPI_ISL_9881539	OM676010
hCoV-19/USA/WA-S17943/2022	Omicron	EPI_ISL_9881799	OM676011
hCoV-19/USA/WA-S17884/2022	Omicron	EPI_ISL_9881472	OM675991
hCoV-19/USA/WA-S17782/2022	Omicron	EPI_ISL_9638399	OM571565
hCoV-19/USA/WA-S17767/2022	Omicron	EPI_ISL_9638384	OM571550
hCoV-19/USA/WA-S17763/2022	Omicron	EPI_ISL_9638380	OM571546
hCoV-19/USA/WA-S17879/2022	Omicron	EPI_ISL_9881813	OM675986
hCoV-19/USA/WA-S17948/2022	Omicron	EPI_ISL_9881541	OM676016
hCoV-19/USA/WA-S17761/2022	Omicron	EPI_ISL_9638378	OM571544
hCoV-19/USA/WA-S17936/2022	Omicron	EPI_ISL_9881796	OM676004
hCoV-19/USA/WA-S17770/2022	Omicron	EPI_ISL_9638387	OM571553
hCoV-19/USA/WA-S17766/2022	Omicron	EPI_ISL_9638383	OM571549
hCoV-19/USA/WA-S17758/2022	Omicron	EPI_ISL_9638375	OM571541
hCoV-19/USA/WA-S17776/2022	Omicron	EPI_ISL_9638393	OM571559

hCoV-19/USA/WA-S17757/2022	Omicron	EPI_ISL_9638374	OM571540
hCoV-19/USA/WA-S17880/2022	Omicron	EPI_ISL_9881778	OM675987
hCoV-19/USA/WA-S17780/2022	Omicron	EPI_ISL_9638397	OM571563
hCoV-19/USA/WA-S17755/2022	Omicron	EPI_ISL_9638372	OM571538
hCoV-19/USA/WA-S17944/2022	Omicron	EPI_ISL_9881789	OM676012
hCoV-19/USA/WA-S17947/2022	Omicron	EPI_ISL_9881540	OM676015
hCoV-19/USA/WA-S17777/2022	Omicron	EPI_ISL_9638394	OM571560
hCoV-19/USA/WA-S18002/2022	Omicron	EPI_ISL_9881583	OM676052
hCoV-19/USA/WA-S17760/2022	Omicron	EPI_ISL_9638377	OM571543
hCoV-19/USA/WA-S17751/2022	Omicron	EPI_ISL_9638368	OM571534
hCoV-19/USA/WA-S17762/2022	Omicron	EPI_ISL_9638379	OM571545
hCoV-19/USA/WA-S17781/2022	Omicron	EPI_ISL_9638398	OM571564
hCoV-19/USA/WA-S17765/2022	Omicron	EPI_ISL_9638382	OM571548
hCoV-19/USA/WA-S18026/2022	Omicron	EPI_ISL_9881605	OM676058
hCoV-19/USA/WA-S17969/2022	Omicron	EPI_ISL_9881811	OM676025
hCoV-19/USA/WA-S17985/2022	Omicron	EPI_ISL_9881569	OM676041
hCoV-19/USA/WA-S17933/2022	Omicron	EPI_ISL_9881532	OM676001
hCoV-19/USA/WA-S17994/2022	Omicron	EPI_ISL_9881575	OM676050
hCoV-19/USA/WA-S17974/2022	Omicron	EPI_ISL_9881562	OM676030
hCoV-19/USA/WA-S17965/2022	Omicron	EPI_ISL_9881558	OM676021
hCoV-19/USA/WA-S17967/2022	Omicron	EPI_ISL_9881797	OM676023
hCoV-19/USA/WA-S17984/2022	Omicron	EPI_ISL_9881568	OM676040
hCoV-19/USA/WA-S18054/2022	Omicron	EPI_ISL_9881800	OM676086
hCoV-19/USA/WA-S17989/2022	Omicron	EPI_ISL_9881573	OM676045
hCoV-19/USA/WA-S18056/2022	Omicron	EPI_ISL_9881625	OM676088
hCoV-19/USA/WA-S17980/2022	Omicron	EPI_ISL_9881567	OM676036
hCoV-19/USA/WA-S17935/2022	Omicron	EPI_ISL_9881762	OM676003
hCoV-19/USA/WA-S17977/2022	Omicron	EPI_ISL_9881564	OM676033
hCoV-19/USA/WA-S17941/2022	Omicron	EPI_ISL_9881538	OM676009
hCoV-19/USA/WA-S17990/2022	Omicron	EPI_ISL_9881763	OM676046
hCoV-19/USA/WA-S17979/2022	Omicron	EPI_ISL_9881566	OM676035
hCoV-19/USA/WA-S17940/2022	Omicron	EPI_ISL_9881537	OM676008
hCoV-19/USA/WA-S17975/2022	Omicron	EPI_ISL_9881803	OM676031
hCoV-19/USA/WA-S17976/2022	Omicron	EPI_ISL_9881563	OM676032
hCoV-19/USA/WA-S18025/2022	Omicron	EPI_ISL_9881604	OM676057
hCoV-19/USA/WA-S17928/2022	Omicron	EPI_ISL_9881783	OM675997
hCoV-19/USA/WA-S17929/2022	Omicron	EPI_ISL_9881529	OM675998
hCoV-19/USA/WA-S17987/2022	Omicron	EPI_ISL_9881571	OM676043
hCoV-19/USA/WA-S17926/2022	Omicron	EPI_ISL_9881527	OM675995
hCoV-19/USA/WA-S17982/2022	Omicron	EPI_ISL_9881473	OM676038
hCoV-19/USA/WA-S17964/2022	Omicron	EPI_ISL_9881557	OM676020
hCoV-19/USA/WA-S17925/2022	Omicron	EPI_ISL_9881526	OM675994
hCoV-19/USA/WA-S17981/2022	Omicron	EPI_ISL_9881810	OM676037
hCoV-19/USA/WA-S17971/2022	Omicron	EPI_ISL_9881560	OM676027
hCoV-19/USA/WA-S18053/2022	Omicron	EPI_ISL_9881623	OM676085
hCoV-19/USA/WA-S18049/2022	Omicron	EPI_ISL_9881620	OM676081
hCoV-19/USA/WA-S18116/2022	Omicron	EPI_ISL_9881676	OM676122
hCoV-19/USA/WA-S17991/2022	Omicron	EPI_ISL_9881785	OM676047
hCoV-19/USA/WA-S18052/2022	Omicron	EPI_ISL_9881791	OM676084
hCoV-19/USA/WA-S18051/2022	Omicron	EPI_ISL_9881622	OM676083
hCoV-19/USA/WA-S17963/2022	Omicron	EPI_ISL_9881556	OM676019

hCoV-19/USA/WA-S17986/2022	Omicron	EPI_ISL_9881570	OM676042
hCoV-19/USA/WA-S18117/2022	Omicron	EPI_ISL_9881677	OM676123
hCoV-19/USA/WA-S18007/2022	Omicron	EPI_ISL_9881588	OM676053
hCoV-19/USA/WA-S17931/2022	Omicron	EPI_ISL_9881531	OM676000
hCoV-19/USA/WA-S17973/2022	Omicron	EPI_ISL_9881561	OM676029
hCoV-19/USA/WA-S17992/2022	Omicron	EPI_ISL_9881574	OM676048
hCoV-19/USA/WA-S18055/2022	Omicron	EPI_ISL_9881624	OM676087
hCoV-19/USA/WA-S17993/2022	Omicron	EPI_ISL_9881809	OM676049
hCoV-19/USA/WA-S17970/2022	Omicron	EPI_ISL_9881559	OM676026
hCoV-19/USA/WA-S17927/2022	Omicron	EPI_ISL_9881528	OM675996
hCoV-19/USA/WA-S17962/2022	Omicron	EPI_ISL_9881555	OM676018
hCoV-19/USA/WA-S17972/2022	Omicron	EPI_ISL_9881792	OM676028
hCoV-19/USA/WA-S18042/2022	Omicron	EPI_ISL_9881771	OM676074
hCoV-19/USA/WA-S18036/2022	Omicron	EPI_ISL_9881794	OM676068
hCoV-19/USA/WA-S18028/2022	Omicron	EPI_ISL_9881607	OM676060
hCoV-19/USA/WA-S18058/2022	Omicron	EPI_ISL_9881790	OM676090
hCoV-19/USA/WA-S18070/2022	Omicron	EPI_ISL_9881634	OM676102
hCoV-19/USA/WA-S18041/2022	Omicron	EPI_ISL_9881615	OM676073
hCoV-19/USA/WA-S18044/2022	Omicron	EPI_ISL_9881617	OM676076
hCoV-19/USA/WA-S18050/2022	Omicron	EPI_ISL_9881621	OM676082
hCoV-19/USA/WA-S18040/2022	Omicron	EPI_ISL_9881805	OM676072
hCoV-19/USA/WA-S18069/2022	Omicron	EPI_ISL_9881633	OM676101
hCoV-19/USA/WA-S18110/2022	Omicron	EPI_ISL_9881670	OM676119
hCoV-19/USA/WA-S18075/2022	Omicron	EPI_ISL_9881638	OM676107
hCoV-19/USA/WA-S18029/2022	Omicron	EPI_ISL_9881807	OM676061
hCoV-19/USA/WA-S18034/2022	Omicron	EPI_ISL_9881611	OM676066
hCoV-19/USA/WA-S18078/2022	Omicron	EPI_ISL_9881641	OM676110
hCoV-19/USA/WA-S18057/2022	Omicron	EPI_ISL_9881786	OM676089
hCoV-19/USA/WA-S18104/2022	Omicron	EPI_ISL_9881664	OM676113
hCoV-19/USA/WA-S18113/2022	Omicron	EPI_ISL_9881673	OM676121
hCoV-19/USA/WA-S18064/2022	Omicron	EPI_ISL_9881773	OM676096
hCoV-19/USA/WA-S18061/2022	Omicron	EPI_ISL_9881628	OM676093
hCoV-19/USA/WA-S18062/2022	Omicron	EPI_ISL_9881629	OM676094
hCoV-19/USA/WA-S18031/2022	Omicron	EPI_ISL_9881609	OM676063
hCoV-19/USA/WA-S18077/2022	Omicron	EPI_ISL_9881640	OM676109
hCoV-19/USA/WA-S18068/2022	Omicron	EPI_ISL_9881632	OM676100
hCoV-19/USA/WA-S18066/2022	Omicron	EPI_ISL_9881793	OM676098
hCoV-19/USA/WA-S18046/2022	Omicron	EPI_ISL_9881618	OM676078
hCoV-19/USA/WA-S18035/2022	Omicron	EPI_ISL_9881474	OM676067
hCoV-19/USA/WA-S18045/2022	Omicron	EPI_ISL_9881772	OM676077
hCoV-19/USA/WA-S18121/2022	Omicron	EPI_ISL_9881681	OM676125
hCoV-19/USA/WA-S18067/2022	Omicron	EPI_ISL_9881631	OM676099
hCoV-19/USA/WA-S18043/2022	Omicron	EPI_ISL_9881616	OM676075
hCoV-19/USA/WA-S18105/2022	Omicron	EPI_ISL_9881665	OM676114
hCoV-19/USA/WA-S18109/2022	Omicron	EPI_ISL_9881669	OM676118
hCoV-19/USA/WA-S18124/2022	Omicron	EPI_ISL_9881476	OM676128
hCoV-19/USA/WA-S18142/2022	Omicron	EPI_ISL_9881478	OM676146
hCoV-19/USA/WA-S18125/2022	Omicron	EPI_ISL_9881477	OM676129
hCoV-19/USA/WA-S18071/2022	Omicron	EPI_ISL_9881635	OM676103
hCoV-19/USA/WA-S18072/2022	Omicron	EPI_ISL_9881636	OM676104
hCoV-19/USA/WA-S18087/2022	Omicron	EPI_ISL_9881649	OM676111

hCoV-19/USA/WA-S18076/2022	Omicron	EPI_ISL_9881639	OM676108
hCoV-19/USA/WA-S18024/2022	Omicron	EPI_ISL_9881603	OM676056
hCoV-19/USA/WA-S18106/2022	Omicron	EPI_ISL_9881666	OM676115
hCoV-19/USA/WA-S18108/2022	Omicron	EPI_ISL_9881668	OM676117
hCoV-19/USA/WA-S18103/2022	Omicron	EPI_ISL_9881663	OM676112
hCoV-19/USA/WA-S18073/2022	Omicron	EPI_ISL_9881475	OM676105
hCoV-19/USA/WA-S18107/2022	Omicron	EPI_ISL_9881667	OM676116
hCoV-19/USA/WA-S18023/2022	Omicron	EPI_ISL_9881602	OM676055
hCoV-19/USA/WA-S18324/2022	Omicron	EPI_ISL_10570943	OM886453
hCoV-19/USA/WA-S18065/2022	Omicron	EPI_ISL_9881471	OM676097
hCoV-19/USA/WA-S18048/2022	Omicron	EPI_ISL_9881619	OM676080
hCoV-19/USA/WA-S18022/2022	Omicron	EPI_ISL_9881787	OM676054
hCoV-19/USA/WA-S18037/2022	Omicron	EPI_ISL_9881612	OM676069
hCoV-19/USA/WA-S18063/2022	Omicron	EPI_ISL_9881630	OM676095
hCoV-19/USA/WA-S18112/2022	Omicron	EPI_ISL_9881672	OM676120
hCoV-19/USA/WA-S18120/2022	Omicron	EPI_ISL_9881680	OM676124
hCoV-19/USA/WA-S18047/2022	Omicron	EPI_ISL_9881759	OM676079
hCoV-19/USA/WA-S18027/2022	Omicron	EPI_ISL_9881606	OM676059
hCoV-19/USA/WA-S18039/2022	Omicron	EPI_ISL_9881614	OM676071
hCoV-19/USA/WA-S18032/2022	Omicron	EPI_ISL_9881610	OM676064
hCoV-19/USA/WA-S18143/2022	Omicron	EPI_ISL_9881804	OM676147
hCoV-19/USA/WA-S18138/2022	Omicron	EPI_ISL_9881693	OM676142
hCoV-19/USA/WA-S18295/2022	Omicron	EPI_ISL_10570916	OM886444
hCoV-19/USA/WA-S18132/2022	Omicron	EPI_ISL_9881688	OM676136
hCoV-19/USA/WA-S18297/2022	Omicron	EPI_ISL_10570918	OM886446
hCoV-19/USA/WA-S18299/2022	Omicron	EPI_ISL_10570920	OM886448
hCoV-19/USA/WA-S18294/2022	Omicron	EPI_ISL_10570915	OM886443
hCoV-19/USA/WA-S18291/2022	Omicron	EPI_ISL_10570912	OM886440
hCoV-19/USA/WA-S18130/2022	Omicron	EPI_ISL_9881686	OM676134
hCoV-19/USA/WA-S18139/2022	Omicron	EPI_ISL_9881470	OM676143
hCoV-19/USA/WA-S18151/2022	Omicron	EPI_ISL_9881780	OM676155
hCoV-19/USA/WA-S18134/2022	Omicron	EPI_ISL_9881690	OM676138
hCoV-19/USA/WA-S18128/2022	Omicron	EPI_ISL_9881814	OM676132
hCoV-19/USA/WA-S18293/2022	Omicron	EPI_ISL_10570914	OM886442
hCoV-19/USA/WA-S18149/2022	Omicron	EPI_ISL_9881698	OM676153
hCoV-19/USA/WA-S18292/2022	Omicron	EPI_ISL_10570913	OM886441
hCoV-19/USA/WA-S18131/2022	Omicron	EPI_ISL_9881687	OM676135
hCoV-19/USA/WA-S18325/2022	Omicron	EPI_ISL_10570944	OM886454
hCoV-19/USA/WA-S18141/2022	Omicron	EPI_ISL_9881695	OM676145
hCoV-19/USA/WA-S18136/2022	Omicron	EPI_ISL_9881774	OM676140
hCoV-19/USA/WA-S18298/2022	Omicron	EPI_ISL_10570919	OM886447
hCoV-19/USA/WA-S18123/2022	Omicron	EPI_ISL_9881683	OM676127
hCoV-19/USA/WA-S18145/2022	Omicron	EPI_ISL_9881696	OM676149
hCoV-19/USA/WA-S18336/2022	Omicron	EPI_ISL_10570955	OM886465
hCoV-19/USA/WA-S18135/2022	Omicron	EPI_ISL_9881691	OM676139
hCoV-19/USA/WA-S18129/2022	Omicron	EPI_ISL_9881685	OM676133
hCoV-19/USA/WA-S18146/2022	Omicron	EPI_ISL_9881784	OM676150
hCoV-19/USA/WA-S18150/2022	Omicron	EPI_ISL_9881699	OM676154
hCoV-19/USA/WA-S18133/2022	Omicron	EPI_ISL_9881689	OM676137
hCoV-19/USA/WA-S18137/2022	Omicron	EPI_ISL_9881692	OM676141
hCoV-19/USA/WA-S18300/2022	Omicron	EPI_ISL_10570921	OM886449

hCoV-19/USA/WA-S18140/2022	Omicron	EPI_ISL_9881694	OM676144
hCoV-19/USA/WA-S18335/2022	Omicron	EPI_ISL_10570954	OM886464
hCoV-19/USA/WA-S18147/2022	Omicron	EPI_ISL_9881795	OM676151
hCoV-19/USA/WA-S18122/2022	Omicron	EPI_ISL_9881682	OM676126
hCoV-19/USA/WA-S18127/2022	Omicron	EPI_ISL_9881806	OM676131
hCoV-19/USA/WA-S18296/2022	Omicron	EPI_ISL_10570917	OM886445
hCoV-19/USA/WA-S18323/2022	Omicron	EPI_ISL_10570942	OM886452
hCoV-19/USA/WA-S18126/2022	Omicron	EPI_ISL_9881684	OM676130
hCoV-19/USA/WA-S18302/2022	Omicron	EPI_ISL_10570923	OM886451
hCoV-19/USA/WA-S18334/2022	Omicron	EPI_ISL_10570953	OM886463
hCoV-19/USA/WA-S18342/2022	Omicron	EPI_ISL_10570961	OM886466
hCoV-19/USA/WA-S18331/2022	Omicron	EPI_ISL_10570950	OM886460
hCoV-19/USA/WA-S18329/2022	Omicron	EPI_ISL_10570948	OM886458
hCoV-19/USA/WA-S18332/2022	Omicron	EPI_ISL_10570951	OM886461
hCoV-19/USA/WA-S18330/2022	Omicron	EPI_ISL_10570949	OM886459
hCoV-19/USA/WA-S18327/2022	Omicron	EPI_ISL_10570946	OM886456
hCoV-19/USA/WA-S18328/2022	Omicron	EPI_ISL_10570947	OM886457
hCoV-19/USA/WA-S18333/2022	Omicron	EPI_ISL_10570952	OM886462
hCoV-19/USA/WA-S18406/2022	Omicron	EPI_ISL_10571023	OM886479
hCoV-19/USA/WA-S18401/2022	Omicron	EPI_ISL_10571018	OM886474
hCoV-19/USA/WA-S18407/2022	Omicron	EPI_ISL_10571024	OM886480
hCoV-19/USA/WA-S18403/2022	Omicron	EPI_ISL_10571020	OM886476
hCoV-19/USA/WA-S18402/2022	Omicron	EPI_ISL_10571019	OM886475
hCoV-19/USA/WA-S18399/2022	Omicron	EPI_ISL_10571016	OM886472
hCoV-19/USA/WA-S18408/2022	Omicron	EPI_ISL_10571025	OM886481
hCoV-19/USA/WA-S18405/2022	Omicron	EPI_ISL_10571022	OM886478
hCoV-19/USA/WA-S18397/2022	Omicron	EPI_ISL_10571014	OM886470
hCoV-19/USA/WA-S18404/2022	Omicron	EPI_ISL_10571021	OM886477
hCoV-19/USA/WA-S18425/2022	Omicron	EPI_ISL_10571042	OM886490
hCoV-19/USA/WA-S18418/2022	Omicron	EPI_ISL_10571035	OM886483
hCoV-19/USA/WA-S18444/2022	Omicron	EPI_ISL_10571060	OM886508
hCoV-19/USA/WA-S18400/2022	Omicron	EPI_ISL_10571017	OM886473
hCoV-19/USA/WA-S18424/2022	Omicron	EPI_ISL_10571041	OM886489
hCoV-19/USA/WA-S18448/2022	Omicron	EPI_ISL_10571064	OM886512
hCoV-19/USA/WA-S18423/2022	Omicron	EPI_ISL_10571040	OM886488
hCoV-19/USA/WA-S18398/2022	Omicron	EPI_ISL_10571015	OM886471
hCoV-19/USA/WA-S18447/2022	Omicron	EPI_ISL_10571063	OM886511
hCoV-19/USA/WA-S18452/2022	Omicron	EPI_ISL_10571068	OM886515
hCoV-19/USA/WA-S18440/2022	Omicron	EPI_ISL_10571057	OM886505
hCoV-19/USA/WA-S18426/2022	Omicron	EPI_ISL_10571043	OM886491
hCoV-19/USA/WA-S18450/2022	Omicron	EPI_ISL_10571066	OM886513
hCoV-19/USA/WA-S18446/2022	Omicron	EPI_ISL_10571062	OM886510
hCoV-19/USA/WA-S18445/2022	Omicron	EPI_ISL_10571061	OM886509
hCoV-19/USA/WA-S18434/2022	Omicron	EPI_ISL_10571051	OM886499
hCoV-19/USA/WA-S18432/2022	Omicron	EPI_ISL_10571049	OM886497
hCoV-19/USA/WA-S18429/2022	Omicron	EPI_ISL_10571046	OM886494
hCoV-19/USA/WA-S18417/2022	Omicron	EPI_ISL_10571034	OM886482
hCoV-19/USA/WA-S18455/2022	Omicron	EPI_ISL_10571071	OM886518
hCoV-19/USA/WA-S18435/2022	Omicron	EPI_ISL_10571052	OM886500
hCoV-19/USA/WA-S18419/2022	Omicron	EPI_ISL_10571036	OM886484
hCoV-19/USA/WA-S18453/2022	Omicron	EPI_ISL_10571069	OM886516

hCoV-19/USA/WA-S17798/2022	Omicron	EPI_ISL_9638415	OM571581
hCoV-19/USA/WA-S18433/2022	Omicron	EPI_ISL_10571050	OM886498
hCoV-19/USA/WA-S18437/2022	Omicron	EPI_ISL_10571054	OM886502
hCoV-19/USA/WA-S18420/2022	Omicron	EPI_ISL_10571037	OM886485
hCoV-19/USA/WA-S18439/2022	Omicron	EPI_ISL_10571056	OM886504
hCoV-19/USA/WA-S17785/2022	Omicron	EPI_ISL_9638402	OM571568
hCoV-19/USA/WA-S18442/2022	Omicron	EPI_ISL_10571059	OM886507
hCoV-19/USA/WA-S18456/2022	Omicron	EPI_ISL_10571072	OM886519
hCoV-19/USA/WA-S18438/2022	Omicron	EPI_ISL_10571055	OM886503
hCoV-19/USA/WA-S18430/2022	Omicron	EPI_ISL_10571047	OM886495
hCoV-19/USA/WA-S18428/2022	Omicron	EPI_ISL_10571045	OM886493
hCoV-19/USA/WA-S18421/2022	Omicron	EPI_ISL_10571038	OM886486
hCoV-19/USA/WA-S17786/2022	Omicron	EPI_ISL_9638403	OM571569
hCoV-19/USA/WA-S18422/2022	Omicron	EPI_ISL_10571039	OM886487
hCoV-19/USA/WA-S18441/2022	Omicron	EPI_ISL_10571058	OM886506
hCoV-19/USA/WA-S18436/2022	Omicron	EPI_ISL_10571053	OM886501
hCoV-19/USA/WA-S18451/2022	Omicron	EPI_ISL_10571067	OM886514
hCoV-19/USA/WA-S18431/2022	Omicron	EPI_ISL_10571048	OM886496
hCoV-19/USA/WA-S18466/2022	Omicron	EPI_ISL_10571082	OM886529
hCoV-19/USA/WA-S17788/2022	Omicron	EPI_ISL_9638405	OM571571
hCoV-19/USA/WA-S18460/2022	Omicron	EPI_ISL_10571076	OM886523
hCoV-19/USA/WA-S18459/2022	Omicron	EPI_ISL_10571075	OM886522
hCoV-19/USA/WA-S17799/2022	Omicron	EPI_ISL_9638416	OM571582
hCoV-19/USA/WA-S17797/2022	Omicron	EPI_ISL_9638414	OM571580
hCoV-19/USA/WA-S17791/2022	Omicron	EPI_ISL_9638408	OM571574
hCoV-19/USA/WA-S17787/2022	Omicron	EPI_ISL_9638404	OM571570
hCoV-19/USA/WA-S17794/2022	Omicron	EPI_ISL_9638411	OM571577
hCoV-19/USA/WA-S18465/2022	Omicron	EPI_ISL_10571081	OM886528
hCoV-19/USA/WA-S17800/2022	Omicron	EPI_ISL_9638417	OM571583
hCoV-19/USA/WA-S18461/2022	Omicron	EPI_ISL_10571077	OM886524
hCoV-19/USA/WA-S17796/2022	Omicron	EPI_ISL_9638413	OM571579
hCoV-19/USA/WA-S18464/2022	Omicron	EPI_ISL_10571080	OM886527
hCoV-19/USA/WA-S17789/2022	Omicron	EPI_ISL_9638406	OM571572
hCoV-19/USA/WA-S18457/2022	Omicron	EPI_ISL_10571073	OM886520
hCoV-19/USA/WA-S17793/2022	Omicron	EPI_ISL_9638410	OM571576
hCoV-19/USA/WA-S18462/2022	Omicron	EPI_ISL_10571078	OM886525
hCoV-19/USA/WA-S17783/2022	Omicron	EPI_ISL_9638400	OM571566
hCoV-19/USA/WA-S17802/2022	Omicron	EPI_ISL_9638419	OM571585
hCoV-19/USA/WA-S18477/2022	Omicron	EPI_ISL_10571093	OM886540
hCoV-19/USA/WA-S17784/2022	Omicron	EPI_ISL_9638401	OM571567
hCoV-19/USA/WA-S18458/2022	Omicron	EPI_ISL_10571074	OM886521
hCoV-19/USA/WA-S17792/2022	Omicron	EPI_ISL_9638409	OM571575
hCoV-19/USA/WA-S17795/2022	Omicron	EPI_ISL_9638412	OM571578
hCoV-19/USA/WA-S17804/2022	Omicron	EPI_ISL_9638421	OM571587
hCoV-19/USA/WA-S17805/2022	Omicron	EPI_ISL_9638422	OM571588
hCoV-19/USA/WA-S17808/2022	Omicron	EPI_ISL_9638425	OM571591
hCoV-19/USA/WA-S17806/2022	Omicron	EPI_ISL_9638423	OM571589
hCoV-19/USA/WA-S18471/2022	Omicron	EPI_ISL_10571087	OM886534
hCoV-19/USA/WA-S18467/2022	Omicron	EPI_ISL_10571083	OM886530
hCoV-19/USA/WA-S18463/2022	Omicron	EPI_ISL_10571079	OM886526
hCoV-19/USA/WA-S18472/2022	Omicron	EPI_ISL_10571088	OM886535

hCoV-19/USA/WA-S18475/2022	Omicron	EPI_ISL_10571091	OM886538
hCoV-19/USA/WA-S18474/2022	Omicron	EPI_ISL_10571090	OM886537
hCoV-19/USA/WA-S17801/2022	Omicron	EPI_ISL_9638418	OM571584
hCoV-19/USA/WA-S18479/2022	Omicron	EPI_ISL_10571095	OM886542
hCoV-19/USA/WA-S17803/2022	Omicron	EPI_ISL_9638420	OM571586
hCoV-19/USA/WA-S18476/2022	Omicron	EPI_ISL_10571092	OM886539
hCoV-19/USA/WA-S18473/2022	Omicron	EPI_ISL_10571089	OM886536
hCoV-19/USA/WA-S18489/2022	Omicron	EPI_ISL_10571105	OM886552
hCoV-19/USA/WA-S18470/2022	Omicron	EPI_ISL_10571086	OM886533
hCoV-19/USA/WA-S17807/2022	Omicron	EPI_ISL_9638424	OM571590
hCoV-19/USA/WA-S18469/2022	Omicron	EPI_ISL_10571085	OM886532
hCoV-19/USA/WA-S18468/2022	Omicron	EPI_ISL_10571084	OM886531
hCoV-19/USA/WA-S18492/2022	Omicron	EPI_ISL_10571108	OM886555
hCoV-19/USA/WA-S18496/2022	Omicron	EPI_ISL_10571112	OM886559
hCoV-19/USA/WA-S18490/2022	Omicron	EPI_ISL_10571106	OM886553
hCoV-19/USA/WA-S18485/2022	Omicron	EPI_ISL_10571101	OM886548
hCoV-19/USA/WA-S18487/2022	Omicron	EPI_ISL_10571103	OM886550
hCoV-19/USA/WA-S18478/2022	Omicron	EPI_ISL_10571094	OM886541
hCoV-19/USA/WA-S18482/2022	Omicron	EPI_ISL_10571098	OM886545
hCoV-19/USA/WA-S18498/2022	Omicron	EPI_ISL_10571114	OM886561
hCoV-19/USA/WA-S18480/2022	Omicron	EPI_ISL_10571096	OM886543
hCoV-19/USA/WA-S18500/2022	Omicron	EPI_ISL_10571116	OM886563
hCoV-19/USA/WA-S18495/2022	Omicron	EPI_ISL_10571111	OM886558
hCoV-19/USA/WA-S18481/2022	Omicron	EPI_ISL_10571097	OM886544
hCoV-19/USA/WA-S18491/2022	Omicron	EPI_ISL_10571107	OM886554
hCoV-19/USA/WA-S18483/2022	Omicron	EPI_ISL_10571099	OM886546
hCoV-19/USA/WA-S18501/2022	Omicron	EPI_ISL_10571117	OM886564
hCoV-19/USA/WA-S18497/2022	Omicron	EPI_ISL_10571113	OM886560
hCoV-19/USA/WA-S18499/2022	Omicron	EPI_ISL_10571115	OM886562
hCoV-19/USA/WA-S18486/2022	Omicron	EPI_ISL_10571102	OM886549
hCoV-19/USA/WA-S18504/2022	Omicron	EPI_ISL_10571120	OM886567
hCoV-19/USA/WA-S18503/2022	Omicron	EPI_ISL_10571119	OM886566
hCoV-19/USA/WA-S18502/2022	Omicron	EPI_ISL_10571118	OM886565
hCoV-19/USA/WA-S18509/2022	Omicron	EPI_ISL_10571125	OM886572
hCoV-19/USA/WA-S18508/2022	Omicron	EPI_ISL_10571124	OM886571
hCoV-19/USA/WA-S18510/2022	Omicron	EPI_ISL_10571126	OM886573
hCoV-19/USA/WA-S18506/2022	Omicron	EPI_ISL_10571122	OM886569
hCoV-19/USA/WA-S18521/2022	Omicron	EPI_ISL_10571137	OM886584
hCoV-19/USA/WA-S18517/2022	Omicron	EPI_ISL_10571133	OM886580
hCoV-19/USA/WA-S18512/2022	Omicron	EPI_ISL_10571128	OM886575
hCoV-19/USA/WA-S18518/2022	Omicron	EPI_ISL_10571134	OM886581
hCoV-19/USA/WA-S18519/2022	Omicron	EPI_ISL_10571135	OM886582
hCoV-19/USA/WA-S18522/2022	Omicron	EPI_ISL_10571138	OM886585
hCoV-19/USA/WA-S18513/2022	Omicron	EPI_ISL_10571129	OM886576
hCoV-19/USA/WA-S18515/2022	Omicron	EPI_ISL_10571131	OM886578
hCoV-19/USA/WA-S18523/2022	Omicron	EPI_ISL_10571139	OM886586
hCoV-19/USA/WA-S18511/2022	Omicron	EPI_ISL_10571127	OM886574
hCoV-19/USA/WA-S18524/2022	Omicron	EPI_ISL_10571140	OM886587
hCoV-19/USA/WA-S18516/2022	Omicron	EPI_ISL_10571132	OM886579
hCoV-19/USA/WA-S18514/2022	Omicron	EPI_ISL_10571130	OM886577
hCoV-19/USA/WA-S18525/2022	Omicron	EPI_ISL_10571141	OM886588

hCoV-19/USA/WA-S18520/2022	Omicron	EPI_ISL_10571136	OM886583
hCoV-19/USA/WA-S18526/2022	Omicron	EPI_ISL_10571142	OM886589
hCoV-19/USA/WA-S18528/2022	Omicron	EPI_ISL_10571144	OM886591
hCoV-19/USA/WA-S18533/2022	Omicron	EPI_ISL_10571149	OM886596
hCoV-19/USA/WA-S18527/2022	Omicron	EPI_ISL_10571143	OM886590
hCoV-19/USA/WA-S18535/2022	Omicron	EPI_ISL_10571151	OM886598
hCoV-19/USA/WA-S18530/2022	Omicron	EPI_ISL_10571146	OM886593
hCoV-19/USA/WA-S18536/2022	Omicron	EPI_ISL_10571152	OM886599
hCoV-19/USA/WA-S18537/2022	Omicron	EPI_ISL_10571153	OM886600
hCoV-19/USA/WA-S18548/2022	Omicron	EPI_ISL_10571164	OM886611
hCoV-19/USA/WA-S18547/2022	Omicron	EPI_ISL_10571163	OM886610
hCoV-19/USA/WA-S18540/2022	Omicron	EPI_ISL_10571156	OM886603
hCoV-19/USA/WA-S18529/2022	Omicron	EPI_ISL_10571145	OM886592
hCoV-19/USA/WA-S18549/2022	Omicron	EPI_ISL_10571165	OM886612
hCoV-19/USA/WA-S18531/2022	Omicron	EPI_ISL_10571147	OM886594
hCoV-19/USA/WA-S18532/2022	Omicron	EPI_ISL_10571148	OM886595
hCoV-19/USA/WA-S18538/2022	Omicron	EPI_ISL_10571154	OM886601
hCoV-19/USA/WA-S18534/2022	Omicron	EPI_ISL_10571150	OM886597
hCoV-19/USA/WA-S18545/2022	Omicron	EPI_ISL_10571161	OM886608
hCoV-19/USA/WA-S18552/2022	Omicron	EPI_ISL_10571168	OM886615
hCoV-19/USA/WA-S18546/2022	Omicron	EPI_ISL_10571162	OM886609
hCoV-19/USA/WA-S18550/2022	Omicron	EPI_ISL_10571166	OM886613
hCoV-19/USA/WA-S18563/2022	Omicron	EPI_ISL_10571179	OM886626
hCoV-19/USA/WA-S18542/2022	Omicron	EPI_ISL_10571158	OM886605
hCoV-19/USA/WA-S18556/2022	Omicron	EPI_ISL_10571172	OM886619
hCoV-19/USA/WA-S18555/2022	Omicron	EPI_ISL_10571171	OM886618
hCoV-19/USA/WA-S18553/2022	Omicron	EPI_ISL_10571169	OM886616
hCoV-19/USA/WA-S18566/2022	Omicron	EPI_ISL_10571182	OM886629
hCoV-19/USA/WA-S18543/2022	Omicron	EPI_ISL_10571159	OM886606
hCoV-19/USA/WA-S18557/2022	Omicron	EPI_ISL_10571173	OM886620
hCoV-19/USA/WA-S18551/2022	Omicron	EPI_ISL_10571167	OM886614
hCoV-19/USA/WA-S18544/2022	Omicron	EPI_ISL_10571160	OM886607
hCoV-19/USA/WA-S18562/2022	Omicron	EPI_ISL_10571178	OM886625
hCoV-19/USA/WA-S18564/2022	Omicron	EPI_ISL_10571180	OM886627
hCoV-19/USA/WA-S18572/2022	Omicron	EPI_ISL_10571188	OM886634
hCoV-19/USA/WA-S18554/2022	Omicron	EPI_ISL_10571170	OM886617
hCoV-19/USA/WA-S18560/2022	Omicron	EPI_ISL_10571176	OM886623
hCoV-19/USA/WA-S18571/2022	Omicron	EPI_ISL_10571187	OM886633
hCoV-19/USA/WA-S18582/2022	Omicron	EPI_ISL_10571198	OM886643
hCoV-19/USA/WA-S18559/2022	Omicron	EPI_ISL_10571175	OM886622
hCoV-19/USA/WA-S18570/2022	Omicron	EPI_ISL_10571186	OM886632
hCoV-19/USA/WA-S18569/2022	Omicron	EPI_ISL_10571185	OP209798
hCoV-19/USA/WA-S18573/2022	Omicron	EPI_ISL_10571189	OM886635
hCoV-19/USA/WA-S18567/2022	Omicron	EPI_ISL_10571183	OM886630
hCoV-19/USA/WA-S18575/2022	Omicron	EPI_ISL_10571191	OM886637
hCoV-19/USA/WA-S18568/2022	Omicron	EPI_ISL_10571184	OM886631
hCoV-19/USA/WA-S18558/2022	Omicron	EPI_ISL_10571174	OM886621
hCoV-19/USA/WA-S18574/2022	Omicron	EPI_ISL_10571190	OM886636
hCoV-19/USA/WA-S18576/2022	Omicron	EPI_ISL_10571192	OM886638
hCoV-19/USA/WA-S18587/2022	Omicron	EPI_ISL_10571203	OM886648
hCoV-19/USA/WA-S18580/2022	Omicron	EPI_ISL_10571196	OM886641

hCoV-19/USA/WA-S18589/2022	Omicron	EPI_ISL_10571205	OM886650
hCoV-19/USA/WA-S18578/2022	Omicron	EPI_ISL_10571194	OM886640
hCoV-19/USA/WA-S18583/2022	Omicron	EPI_ISL_10571199	OM886644
hCoV-19/USA/WA-S18579/2022	Omicron	EPI_ISL_10571195	OP209799
hCoV-19/USA/WA-S18577/2022	Omicron	EPI_ISL_10571193	OM886639
hCoV-19/USA/WA-S18581/2022	Omicron	EPI_ISL_10571197	OM886642
hCoV-19/USA/WA-S18584/2022	Omicron	EPI_ISL_10571200	OM886645
hCoV-19/USA/WA-S18585/2022	Omicron	EPI_ISL_10571201	OM886646
hCoV-19/USA/WA-S18586/2022	Omicron	EPI_ISL_10571202	OM886647
hCoV-19/USA/WA-S18596/2022	Omicron	EPI_ISL_10571211	OM886656
hCoV-19/USA/WA-S18603/2022	Omicron	EPI_ISL_10571218	OM886663
hCoV-19/USA/WA-S18599/2022	Omicron	EPI_ISL_10571214	OM886659
hCoV-19/USA/WA-S18609/2022	Omicron	EPI_ISL_10571224	OM886669
hCoV-19/USA/WA-S18601/2022	Omicron	EPI_ISL_10571216	OM886661
hCoV-19/USA/WA-S18594/2022	Omicron	EPI_ISL_10571209	OM886654
hCoV-19/USA/WA-S18604/2022	Omicron	EPI_ISL_10571219	OM886664
hCoV-19/USA/WA-S18597/2022	Omicron	EPI_ISL_10571212	OM886657
hCoV-19/USA/WA-S18590/2022	Omicron	EPI_ISL_10571206	OM886651
hCoV-19/USA/WA-S18605/2022	Omicron	EPI_ISL_10571220	OM886665
hCoV-19/USA/WA-S18602/2022	Omicron	EPI_ISL_10571217	OM886662
hCoV-19/USA/WA-S18592/2022	Omicron	EPI_ISL_10571207	OM886652
hCoV-19/USA/WA-S18610/2022	Omicron	EPI_ISL_10571225	OM886670
hCoV-19/USA/WA-S18606/2022	Omicron	EPI_ISL_10571221	OM886666
hCoV-19/USA/WA-S18612/2022	Omicron	EPI_ISL_10571227	OM886672
hCoV-19/USA/WA-S18611/2022	Omicron	EPI_ISL_10571226	OM886671
hCoV-19/USA/WA-S18608/2022	Omicron	EPI_ISL_10571223	OM886668
hCoV-19/USA/WA-S18607/2022	Omicron	EPI_ISL_10571222	OM886667
hCoV-19/USA/WA-S18614/2022	Omicron	EPI_ISL_10571229	OM886674
hCoV-19/USA/WA-S18613/2022	Omicron	EPI_ISL_10571228	OM886673
hCoV-19/USA/WA-S18615/2022	Omicron	EPI_ISL_10571230	OM886675
hCoV-19/USA/WA-S18616/2022	Omicron	EPI_ISL_10571231	OM886676
hCoV-19/USA/WA-S18617/2022	Omicron	EPI_ISL_10571232	OM886677
hCoV-19/USA/WA-S18618/2022	Omicron	EPI_ISL_10571233	OM886678
hCoV-19/USA/WA-S18622/2022	Omicron	EPI_ISL_10571237	OM886682
hCoV-19/USA/WA-S18621/2022	Omicron	EPI_ISL_10571236	OM886681
hCoV-19/USA/WA-S18624/2022	Omicron	EPI_ISL_10571239	OM886684
hCoV-19/USA/WA-S18630/2022	Omicron	EPI_ISL_10571245	OM886690
hCoV-19/USA/WA-S18625/2022	Omicron	EPI_ISL_10571240	OM886685
hCoV-19/USA/WA-S18628/2022	Omicron	EPI_ISL_10571243	OM886688
hCoV-19/USA/WA-S18627/2022	Omicron	EPI_ISL_10571242	OM886687
hCoV-19/USA/WA-S18629/2022	Omicron	EPI_ISL_10571244	OM886689
hCoV-19/USA/WA-S18626/2022	Omicron	EPI_ISL_10571241	OM886686
hCoV-19/USA/WA-S18631/2022	Omicron	EPI_ISL_10571246	OM886691
hCoV-19/USA/WA-S18634/2022	Omicron	EPI_ISL_10571249	OM886694
hCoV-19/USA/WA-S18643/2022	Omicron	EPI_ISL_10571257	OM886701
hCoV-19/USA/WA-S18636/2022	Omicron	EPI_ISL_10571251	OM886696
hCoV-19/USA/WA-S18644/2022	Omicron	EPI_ISL_10571258	OM886702
hCoV-19/USA/WA-S18639/2022	Omicron	EPI_ISL_10571254	OM886698
hCoV-19/USA/WA-S18635/2022	Omicron	EPI_ISL_10571250	OM886695
hCoV-19/USA/WA-S18642/2022	Omicron	EPI_ISL_10571256	OM886700
hCoV-19/USA/WA-S18645/2022	Omicron	EPI_ISL_10571259	OM886703

hCoV-19/USA/WA-S18641/2022	Omicron	EPI_ISL_10571255	OM886699
hCoV-19/USA/WA-S18637/2022	Omicron	EPI_ISL_10571252	OP209800
hCoV-19/USA/WA-S18638/2022	Omicron	EPI_ISL_10571253	OM886697
